# Encapsulation of a Highly Acid-Stable Dicyano-Bodipy in Zr-Based Metal–Organic Frameworks with Increased Fluorescence Lifetime and Quantum Yield Within the Solid Solution Concept

**DOI:** 10.3390/molecules30214151

**Published:** 2025-10-22

**Authors:** Marcus N. A. Fetzer, Maximilian Vieten, Aysenur Limon, Christoph Janiak

**Affiliations:** Institut für Anorganische Chemie und Strukturchemie, Heinrich-Heine-Universität Düsseldorf, Universitätsstraße 1, D-40225 Düsseldorf, Germany; marcus.fetzer@hhu.de (M.N.A.F.); mavie107@hhu.de (M.V.); aysenur.limon@hhu.de (A.L.)

**Keywords:** dicyano-Bodipy, metal–organic framework (MOF), UiO-66, MOF-808, DUT-67, MIP-206, dye@MOF, fluorescence, solid solution

## Abstract

In this work, we have synthesized a more acid-stable variant of the classic chromophore difluoro-Bodipy by substituting the difluoro ligands at boron with cyano groups. This dicyano-Bodipy variant allowed the in situ incorporation during the MOF formation under acidic conditions and was investigated for the first time as dye@MOF composites using both post-synthetic and in situ incorporation into the zirconium-based metal–organic frameworks (MOFs) UiO-66, MOF-808, DUT-67, and MIP-206. The successful incorporation of dicyano-Bodipy was confirmed by PXRD, N_2_ sorption, digestion UV–Vis, and fluorescence spectroscopy. Depending on the incorporation method used, significant lower BET surface areas could be determined. The luminescence properties of the resulting dicyano-Bodipy@MOF composites from the in situ incorporation had up to almost eight-fold extended photoluminescent lifetimes of 9.0 ns, compared to the neat dye in its solid state with 1.2 ns, which suggests the formation of a solid solution in which the incorporated Bodipy is protected from external influences within a well-defined MOF pore. The quantum yield could be enhanced to as high as 77% through post-synthetic incorporation into the MOF DUT-67, compared to the neat dye in its solid state, with 9%.

## 1. Introduction

Metal–organic frameworks (MOFs) represent a unique class of hybrid compounds in which metal atoms or metal clusters as secondary building units (SBUs) are connected by organic linkers [1]. The resulting frameworks often exhibit high crystallinity and porosity, with specific surface areas of several thousand square meters per gram [2]. Compared to traditional porous materials, such as activated carbon, zeolites, or silica gel, the properties of MOFs, such as defined pores and pore sizes [3,4], surface areas, morphology [5,6,7,8], defects [9,10], or stability, can be designed by the use of different SBUs or organic linkers [11,12,13,14,15,16]. For these reasons, MOFs are investigated for a wide range of applications, such as heterogeneous catalysis [17,18], drug transport [19,20], gas and liquid adsorption and separation [21,22,23], heat transformation [24,25,26], or for the post-synthetic or in situ encapsulation of dyes to modulate their photophysical properties [27,28,29,30]. For example, Chen et al. were able to optimize the emission maximum of composites obtained by post-synthetic encapsulation of different rhodamine dyes in bio-MOF-1 [31]. Tang et al. produced white light-emitting composites by simultaneous encapsulation of different dyes in the MOF ZJU-28 [32]. An important aspect of dye encapsulation is the stability of the resulting composites. Zirconium-based MOFs have proven to be particularly robust compounds in this regard [33,34], making them suitable for the encapsulation of various dyes and their application in different fields. The class of Bodipy dyes is of particular interest in this context [35,36].

**Scheme 1:**
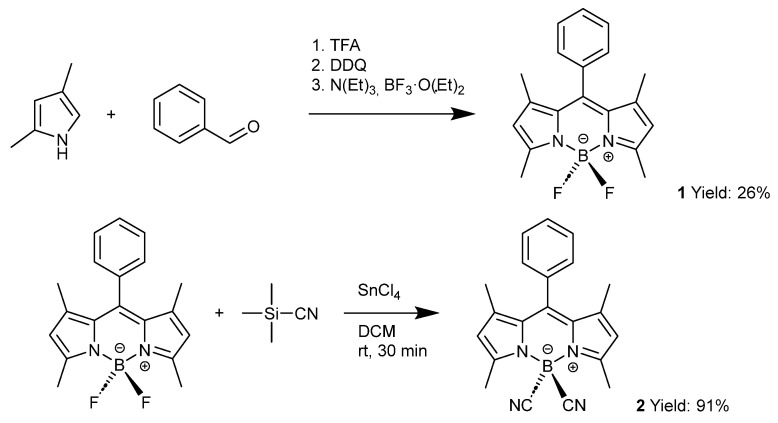
Synthesis of 5,5-difluoro-1,3,7,9-tetramethyl-10-phenyl-Bodipy **1** and 5,5-dicarbonitril-1,3,7,9-tetramethyl-10-phenyl-Bodipy **2**.

Bodipy is the abbreviation for boron dipyrromethene and was first synthesized and characterized by Treibs and Kreuzer in 1968 [37]. Bodipy represents a class of boron complexes formed by a dipyrromethene ligand and two other anionic ligands, usually fluorine (Scheme 1). Due to their chemical and photophysical properties, such as fluorescence with a high quantum yield and narrow emission band width, high thermal and photochemical stability, as well as good solubility in a wide range of solvents, interest in this class of dyes is constantly increasing [38,39]. It is widely used as a fluorescent sensor [40,41] in photothermal and photodynamic therapy for the treatment of cancer [42,43], as well as in the development of lasers [44] and for the labelling of proteins and nucleic acids [45]. It is also of great interest for catalysis research in various fields, such as selective oxidation or the coupling of a wide range of compounds [46,47]. In addition to the most widely used difluoro variant **1**, a large number of other Bodipy compounds have been prepared and analyzed in recent years by substitution of the fluorine atoms at the boron nucleus with other groups (Scheme 1) [48]. With several new dipyrromethene-based ligands, which form the backbone of the Bodipy compounds, the variety of different Bodipy compounds is constantly increasing, demonstrating the continuing interest in this class of dyes [49].

A problem with Bodipy dyes is that their exceptional photophysical properties are usually only observed in solution. In the solid state, strong intermolecular interactions such as π–π stacking and aggregation often lead to fluorescence quenching and spectral shifts. This limitation restricts their applicability in various fields that require stable and efficient solid-state emitters, such as LED technology or sensors. To preserve their luminescent properties in the solid state, formulation strategies such as the incorporation of Bodipy into solid matrices are necessary. We expect that the encapsulation or incorporation of Bodipy into MOFs will give the photophysical properties of the dye as in solution. This should enable us to produce composites for a wide range of applications, such as heterogeneous catalysis, cancer therapy, or sensing. Another interesting approach for such dye composites comes from LED technology, where interactions between the molecules, such as π-π stacking or aggregation-induced quenching, can be suppressed by targeted separation of the dye molecules in the individual MOF pores. The increased stability is highly beneficial for all types of applications. For these reasons, we present here the synthesis of a variety of zirconium-based Bodipy@MOF composites, both by post-synthetic and in situ encapsulation of Bodipy in the MOF.

## 2. Results and Discussion

The synthesis and analysis of the Bodipy dyes and the MOFs used in this work are described in detail in the Supplementary Materials. In short, the Bodipy syntheses in Scheme 1 were carried out according to Caruso et al. [50] and Nguyen et al. [51]. For the MOFs, we chose Zr-based MOFs because of their high hydrothermal stability compared to other MOFs. In addition, Zr-MOFs typically form colorless, crystalline compounds, which makes it easier to detect the color changes caused by the incorporation of Bodipy (see the in situ produced **2**@UiO-66 composites in Figure S13). The MOFs were prepared according to the synthesis described in the literature. For the synthesis of UiO-66, the procedure described by Katz et al. was used with minor modifications [52]. The MOFs MOF-808 and DUT-67 were both synthesized according to Reinsch et al. [53]. MIP-206 was prepared according to a procedure by Wang et al. [54].

The successful synthesis and identity of the Bodipy derivatives **1** and **2** were verified by NMR and high-resolution mass spectrometry. The neat MOFs were prepared by the synthesis routes reported in the literature and characterized by powder X-ray diffraction (PXRD) together with N_2_ gas sorption for BET surface area and porosity determination.

Initial encapsulation experiments with the zirconium-based MOFs UiO-66 and UiO-67, using the difluoro variant **1,** gave no detectable luminescence, probably due to the acidity of the inherently defective UiO structures [55]. This confirms the statement by Wang et al. that the dye cannot be effectively stabilized under strongly acidic conditions. In their study on the stability of Bodipy compounds, it was found that the addition of trifluoroacetic acid led to the degradation of the Bodipy structure [56]. Since the dicyanide variants exhibited increased stability under acidic conditions, we decided to use the less studied but more stable dicyano-Bodipy **2**. Another advantage of this dicyano-Bodipy **2** is, in addition to the increased stability against acids, the increased quantum yield compared to difluoro-Bodipy **1**. By going from the difluoro species **1** to the dicyano species **2**, the quantum yield of the Bodipy dye in THF solution could be increased from approximately 37% for **1** [57] to 89% for **2** [51].

**Figure 1:**
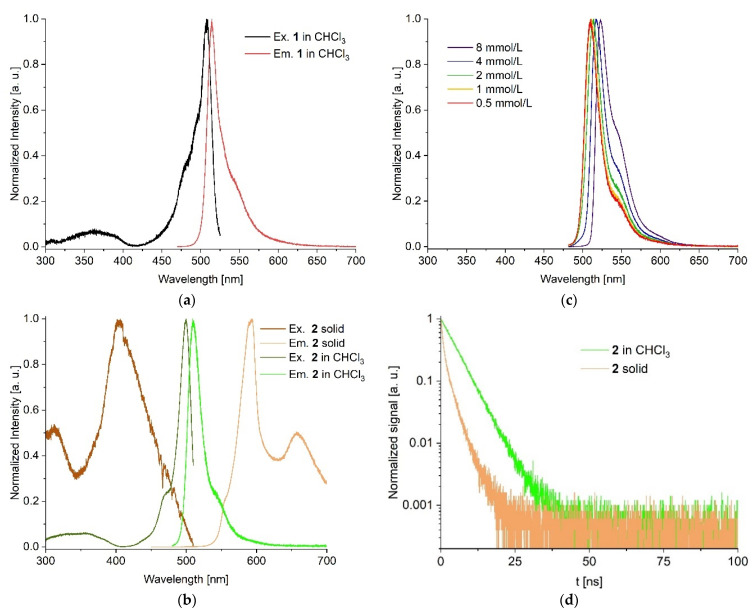
(**a**) Excitation and emission spectra of **1** in CHCl_3_. Emission spectra were measured with an excitation at 360 nm. Excitation spectra were measured at an emission of 540 nm. (**b**) Excitation and emission spectra of **2** in CHCl_3_ and neat Bodipy in the solid state in a reflective setup. Emission spectra in solution were measured with an excitation at 360 nm and as a solid with an excitation at 400 nm. Excitation spectra in solution were measured at an emission of 540 nm and as a solid at an emission of 600 nm. (**c**) Concentration-dependent bathochromic shift of **2** in CHCl_3_. Emission maxima: 510 nm (0.5 mmol/L), 511 nm (1 mmol/L), 514 nm (2 mmol/L), 517 nm (4 mmol/L), and 523 nm (8 mmol/L). Emission spectra were measured with an excitation at 360 nm. (**d**) Lifetime measurements of **2** in solution (0.5 mmol/L) and neat **2** in the solid state at the respective emission maxima of 510 nm and 594 nm (λ_exc_ = 375 nm).

Luminescence studies in solution show that both the difluoro and dicyano species **1** and **2** have narrow excitation and emission bands (Figure 1a,b) and are similar in their photophysical properties. The emission maxima at 514.4 nm for **1** and 510.0 nm for **2** in highly diluted chloroform solution correspond to the values given in the literature [51,57]. The Stokes shifts are 7.7 nm (295 cm^−1^) for **1** and 9.7 nm (381 cm^−1^) for **2,** and their full width at half-maximum (FWHM) values are 19 nm (720 cm^−1^) for **1** and 22 nm (850 cm^−1^) for **2**, making both species very narrow band emitters. Lifetime measurements of both compounds in chloroform yielded an average lifetime value of 5.7 ns for **1** and 4.8 ns for **2** (Figure S6, Supplementary Materials). Investigations of compound **2** in different solvents at the same concentration showed that the emission maxima are almost unaffected by the polarity of the solvent. All maxima were consistently in the range of 525 nm ± 2 nm (Figure S7, Supplementary Materials). This indicates that the photophysical behavior of compound **2** is largely independent of the solvent environment, suggesting minimal interaction between the solvent and the emission states of the compound. However, a concentration-dependent bathochromic shift of the emission maxima was observed in CHCl_3_ solution (Figure 1c).

The wavelength increases for the emission maxima with increasing concentration of compound **2,** which implies the presence of concentration-dependent aggregation phenomena such as π-π interactions and an associated reduction in free rotation. This assumption is also supported by the increasing intensity of the shoulder at 540 nm. These interactions can change the electronic environment of the emission states, which, in our case, leads to a bathochromic shift of the fluorescence.

As a solid, neat Bodipy **2** is a red crystalline powder, while the CHCl_3_ solution has a green color (Figure S8). We used a reflective setup for all solid-state measurements. For this purpose, both neat Bodipy in the solid state and all composites were placed as solids in a brass sample holder. Under UV light excitation with λ_exc_ = 400 nm, solid **2** emits an orange to red luminescence with emission maxima at 594 nm and 659 nm Figure 1b and Figure S8). The formation of two emission maxima and the clear bathochromic shift are discussed in the literature and can be attributed mainly to the formation of J-aggregates [58,59]. The excitation maximum for the solid occurs at 404 nm (Figure 1b), corresponding to Stokes shifts of 190 nm (7917 cm^−1^) and 255 nm (9577 cm^−1^) for the two emission peaks, respectively. A comparison of these values with those of the CHCl_3_ solution shows the influence of aggregation effects in **2**. While Bodipy molecules are surrounded by solvent molecules in solution, allowing for dynamic solvation, molecular motion, and minimal intermolecular interactions, they are densely packed in the solid state, promoting the formation of aggregates. These aggregates significantly influence molecular mobility, the local electronic environment, and the strength of intermolecular interactions. As a result, new electronic states with lower energy levels can arise in the solid state, often accompanied by enhanced π–π interactions between neighboring chromophores. These effects collectively lead to a bathochromic shift compared to the monomeric species in solution.

In addition to the clear differences in the steady-state luminescence measurements, there is also a significant change in luminescence lifetime and quantum yield for neat **2** in the solid state versus **2** in solution. Solid **2** had a lifetime of only 1.2 ns and an average quantum yield of only 9% compared to a lifetime of 4.8 ns in CHCl_3_ and an average quantum yield of 89% in THF solution (Figure 1d, Figures S6 and S9) [51]. The increased quantum yield of **2,** when going from the solid to the solution state, and the drastically improved stability against organic and inorganic acids, make compound **2** in solution very interesting for various applications. With the concept of solid solutions for dye@MOF composites, the embedding or encapsulation of non-aggregated Bodipy in the defined pores of MOFs could open up interesting future applications in areas such as optoelectronic devices or chemical sensors.

The inclusion of dyes in MOFs can be carried out post-synthetically by wet infiltration in the already prepared MOF or in situ, that is, during the MOF synthesis [60]. Furthermore, the presence of defects, such as missing linkers or missing cluster defects, can facilitate the incorporation of the dye molecules and enable the production of composites with both higher dye loadings and high N_2_ uptake. We chose the MOFs UiO-66, MOF-808, DUT-67, and MIP-206. All MOFs are colorless, microcrystalline powders with BET surface areas as reported in the literature. All of them show only a weak linker-based luminescence in the range between 350 and 440 nm, depending on the linkers used. We were able to perform both post-synthetic and in situ encapsulations for **2** in all MOFs except for MIP-206. For comparison purposes, the same initial concentration of Bodipy was used for both the post-synthetic and in situ approaches for MOF-808 and DUT-67.

### 2.1. Composite ***2***@UiO-66

**Figure 2:**
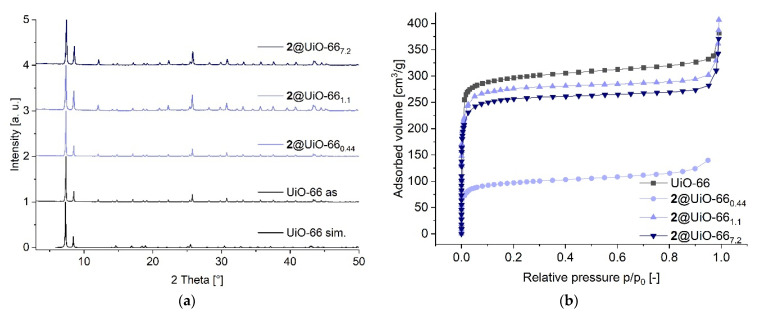
(**a**) PXRD of UiO-66, **2**@UiO-66_0.44_ (post-synthetic encapsulated) and the in situ encapsulated **2**@UiO-66_1.1_ and **2**@UiO-66_7.2_ composites. Simulated diffractogram of UiO-66 from CCDC No. 752051 [64]. (**b**) Nitrogen adsorption isotherms at 77 K of neat UiO-66 (1192 m^2^/g), **2**@UiO-66_1.1_ (1115 m^2^/g), **2**@UiO-66_7.2_ (916 m^2^/g), and **2**@UiO-66_0.44_ (381 m^2^/g). For the desorption isotherms, see Figures S11 and S12 in Supplementary Materials.

We started our studies on the encapsulation of **2** in MOFs with the zirconium MOF UiO-66. The encapsulation was carried out in a post-synthetic approach over a period of 5 days at a concentration of 1.0 mmol/L Bodipy in dichloromethane (CH_2_Cl_2_) with activated UiO-66 (see Section S5, Supplementary Materials for details). With pore sizes of about 8 Å for the tetrahedral pore and 11 Å for the octahedral pore in UiO-66, it is clear that the post-synthetic encapsulation of the Bodipy dye **2**, with molecular dimensions of about 10 Å, is restricted to the larger octahedral pores of UiO-66 (see Section S7, Supplementary Materials for details) [61,62]. Up to 0.44 wt% (4.4 mg of **2** per g of UiO-66 or 21.7 mmol of **2** per mol of UiO-66) could be embedded, based on the UiO-66 formula of [Zr_6_O_4_(OH)_4_(BDC)_6_] (1664.01 g/mol), denoting the composite as **2**@UiO-66_0.44_. No structural change could be detected by powder X-ray diffraction (PXRD, Figure 2a). The loading of all composites was determined post-synthetically by UV–Vis digestion analysis (Section S6, Supplementary Materials) [63] after extended washing procedures with dimethylformamide (DMF) until we could no longer detect any luminescence in the washing solution. This allowed us to assume that all Bodipy molecules attached to the outside of the MOF had been removed. The dye@MOF samples were then digested under strong basic (1 mol/L KOH for UiO-66, MOF-808, and MIP-206) or acidic conditions (conc. HCl for DUT-67) in order to determine the loading of **2** in the MOFs by UV–Vis spectroscopy. After the noted washing procedures, the thus determined Bodipy could only originate from the MOF-incorporated dye molecules.

The apparent low post-synthetic loading results from only a short diffusion path of the dye molecules into the crystal lattice, slightly beneath the surface layer [29]. This is in line with the significant decrease in the BET surface area from 1192 m^2^/g for neat UiO-66 down to 381 m^2^/g for **2**@UiO-66_0.44_ due to pore blocking (Figure 2b). Even these small amounts of **2** resulted in a composite with strong emission in the green wavelength range.

Further, the dye was also incorporated in situ into the MOF structure of UiO-66. Four different concentrations of **2** in DMF were used in the MOF reaction mixture of H_2_BDC and ZrCl_4_ (see Table S1, Supplementary Materials). Noteworthy, the synthesis of UiO-66 was carried out at 80 °C with hydrochloric acid as a modulator (see Section S5, Supplementary Materials for details) [52]. The improved stability of dicyano-Bodipy **2** versus difluoro-Bodipy **1** allowed us to use acids as modulators, giving acidic reaction conditions. The resulting **2**@UiO-66 composites were washed with DMF until the solution no longer showed any residual luminescence. The recorded PXRDs in Figure 2a show no differences compared to the reference sample or the simulation of neat UiO-66. This indicates that the presence of **2** does not affect the MOF structure, and face-centered cubic UiO-66 is the only crystalline product that is formed (see Figure S10, Supplementary Materials for all PXRDs). Scanning electron microscope (SEM) images of both the neat UiO-66 and the synthesized composites show a homogeneous distribution of MOF particles of similar size and unchanged morphology (see Figure S21, Supplementary Materials).

**Figure 3:**
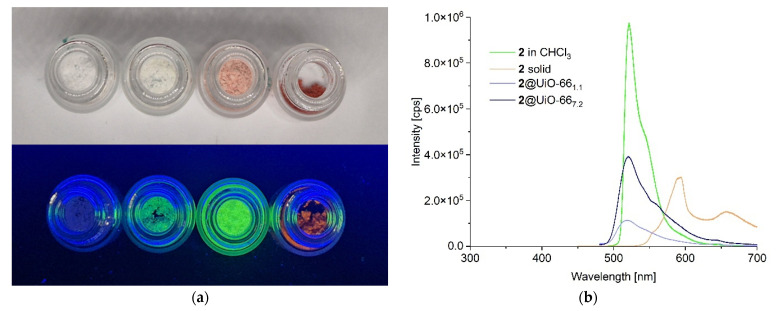
(**a**) From left to right: Neat UiO-66, post-synthetically encapsulated **2**@UiO-66_0.44_ (0.44 wt%), in situ prepared **2**@UiO-66_2.3_ (2.3 wt%), and neat **2** in the solid state, both under daylight (top) and under UV-light (λ_exc_ = 365 nm, bottom). (**b**) Emission spectra (λ_exc_ = 330 nm) of the **2**@UiO-66 in situ composites with the lowest (1.1 wt%) and highest (7.2 wt%) achieved loading together with the emission spectra of **2** in CHCl_3_ solution (8 mmol/L, λ_exc_ = 360 nm) and the emission spectra of neat **2** in the solid state (λ_exc_ = 400 nm) in a reflective setup.

The amount of Bodipy incorporated in situ in each composite was determined by UV–Vis digestion analysis (see above) between 1.1 and 7.2 wt% (11.1 to 77.5 mg of **2** per g of UiO-66 or 54.7 to 381 mmol of **2** per mol of UiO-66, see Section S6 for details), denoting the composites as **2**@UiO-66_1.1_ to **2**@UiO-66_7.2_, respectively. This corresponds to an average octahedral pore filling (n_av_(Bodipy/pore)) of 5.5 to 37.5%, taking into account the number of octahedral pores and asymmetric units in the unit cell of UiO-66 (For a detailed description of the pore calculation, see Section S7 in Supplementary Materials). A higher concentration of **2** in the reaction mixture resulted in a higher loading in the in situ method, also in comparison to the post-synthetic incorporation. While the low loading from post-synthetic encapsulation of **2** in UiO-66 still gave a colorless composite akin to the color of neat UiO-66, the higher amounts of **2** through in situ encapsulations were also evident from an increasingly pinkish color of **2**@UiO-66 (Figure 3a and Figure S13). 

Nitrogen sorption measurements and BET surface area determinations of the activated in situ **2**@UiO-66 composites listed in Table 1 showed a decrease in surface area with increasing Bodipy loading compared to neat UiO-66 (for all isotherms, see Figures S11 and S12). This trend supports the successful incorporation of the dye into the MOF structure, as higher loadings of Bodipy reduce the number of accessible pores available for N_2_ adsorption. The determined BET surface areas of the composites range from 1115 m^2^/g for the least in situ loaded composite **2**@UiO-66_1.1_ to 916 m^2^/g for the composite with the highest Bodipy loading **2**@UiO-66_7.2_. The even lower N_2_ uptake and concomitant lower surface area of only 381 m^2^/g for the post-synthetically prepared sample with only 0.44 wt% dye loading is interpreted through pore blocking by the dye molecules, which diffuse only slightly beneath the outer surface [29]. In situ incorporation results in a more homogeneous distribution of the Bodipy across the entire MOF. This generally leads to better pore accessibility and thus to a larger pore volume for in situ composites compared to post-synthetically produced composites. The similar or even slightly larger pore volume in the in situ composites compared to neat UiO-66 (Table 1) can be explained by the encapsulation of the Bodipy molecules and the resulting formation of defects due to the Bodipy molecules. These defects lead to larger pores with an expanded pore volume.

Photophysical measurements show that all **2**@UiO-66 composites exhibit a strong green emission, as seen for the CHCl_3_ solutions of **2** (Figure 3a and Figure S8), hence indicating solid solution behavior and is in contrast to the red emission characteristics of solid **2** (emission maxima at 594 nm and 659 nm, cf. Figure 1b). This behavior can be attributed to the spatial separation of individual Bodipy molecules within the porous structure of the MOF. Within the individual pores, the Bodipy molecules can behave more like molecules in solution than molecules in the solid state. The MOF framework effectively prevents the aggregation of Bodipy molecules in the solid and dry composite material, causing them to exhibit emission behavior similar to Bodipy in solution.

The **2**@UiO-66 composites exhibit emission maxima at 521 nm ± 2 nm and are thus all comparable to **2** in solution (8 mmol/L), which shows its emission maximum at 523 nm. Figure 3b illustrates the emission intensities of the lowest and highest in situ loaded **2**@UiO-66 composites together with **2** in CHCl_3_ (8 mmol/L) and **2** as a solid (for the emission spectra of all UiO-66 composites, see Figure S25). The observed broadening of the emission band of the composites shown in Figure 3b can be attributed to the heterogeneous and rigid environment of the MOF matrix. As already described, the Bodipy molecules in solution are uniformly solvated by CHCl_3_, which allows free rotational and translational motion. As a result, all molecules emit with nearly identical energies and produce a narrow emission band. In contrast, the confinement within the MOF restricts molecular mobility and exposes the Bodipy molecules to different microenvironments depending on their orientation within the pore. This leads to different emission energies and slight shifts in the emission maxima of individual molecules. In addition, intermolecular interactions such as aggregation further disrupt the energies in the excited state. The cumulative effect of all these changes leads to an experimentally observed broadening of the entire emission band. The intensity of this band increases with higher Bodipy loading, yet without any bathochromic shift being observed for higher concentrations in CHCl_3_ solutions of **2** (cf. Figure 1c). However, the emission maximum of 521 nm is already as seen for the higher-concentrated Bodipy CHCl_3_ solution of 8 mmol/L, which can be attributed to the suppression of the rotation of **2** within the MOF pore [66,67].

We assume that the compact structure of the MOF composites causes a primary inner filter effect, whereby the excitation light is absorbed before it reaches molecules located deeper within the framework. Consequently, these internal molecules remain unexcited. However, the observed linear correlation between emission intensity and dye loading and the absence of a significant redshift between the individual composites indicate that our composites are in a concentration range in which the secondary inner filter effect due to self-quenching or reabsorption phenomena plays a minor role, if any (see Figure S25f).

The constant emission maxima of the **2**@UiO-66 composites with different loadings strongly suggest that the encapsulation of **2** into the MOF structure effectively limits the interactions and aggregation of Bodipy molecules. All composites show, by their emission behavior, a desired separation of the Bodipy molecules, similar to that in the 8 mmol/L solution. However, it can be assumed that as the load increases, the probability of finding more than one Bodipy molecule in a pore also increases. This assumption is based on the shoulder at 550 nm, which is most prominent for the **2**@UiO-66_7.2_ composite with the highest loading. It indicates some formation of J-aggregates between two or more Bodipy molecules in the same pore. The emission maximum remains unchanged at this low degree of aggregation. Based on the above loading, we calculated the probability p of multiple occupations in a random distribution of Bodipy in UiO-66 and listed this probability in Table 2 (for a more detailed description of the calculation, see Section S7, Supplementary Materials). The same behavior was observed and discussed in the work of Püschel et al. [68].

The probability calculations confirmed our assumptions of some J-aggregate formation already at low loadings. At the same time, we were able to determine that the majority of the Bodipy molecules are embedded separately, which explains the solution-like luminescence behavior of the composites. Furthermore, based on the measured emission wavelengths and increasing intensities with loading, we assume that there is no strong interaction of the dye molecules with the MOF pore surface that would affect the wavelength of the emission maxima. Time-resolved fluorescence spectroscopy of the **2**@UiO-66 composites shows that a tri-exponential decay of the post-synthetically prepared **2**@UiO-66_0.44_ composite and bi-exponential decay of all in situ prepared composites were needed to describe the luminescence lifetimes and decays of all composites (for the decay plots of all UiO-66 composites, see Figure S26). The difference in decay can be explained by the presence of different luminescent species. These species can be attributed to the presence of different J-aggregates, which are formed by the multiple loading of pores with two or more Bodipy molecules within the MOF structure. This aggregation depends on the loading and the probability of several Bodipy molecules within a pore (cf. Table 2). Such an aggregate exhibits its own decay, whereby its respective properties significantly influence the lifetimes of the composite materials. Furthermore, the environment formed by the MOF can also affect the luminescence lifetime decay. This can result in either a prolongation or a shortening of the lifetime [69,70,71]. The lifetimes of **2**@UiO-66 were significantly extended compared to the lifetime of solid **2** (Table 3).

The average lifetimes τ_x_ of the composites are comparable to the lifetime of **2** in solution, with the lowest loadings of 0.44 and 1.1 wt% showing slightly increased lifetimes of 5.3 and 5.1 ns, respectively. Interestingly, the lifetimes show an inverse relationship to the Bodipy loading in the composites (Table 3). The sample with the highest loading of 7.2 wt% gave the shortest lifetime, measured at 4.0 ns. This trend is consistent with the observed quantum yields, which also decrease with increasing loading. In particular, the composites with the lowest loadings exhibited average quantum yields of around 30%, while the composite with the highest loading exhibited a reduced quantum yield of 19%. The presence of π-π interactions between neighboring Bodipy molecules can influence the lifetime. However, it is mainly explained by the presence and increase of J-aggregates in higher loaded composites (cf. Table 2) [72]. In addition to the increased aggregation, Bodipy-to-MOF wall interactions play a decisive role, as the excited state loses its energy more quickly through a Bodipy contact with the MOF pore wall. This can contribute to both a reduced quantum yield and a reduced lifetime. Although the quantum yields of **2**@UiO-66 are lower than the quantum yield of **2** in THF solution, they are significantly improved over the quantum yield of solid **2**, which is only 9%.

### 2.2. Composites ***2***@MOF-808, ***2***@DUT-67 and ***2***@MIP-206

To investigate the influence of the MOF host on the photophysical properties of **2**@MOF composites, a post-synthetic and an in situ composite were synthesized for MOF-808 and DUT-67, and a post-synthetic composite for MIP-206. The same MOF to Bodipy ratio was used in the preparation of both the post-synthetic and in situ composites. Due to the increased stability of **2**, we were able to use glacial acetic acid in a large excess in the in situ syntheses of the **2**@MOF-808 and **2**@DUT-67 composites. However, due to the extremely harsh synthesis conditions required for MIP-206, with formic acid as the sole solvent at temperatures of 180 °C, the in situ encapsulations of **2** could not be successfully carried out for MIP-206.

MOF-808 contains a hierarchical pore structure of large, interconnected hexagonal channels with diameters of approximately 18 Å (1.8 nm) and isolated tetrahedral cages with internal pore diameters of around 4.8 Å (or 0.48 nm); the latter being unaccessible for Bodipy [73]. DUT-67 has a hierarchical porous structure that contains cuboctahedral pores with a diameter of 14.2 Å (1.42 nm) and an octahedral pore with a diameter of 11.7 Å (1.17 nm) [74]; both of which can encapsulate Bodipy, and we took both pores into account when calculating pore filling. The structure of MIP-206 has uniform meso-voids with a diameter of ca. 26 Å (2.6 nm) [54].

**Figure 4:**
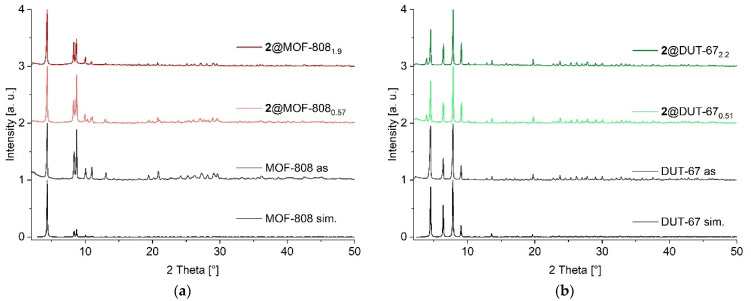
(**a**) PXRD of MOF-808, post-synthetic **2**@MOF-808_0.57_ and in situ encapsulated **2**@MOF-808_1.9_ composites. Simulated diffractogram of MOF-808 from CCDC No. 1002672 [73]. (**b**) PXRD of DUT-67, post-synthetic **2**@DUT-67_0.51_, and in situ encapsulated **2**@DUT-67_2.2_ composites. Simulated diffractogram of DUT-67 from CCDC No. 921644 [74]. For the PXRDs of MIP-206 and **2**@MIP-206_0.3_, see Figure S20a.

The post-synthetic **2**@MOF-808 and **2**@DUT-67 composites show no detectable changes in their PXRD patterns in Figure 4, indicating that the crystalline structures of the MOFs were unchanged.

From the UV–Vis digestion analysis, the post-synthetic loadings were determined to be 0.57 wt% for MOF-808 and 0.51 wt% for DUT-67 (see Table S2) [75,76]. The resulting composites are designated as **2**@MOF-808_0.57_ and **2**@DUT-67_0.51_. This corresponds to a molar ratio of 22.1 mmol Bodipy per mol of MOF-808 and 23.3 mmol Bodipy per mol of DUT-67. The formulas for MOF-808 [Zr_6_O_4_(OH)_10_(BTC)_2_(H_2_O)_6_] (1303.7 g/mol) and DUT-67 [Zr_6_O_4_(OH)_8_(TDC)_4_(H_2_O)_6_] (1536.1 g/mol) were used for the calculation [53].

**Figure 5:**
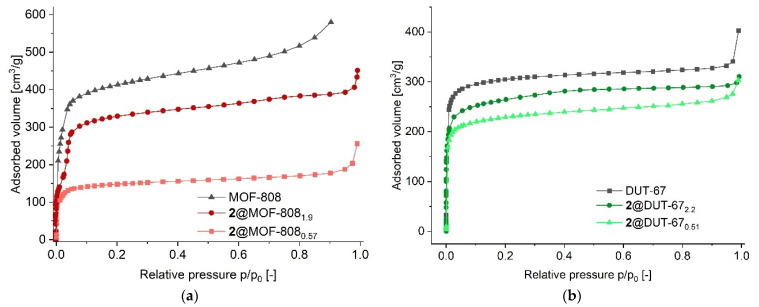
(**a**) Nitrogen adsorption isotherms at 77 K of neat MOF-808 (1683 m^2^/g), **2**@MOF-808_1.9_ (1200 m^2^/g, in situ), and **2**@MOF-808_0.57_ (596 m^2^/g, post-synthetic) composites. (**b**) Nitrogen adsorption isotherms at 77 K of neat DUT-67 (1207 m^2^/g), **2**@DUT-67_2.2_ (1020 m^2^/g, in situ), and **2**@DUT-67_0.51_ (840 m^2^/g, post-synthetic) composites. For the desorption isotherms, see Figures S18 and S19, for the isotherms of **2**@MIP-206_0.3_, see Figure S20b.

In agreement with such a low loading, we observed only very slight changes in the color of both post-synthetic composites, which were almost colorless, microcrystalline powders (see Section S10, Figures S27c and S29c). However, a significant decrease in the determined BET surface area was observed for the **2**@MOF-808_0.57_ composite, with a reduction of up to 65% versus neat MOF-808. By comparison, only a 31% reduction of the BET surface area was observed for the **2**@DUT-67_0.51_ composite compared to neat DUT-67 (Figure 5, Table 4).

The BET surface area reductions for the two post-synthetic composites compared to the in situ prepared composites (see below) can be attributed to the accumulation predominantly in the outer layers of the MOF crystallites rather than uniformly penetrating the entire porous network [29]. Thereby, access to the inner dye-free pore structure is restricted by pore blocking, which leads to reduced N_2_ adsorption and, consequently, lower BET surface areas.

The in situ encapsulations of **2** into MOF-808 and DUT-67 resulted in the composites **2**@MOF-808_1.9_ and **2**@DUT-67_2.2_ with loadings of 1.9 wt% and 2.2 wt%, corresponding to 74.7 and 102 mmol Bodipy per mol of MOF-808 and DUT-67, respectively. The in situ prepared composites **2**@MOF-808_1.9_ and **2**@DUT-67_2.2_ showed a slight color change to pale yellow compared to the neat MOFs (see Figures S27c and S29c, Supplementary Materials). The PXRD analysis of the in situ prepared composites, which are shown in Figure 4, revealed no differences from the simulated patterns of MOF-808 and DUT-67. These observations indicate the formation of the neat MOF structure and a crystalline phase-pure synthesis of the composites produced in situ. SEM images of neat MOF-808, DUT-67, and MIP-206, as well as the synthesized composites, each show a homogeneous distribution of MOF particles with a uniform size and unchanged morphology (see Figures S22–S24, Supplementary Materials).

Their N_2_ adsorption measurements, shown in Figure 5, yielded BET surface areas of 1200 m^2^/g for **2**@MOF-808_1.9_ and 1020 m^2^/g for **2**@DUT-67_2.2_. These values are comparable to those reported in the literature. We assume that the in situ encapsulation enables homogeneous distribution of the Bodipy dye within the MOF frameworks. This results in better pore availability compared to post-synthetic loading.

**Figure 6:**
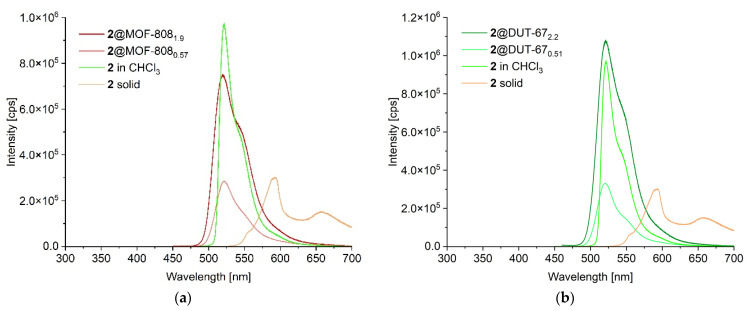
(**a**) Luminescence spectra of in situ and post-synthetic **2**@MOF-808 composites together with **2** in CHCl_3_ solution (8 mmol/L) and neat **2** in the solid state. (**b**) Luminescence spectra of in situ and post-synthetic **2**@DUT-67 composites together with **2** in CHCl_3_ solution (8 mmol/L) and neat **2** in the solid state. Emission spectra were measured with excitation at 360 nm for all composites and the CHCl_3_ solution and at 400 nm for neat **2** in the solid state in a reflective setup. For the luminescence spectra of post-synthetic **2**@MIP-206, see Figure S31a.

Steady-state luminescence measurements showed that both the post-synthetically loaded and in situ fabricated composites of MOF-808 and DUT-67 exhibit the characteristic luminescence of **2** in solution with emission maxima in the region of 521 nm ± 2 nm, with a shoulder at 550 nm (Figure 6). The observed broadening of the emission bands of the **2**@MOF-808 and **2**@DUT-67 composites shown in Figure 6 can be explained by the different microenvironments, as for the **2**@UiO-66 composites. Due to the higher loading in the composites produced in situ, a significant increase in luminescence intensity can be measured (for MIP-206 see Supplementary Materials, Section S10). We expect only a primary inner filter effect, just as for the **2**@UiO-66 composites, as the **2**@MOF-808 and **2**@DUT-67 composites are in the same concentration range. Again, as for **2**@UiO-66, the shoulder around 550 nm indicates multiple occupancy of some pores and thus the formation of aggregates between two or more Bodipy molecules in the same pore. The probability p of a multiple pore occupation of **2** in both MOFs is listed in Table 5, taking into account that the pores are formed with the molecular formula units of [Zr_6_O_4_(OH)_10_(BTC)_2_(H_2_O)_6_] for MOF-808 and [Zr_6_O_4_(OH)_8_(TDC)_4_(H_2_O)_6_] for DUT-67 (for a more detailed description of the structures and the ratios of pore-to-molecular formula units, see Supplementary Materials Section S7).

Looking at the lifetimes of the prepared composites in Table 6, the post-synthetic composites gave tri-exponential decays, and the in situ composites bi-exponential ones, as seen in the **2**@UiO-66 composites. The observed tri-exponential and bi-exponential decays suggest the coexistence of different emitting species—a property often associated in the literature with the formation of J-aggregates (cf. Table 5)—similar to what was seen in the **2**@UiO-66 composites [69]. Compared to the lifetime of **2** in solution (4.8 ns for 0.5 mmol/L) and as a solid (1.2 ns), all composites show significantly longer lifetimes. For the post-synthetic composites of MOF-808, DUT-67, and MIP-206, average lifetimes τ_x_ of 6.5 ns to 7.9 ns were measured. The in situ synthesized composites **2**@MOF-808_1.9_ and **2**@DUT-67_2.2_ showed a further significant increase in average lifetimes, with values of 9.0 ns and 8.5 ns, respectively (see Section S10, Supplementary Materials for the decay plots of all **2**@MOF composites). The average lifetime of these composites represents a doubling compared to **2** in solution (0.5 mmol/L) and an eight-fold increase compared to neat **2** in the solid state. For the **2**@UiO-66 composites, the average lifetime τ_x_ was close to the lifetime of **2** in solution (0.5 mmol/L) (cf. Table 3). 

Compared to the UiO-66 composites, the extended lifetimes in MOF-808 and DUT-67 reveal the influence of both pore size and pore environment. The latter two MOFs have pores that are significantly larger than the embedded dye molecules. This results in a larger distance and better separation of the Bodipy molecules within the pores and fewer interactions, such as π-π interactions or aggregation, both between individual Bodipy molecules and the pore walls and between neighboring Bodipy molecules. Lower interactions between both the Bodipy molecules themselves and the Bodipy molecules and the MOF walls are evident when comparing the aggregation (cf. Table 2 and Table 5) and respective lifetimes (cf. Table 3 and Table 6) of UiO-66 vs. MOF-808 and UiO-66 vs. DUT-67. At comparable degrees of aggregation of **2**@MOF-808_1.9_ (τ_x_ = 9.0 ns) vs. **2**@UiO-66_4.7_ (τ_x_ = 4.2 ns), and of **2**@DUT-67_2.2_ (τ_x_ = 8.5 ns) vs. **2**@UiO-66_2.3_ (τ_x_ = 4.6 ns), the lifetime increases with the pore size. The largest pore sizes in MOF-808 are about 18 Å, both in DUT-67 14 Å and in UiO-66 11 Å (see Section S7, Supplementary Materials). Thus, the distance between two Bodipy molecules as well as the distance between Bodipy molecules and the MOF walls also increases. In other words, the larger pores lead to weaker interactions of the Bodipy molecules, both with each other and with the MOF walls.

The presence of such monomers has already been described in the work of Xiong et al. using rhodamine B in ZIF-8 [70]. However, the significant increase in lifetime in the **2**@MOF-808 and **2**@DUT-67 composites compared to the lifetime in solution is not only due to the formation of such isolated monomers through enlarged pores, but also to the pore-forming MOF environment. Studies such as those by Liu et al. demonstrate the significant impact that changes in the MOF environment due to the introduction of functional groups can have on the photophysical properties of dyes [78]. In their work, they modified ZIF-8 by introducing 1,2,4-benzenetricarbonic acid with carboxyl functional groups, which significantly increased the quantum yield of the dye@MOF composite.

In addition to the extended lifetimes of **2** in **2**@MOF-808 and **2**@DUT-67, good quantum yields were observed. The composites prepared in situ had average quantum yields of 35% for MOF-808 and 41% for DUT-67. Significantly higher quantum yields were obtained for the post-synthetically encapsulated composites. For **2**@MOF-808_0.57_, we measured an average quantum yield of 48% and for **2**@DUT-67_0.51_, it was an impressive 77%. These high quantum yields derive from the Bodipy concentration in the outer MOF layer, which enables efficient molecule excitation. The inner Bodipy molecules of the in situ composites may not be reached by the photons, and their emission is also more readily absorbed within the MOF. Furthermore, the decreased dye-to-wall interactions between Bodipy in MOF-808 and DUT-67, with their larger pores, compared to UiO-66, result in less quenching. The quantum yield for **2**@DUT-67_0.51_ of 77% is comparable to the quantum yield of 89% for **2** in the THF solution reported in the literature [51].

All composites far exceed the quantum yield of the solid dye. The **2**@MOF-808 and **2**@DUT-67 composites also gave higher quantum yields than the **2**@UiO-66 composites. The UiO-66 composites ranged from 31% for **2**@UiO-66_1.1_, which had the highest quantum yield, to 19% for **2**@UiO-66_7.2_, which had the lowest quantum yield (cf. Table 3). Overall, the improved photophysical emission lifetimes and quantum yields make the Bodipy@MOF composites interesting for applications in solid-state devices.

## 3. Conclusions

In conclusion, we have shown that by substituting the well-known and well-studied difluoro groups of Bodipy with the less-investigated cyano groups, an acid-stable variant of Bodipy can be synthesized and used for both post-synthetic and in situ incorporation into zirconium-based MOFs under highly acidic synthesis conditions. The **2**@MOF composites show a distinct emission at 521 nm ± 2 nm, comparable to the emission of **2** in solution (8 mmol/L), in line with the solid solution concept for dyes@MOFs. From N_2_ adsorption measurements with surface area determinations, it could be inferred that the post-synthetic encapsulation method leads to a dye loading predominantly in the outer layers of the MOF crystallites. On the contrary, the in situ encapsulation method yields a more homogeneous distribution of the Bodipy molecules over the entire MOF crystallite. By means of distribution calculations and by fluorescence spectroscopic measurements, it is suggested that isolated J-aggregates are formed as the loadings increase. Yet, the post-synthetic concentration of the dye in the outer layer was found advantageous to increase the quantum yield in the case of large-pore MOFs. Through the choice of a MOF with a pore size larger than the dye molecule, composites with a high quantum yield close to the quantum yield of **2** in solution could be obtained due to decreased dye-to-wall interactions, and thereby, reduced quenching. The advantage of the solid **2**@MOF composites was their increased fluorescence lifetime and quantum yield, both up to eight-fold, compared to solid **2**. These results provide a starting point for the targeted development of solid Bodipy@MOF composites with specific optical and structural properties so that they could also be used for sensing as other dye@MOF composites [79,80,81].

## Figures and Tables

**Table 1:** Results of nitrogen sorption measurements for neat UiO-66 and **2**@UiO-66 composites.

Compound	Bodipy Loading [wt%] ^a^	*S*_BET_ [m^2^/g]	V_pore(total)_ [cm^3^/g] ^b^	V_pore(micro)_ [cm^3^/g] ^c^
UiO-66 literature	-	1580 [52]	-	-
	-	1175 [65]	0.63	-
UiO-66 synthesized	-	1192	0.55	0.55
**2**@UiO-66_0.44_ (post-synth.)	0.44	381	0.19	0.19
**2**@UiO-66_1.1_ (in situ)	1.1	1115	0.63	0.63
**2**@UiO-66_2.3_ (in situ)	2.3	1096	0.6	0.61
**2**@UiO-66_4.7_ (in situ)	4.7	1022	0.57	0.59
**2**@UiO-66_7.2_ (in situ)	7.2	916	0.56	0.53

^a^ Calculated by UV–Vis digestion analysis. ^b^ Total pore volume at p/p_0_ = 0.9. ^c^ Micropore volume calculated from NLDFT method using the N_2_ adsorption isotherm at 77 K for cylindrical pores ≤ 2 nm.

**Table 2:** Probability p of multiple occupations of Bodipy molecules in a pore of the **2**@UiO-66 composites ^a^.

	2@UiO-66_1.1_	2@UiO-66_2.3_	2@UiO-66_4.7_	2@UiO-66_7.2_
n_av_(Bodipy/pore)	0.055	0.12	0.243	0.375
p(one) [%]	96.4	94	86.7	78.1
p(two) [%]	3.6	5.7	12.1	18.4
p(≥three) [%]	0	0.2	1.1	3.5

^a^ Calculations are based on the UiO-66 formula of [Zr_6_O_4_(OH)_4_(BDC)_6_], the amount of Bodipy in mol per mol MOF, and the ratio of pores per formula unit (1:1). Pore refers to the octahedral pore in UiO-66.

**Table 3:** Photophysical data for **2** as a solid, in solution, and for the **2**@UiO-66 composites.

Compound	λ_F, max_ [nm] ^a^	τ_1_ (x_1_), τ_2_ (x_2_), τ_3_ (x_3_) [ns] ^b^	τ_x_ [ns] ^b^	Φ_F_ [%] ^c^
**2** solid	594/659	0.6 (0.74), 2.8 (0.29)	1.2	9
**2** in CHCl_3_ (0.5 mmol/L)	510	4.8 (1)	4.8	89 ^d^
**2**@UiO-66_0.44_ (post-synth.)	519	0.7 (0.10), 5.3 (0.8), 11.1 (0.09)	5.3	29
**2**@UiO-66_1.1_ (in situ)	521	1.0 (0.08), 5.4 (0.91)	5.1	32
**2**@UiO-66_2.3_ (in situ)	523	0.6 (0.09), 5.1 (0.89)	4.6	28
**2**@UiO-66_4.7_ (in situ)	519	1.4 (0.21); 4.8 (0.8)	4.2	21
**2**@UiO-66_7.2_ (in situ)	520	0.9 (0.14), 4.6 (0.85)	4.0	19

^a^ Wavelength of the fluorescence maximum; λ_exc_ = 400 nm for solid **2**; λ_exc_ = 360 nm for **2** in CHCl_3_; λ_exc_ = 330 nm for **2**@UiO-66. ^b^ Fluorescence lifetime (λ_exc_ = 375 nm): τ_i_ (x_i_) species i lifetime (fraction), τ_x_ species-weighted average lifetime. ^c^ Fluorescence quantum yield. ^d^ Measured in THF taken from the literature [51].

**Table 4:** Results of nitrogen sorption measurements for neat MOF-808, **2**@MOF-808 composites, neat DUT-67, **2**@DUT-67 composites, neat MIP-206, and **2**@MIP-206_0.3_.

Compound	Bodipy Loading [wt%] ^a^	*S*_BET_ [m^2^/g]	*V*_pore(total)_ [cm^3^/g] ^b^	*V*_pore(micro)_ [cm^3^/g] ^c^
MOF-808 literature	-	2060 [73]	0.84	-
	-	1210 [53]	0.53	-
MOF-808 synthesized	-	1683	0.8	0.76
**2**@MOF-808_0.57_ (post-synth.)	0.57	596	0.27	0.27
**2**@MOF-808_1.9_ (in situ)	1.9	1200	0.68	0.24
DUT-67 literature	-	1171 [77]	-	-
	-	1150 [53]	-	-
DUT-67 synthesized	-	1207	0.56	0.52
**2**@DUT-67_0.51_ (post-synth.)	0.51	840	0.4	0.37
**2**@DUT-67_2.2_ (in situ)	2.2	1020	0.47	0.51
MIP-206 literature	-	1059 [54]	0.45	-
MIP-206 synthesized	-	1062	0.42	0.41
**2**@MIP-206_0.3_ (post-synth.)	0.3	701	0.39	0.37

^a^ Calculated by UV–Vis digestion analysis. ^b^ Total pore volume at p/p_0_ = 0.9. ^c^ Micropore volume calculated from the NLDFT method using the N_2_ adsorption isotherm at 77 K for cylindrical pores ≤ 2 nm.

**Table 5:** Probability p of multiple occupations of **2** in the in situ prepared **2**@MOF-808 and **2**@DUT-67 composite materials ^a^.

	2@MOF-808_1.9_	2@DUT-67_2.2_
n_av_(Bodipy/pore)	0.298	0.155
p(one) [%]	83.4	91.7
p(two) [%]	14.7	7.7
p(≥three) [%]	1.9	0.6

^a^ The calculations are based on encapsulated 74.7 and 102 mmol Bodipy per mol of MOF-808 and DUT-67, respectively, and the ratio of pores per formula unit of each MOF. The latter ratio for MOF-808 is 0.25:1, and for DUT-67, it is 0.66:1. For more details about the calculation, see Supplementary Materials Section S7.

**Table 6:** Photophysical data for **2** as a solid and in solution, and the **2**@MOF-808 and **2**@DUT-67 composites.

Compound	λ_F, max_ [nm] *^a^*	τ_1_ (x_1_), τ_2_ (x_2_), τ_3_ (x_3_) [ns] *^b^*	τ_x_ [ns] *^b^*	Φ_F_ [%] *^c^*
**2** solid	594/659	0.6 (0.74), 2.8 (0.29)	1.2	9
**2** in CHCl_3_	510	4.8 (1)	4.8	89 ^*d*^
**2**@MOF-808_0.57_ (post-synth.)	523	6.5 (0.85), 0.8 (0.09), 13.2 (0.07)	6.5	48
**2**@MOF-808_1.9_ (in situ)	519	6.6 (0.44), 11.3 (0.54)	9.0	35
**2**@DUT-67_0.51_ (post-synth.)	519	6.2 (0.78), 1.6 (0.07), 10.8 (0.16)	6.8	77
**2**@DUT-67_2.2_ (in situ)	521	8.4 (0.97), 19.8 (0.02)	8.5	41

*^a^* Wavelength of the fluorescence maximum (λ_exc_ = 360 nm). *^b^* Fluorescence lifetime (λ_exc_ = 375 nm): τ_i_ (x_i_) species i lifetime (fraction), τ_x_ species-weighted average lifetime. *^c^* Fluorescence quantum yield. *^d^* Measured in THF taken from the literature [51]. For the fluorescence lifetime of post-synthetic **2**@MIP-206, see Table S6.

## Data Availability

The original contributions presented in this study are included in the article/Supplementary Material. Further inquiries can be directed to the corresponding author(s).

## Supplementary Materials

The following supporting information can be downloaded at: https://www.mdpi.com/article/10.3390/molecules30214151/s1, Section S1: General information; Section S2: Sources of chemicals; Section S3: Synthesis of Bodipy compounds **1** and **2** (Schemes S1 and S2); Section S4: Photophysical properties of Bodipy **2**; Section S5: Synthesis of UiO-66 and **2**@UiO-66 composites (Scheme S3); Section S6: Digestion UV–Vis; Section S7: Calculation of pore filling and the probability p of multiple occupations; Section S8: Synthesis of MOF-808, DUT-67, and MIP-206 and their composites (Schemes S4 and S5); Section S9: Scanning electron microscopy images of MOFs and composites; Section S10: Photophysical properties of all composites; Section S11: References. References [82,83] are cited in the Supplementary Materials.

## References

[1] Gangu K.K., Maddila S., Mukkamala S.B., Jonnalagadda S.B. (2016). A review on contemporary Metal–Organic Framework materials. Inorganica Chim. Acta.

[2] Honicke I.M., Senkovska I., Bon V., Baburin I.A., Bonisch N., Raschke S., Evans J.D., Kaskel S. (2018). Balancing Mechanical Stability and Ultrahigh Porosity in Crystalline Framework Materials. Angew. Chem. Int. Ed..

[3] Schaate A., Roy P., Godt A., Lippke J., Waltz F., Wiebcke M., Behrens P. (2011). Modulated synthesis of Zr-based metal-organic frameworks: From nano to single crystals. Chem. Eur. J..

[4] Ren J., Musyoka N.M., Langmi H.W., Segakweng T., North B.C., Mathe M., Kang X. (2014). Modulated synthesis of chromium-based metal-organic framework (MIL-101) with enhanced hydrogen uptake. Int. J. Hydrogen Energy.

[5] Bagherzadeh E., Zebarjad S.M., Hosseini H.R.M. (2018). Morphology Modification of the Iron Fumarate MIL—88A Metal–Organic Framework Using Formic Acid and Acetic Acid as Modulators. Eur. J. Inorg. Chem..

[6] Li M., Zhou H., Zhang L., Han J., Wang G., Fan F., Wang T., Zhang X., Fu Y. (2023). Size and morphology control of two-dimensional metal-organic frameworks through coordination modulation. Microporous Mesoporous Mater..

[7] Yang P., Huang Y., Zhang Z.W., Li N., Fan Y. (2020). Shape-controlled synthesis of the metal-organic framework MIL-125 towards a highly enhanced catalytic performance for the oxidative desulfurization of 4,6-dimethyldibenzothiophene. Dalton Trans..

[8] Liu Y., Liu S., He D., Li N., Ji Y., Zheng Z., Luo F., Liu S., Shi Z., Hu C. (2015). Crystal Facets Make a Profound Difference in Polyoxometalate-Containing Metal-Organic Frameworks as Catalysts for Biodiesel Production. J. Am. Chem. Soc..

[9] Cai G., Jiang H.L. (2017). A Modulator-Induced Defect-Formation Strategy to Hierarchically Porous Metal-Organic Frameworks with High Stability. Angew. Chem. Int. Ed..

[10] Epley C.C., Love M.D., Morris A.J. (2017). Characterizing Defects in a UiO-AZB Metal-Organic Framework. Inorg. Chem..

[11] Chung Y.G., Camp J., Haranczyk M., Sikora B.J., Bury W., Krungleviciute V., Yildirim T., Farha O.K., Sholl D.S., Snurr R.Q. (2014). Computation-Ready, Experimental Metal–Organic Frameworks: A Tool To Enable High-Throughput Screening of Nanoporous Crystals. Chem. Mater..

[12] Eddaoudi M., Kim J., Rosi N., Vodak D., Wachter J., O’Keeffe M., Yaghi O.M. (2002). Systematic design of pore size and functionality in isoreticular MOFs and their application in methane storage. Science.

[13] Yuan S., Feng L., Wang K., Pang J., Bosch M., Lollar C., Sun Y., Qin J., Yang X., Zhang P. (2018). Stable Metal-Organic Frameworks: Design, Synthesis, and Applications. Adv. Mater..

[14] Lu W., Wei Z., Gu Z.Y., Liu T.F., Park J., Park J., Tian J., Zhang M., Zhang Q., Gentle T. (2014). Tuning the structure and function of metal-organic frameworks via linker design. Chem. Soc. Rev..

[15] Mai Z., Liu D. (2019). Synthesis and Applications of Isoreticular Metal–Organic Frameworks IRMOFs-n (n = 1, 3, 6, 8). Cryst. Growth Des..

[16] Fan W., Zhang X., Kang Z., Liu X., Sun D. (2021). Isoreticular chemistry within metal–organic frameworks for gas storage and separation. Coord. Chem. Rev..

[17] Empel C., Fetzer M.N.A., Sasmal S., Strothmann T., Janiak C., Koenigs R.M. (2024). Unlocking catalytic potential: A rhodium(II)-based coordination polymer for efficient carbene transfer reactions with donor/acceptor diazoalkanes. Chem. Commun..

[18] Jin J., Wan S., Lee S., Oh C., Jang G.Y., Zhang K., Lu Z., Park J.H. (2023). Tailoring the Nanoporosity and Photoactivity of Metal-Organic Frameworks with Rigid Dye Modulators for Toluene Purification. Small.

[19] Vasconcelos I.B., Silva T.G.d., Militão G.C.G., Soares T.A., Rodrigues N.M., Rodrigues M.O., Costa N.B.d., Freire R.O., Junior S.A. (2012). Cytotoxicity and slow release of the anti-cancer drug doxorubicin from ZIF-8. RSC Adv..

[20] Javanbakht S., Pooresmaeil M., Namazi H. (2019). Green one-pot synthesis of carboxymethylcellulose/Zn-based metal-organic framework/graphene oxide bio-nanocomposite as a nanocarrier for drug delivery system. Carbohydr. Polym..

[21] Wu X., Bao Z., Yuan B., Wang J., Sun Y., Luo H., Deng S. (2013). Microwave synthesis and characterization of MOF-74 (M = Ni, Mg) for gas separation. Microporous Mesoporous Mater..

[22] Dechnik J., Nuhnen A., Janiak C. (2017). Mixed-Matrix Membranes of the Air-Stable MOF-5 Analogue [Co_4_(μ_4_-O)(Me_2_pzba)_3_] with a Mixed-Functional Pyrazolate-Carboxylate Linker for CO_2_/CH_4_ Separation. Cryst. Growth Des..

[23] Adil K., Belmabkhout Y., Pillai R.S., Cadiau A., Bhatt P.M., Assen A.H., Maurin G., Eddaoudi M. (2017). Gas/vapour separation using ultra-microporous metal-organic frameworks: Insights into the structure/separation relationship. Chem. Soc. Rev..

[24] Gökpinar S., Ernst S.-J., Hastürk E., Möllers M., El Aita I., Wiedey R., Tannert N., Nießing S., Abdpour S., Schmitz A. (2019). Air-Con Metal–Organic Frameworks in Binder Composites for Water Adsorption Heat Transformation Systems. Ind. Eng. Chem. Res..

[25] Hanikel N., Pei X., Chheda S., Lyu H., Jeong W., Sauer J., Gagliardi L., Yaghi O.M. (2021). Evolution of water structures in metal-organic frameworks for improved atmospheric water harvesting. Science.

[26] Terzis A., Ramachandran A., Wang K., Asheghi M., Goodson K.E., Santiago J.G. (2020). High-Frequency Water Vapor Sorption Cycling Using Fluidization of Metal-Organic Frameworks. Cell Rep. Phys. Sci..

[27] Zhang N., Zhang D., Zhao J., Xia Z. (2019). Fabrication of a dual-emitting dye-encapsulated metal-organic framework as a stable fluorescent sensor for metal ion detection. Dalton Trans..

[28] Knedel T.O., Buss S., Maisuls I., Daniliuc C.G., Schlusener C., Brandt P., Weingart O., Vollrath A., Janiak C., Strassert C.A. (2020). Encapsulation of Phosphorescent Pt(II) Complexes in Zn-Based Metal-Organic Frameworks toward Oxygen-Sensing Porous Materials. Inorg. Chem..

[29] Ma M., Gross A., Zacher D., Pinto A., Noei H., Wang Y., Fischer R.A., Metzler-Nolte N. (2011). Use of confocal fluorescence microscopy to compare different methods of modifying metal–organic framework (MOF) crystals with dyes. CrystEngComm.

[30] Yin J.C., Chang Z., Li N., He J., Fu Z.X., Bu X.H. (2020). Efficient Regulation of Energy Transfer in a Multicomponent Dye-Loaded MOF for White-Light Emission Tuning. ACS Appl. Mater. Interfaces.

[31] Chen W., Zhuang Y., Wang L., Lv Y., Liu J., Zhou T.L., Xie R.J. (2018). Color-Tunable and High-Efficiency Dye-Encapsulated Metal-Organic Framework Composites Used for Smart White-Light-Emitting Diodes. ACS Appl. Mater. Interfaces.

[32] Tang Y., Xia T., Song T., Cui Y., Yang Y., Qian G. (2018). Efficient Energy Transfer within Dyes Encapsulated Metal–Organic Frameworks to Achieve High Performance White Light—Emitting Diodes. Adv. Opt. Mater..

[33] He T., Kong X.J., Li J.R. (2021). Chemically Stable Metal-Organic Frameworks: Rational Construction and Application Expansion. Acc. Chem. Res..

[34] Kim J.Y., Kang J., Cha S., Kim H., Kim D., Kang H., Choi I., Kim M. (2024). Stability of Zr-Based UiO-66 Metal-Organic Frameworks in Basic Solutions. Nanomaterials.

[35] Glembockyte V., Frenette M., Mottillo C., Durantini A.M., Gostick J., Strukil V., Friscic T., Cosa G. (2018). Highly Photostable and Fluorescent Microporous Solids Prepared via Solid-State Entrapment of Boron Dipyrromethene Dyes in a Nascent Metal-Organic Framework. J. Am. Chem. Soc..

[36] Oh J.S., Park K.C., Gupta G., Lee C.Y. (2019). Complementary Chromophore Decoration in NU-1000 via Solvent-Assisted Ligands Incorporation: Efficient Energy Transfer within the Metal-Organic Frameworks. Bull. Korean Chem. Soc..

[37] Treibs A., Kreuzer F.H. (1968). Difluorboryl-Komplexe von Di- und Tripyrrylmethenen. Justus Liebigs Ann. Chem..

[38] Duan C., Zhou Y., Shan G.-G., Chen Y., Zhao W., Yuan D., Zeng L., Huang X., Niu G. (2019). Bright solid-state red-emissive BODIPYs: Facile synthesis and their high-contrast mechanochromic properties. J. Mater. Chem. C.

[39] Gibbs J.H., Robins L.T., Zhou Z., Bobadova-Parvanova P., Cottam M., McCandless G.T., Fronczek F.R., Vicente M.G. (2013). Spectroscopic, computational modeling and cytotoxicity of a series of meso-phenyl and meso-thienyl-BODIPYs. Bioorganic Med. Chem..

[40] Tang F.K., Zhu J., Kong F.K., Ng M., Bian Q., Yam V.W., Tse A.K., Tse Y.C., Leung K.C. (2020). A BODIPY-based fluorescent sensor for the detection of Pt2+ and Pt drugs. Chem. Commun..

[41] Dorh N., Zhu S., Dhungana K.B., Pati R., Luo F.T., Liu H., Tiwari A. (2015). BODIPY-Based Fluorescent Probes for Sensing Protein Surface-Hydrophobicity. Sci. Rep..

[42] Guan Q., Fu D.D., Li Y.A., Kong X.M., Wei Z.Y., Li W.Y., Zhang S.J., Dong Y.B. (2019). BODIPY-Decorated Nanoscale Covalent Organic Frameworks for Photodynamic Therapy. iScience.

[43] Schneider L., Kalt M., Koch S., Sithamparanathan S., Villiger V., Mattiat J., Kradolfer F., Slyshkina E., Luber S., Bonmarin M. (2023). BODIPY-Based Photothermal Agents with Excellent Phototoxic Indices for Cancer Treatment. J. Am. Chem. Soc..

[44] Mack J., Kubheka G., May A., Ngoy B.P., Nyokong T. (2024). BODIPY dyes for optical limiting applications on the nanosecond timescale. Dalton Trans..

[45] Karaman O., Almammadov T., Emre Gedik M., Gunaydin G., Kolemen S., Gunbas G. (2019). Mitochondria-Targeting Selenophene-Modified BODIPY-Based Photosensitizers for the Treatment of Hypoxic Cancer Cells. ChemMedChem.

[46] Wang X.F., Yu S.S., Wang C., Xue D., Xiao J. (2016). BODIPY catalyzed amide synthesis promoted by BHT and air under visible light. Org. Biomol. Chem..

[47] Liras M., Iglesias M., Sánchez F. (2016). Conjugated Microporous Polymers Incorporating BODIPY Moieties as Light-Emitting Materials and Recyclable Visible-Light Photocatalysts. Macromolecules.

[48] Stachelek P., Alsimaree A.A., Alnoman R.B., Harriman A., Knight J.G. (2017). Thermally-Activated, Delayed Fluorescence in O,B,O- and N,B,O-Strapped Boron Dipyrromethene Derivatives. J. Phys. Chem. A.

[49] Fan Y., Zhang J., Hong Z., Qiu H., Li Y., Yin S. (2020). Architectures and Applications of BODIPY-Based Conjugated Polymers. Polymers.

[50] Caruso E., Gariboldi M., Sangion A., Gramatica P., Banfi S. (2017). Synthesis, photodynamic activity, and quantitative structure-activity relationship modelling of a series of BODIPYs. J. Photochem. Photobiol. B.

[51] Nguyen A.L., Wang M., Bobadova-Parvanova P., Do Q., Zhou Z., Fronczek F.R., Smith K.M., Vicente M.G.H. (2016). Synthesis and properties ofB-cyano-BODIPYs. J. Porphyr. Phthalocyanines.

[52] Katz M.J., Brown Z.J., Colon Y.J., Siu P.W., Scheidt K.A., Snurr R.Q., Hupp J.T., Farha O.K. (2013). A facile synthesis of UiO-66, UiO-67 and their derivatives. Chem. Commun..

[53] Reinsch H., Waitschat S., Chavan S.M., Lillerud K.P., Stock N. (2016). A Facile “Green” Route for Scalable Batch Production and Continuous Synthesis of Zirconium MOFs. Eur. J. Inorg. Chem..

[54] Wang S., Chen L., Wahiduzzaman M., Tissot A., Zhou L., Ibarra I.A., Gutiérrez-Alejandre A., Lee J.S., Chang J.-S., Liu Z. (2021). A Mesoporous Zirconium-Isophthalate Multifunctional Platform. Matter.

[55] Jiang J., Yaghi O.M. (2015). Bronsted acidity in metal-organic frameworks. Chem. Rev..

[56] Wang M., Vicente M.G.H., Mason D., Bobadova-Parvanova P. (2018). Stability of a Series of BODIPYs in Acidic Conditions: An Experimental and Computational Study into the Role of the Substituents at Boron. ACS Omega.

[57] Zhang G., Wang M., Fronczek F.R., Smith K.M., Vicente M.G.H. (2018). Lewis-Acid-Catalyzed BODIPY Boron Functionalization Using Trimethylsilyl Nucleophiles. Inorg. Chem..

[58] Manzano H., Esnal I., Marqués-Matesanz T., Bañuelos J., López-Arbeloa I., Ortiz M.J., Cerdán L., Costela A., García-Moreno I., Chiara J.L. (2016). Unprecedented J-Aggregated Dyes in Pure Organic Solvents. Adv. Funct. Mater..

[59] Marfin Y.S., Banakova E.A., Merkushev D.A., Usoltsev S.D., Churakov A.V. (2020). Effects of Concentration on Aggregation of BODIPY-Based Fluorescent Dyes Solution. J. Fluoresc..

[60] Ryu U., Lee H.S., Park K.S., Choi K.M. (2018). The rules and roles of metal-organic framework in combination with molecular dyes. Polyhedron.

[61] Slawek A., Jajko G., Ogorzaly K., Dubbeldam D., Vlugt T.J.H., Makowski W. (2022). The Influence of UiO-66 Metal-Organic Framework Structural Defects on Adsorption and Separation of Hexane Isomers. Chem. Eur. J..

[62] Bárcia P.S., Guimarães D., Mendes P.A.P., Silva J.A.C., Guillerm V., Chevreau H., Serre C., Rodrigues A.E. (2011). Reverse shape selectivity in the adsorption of hexane and xylene isomers in MOF UiO-66. Microporous Mesoporous Mater..

[63] D’Amato R., Bondi R., Moghdad I., Marmottini F., McPherson M.J., Naïli H., Taddei M., Costantino F. (2021). “Shake ‘n Bake” Route to Functionalized Zr-UiO-66 Metal-Organic Frameworks. Inorg. Chem..

[64] Guillerm V., Gross S., Serre C., Devic T., Bauer M., Ferey G. (2010). A zirconium methacrylate oxocluster as precursor for the low-temperature synthesis of porous zirconium(IV) dicarboxylates. Chem. Commun..

[65] Shearer G.C., Chavan S., Bordiga S., Svelle S., Olsbye U., Lillerud K.P. (2016). Defect Engineering: Tuning the Porosity and Composition of the Metal–Organic Framework UiO-66 via Modulated Synthesis. Chem. Mater..

[66] Grabowski Z.R., Dobkowski J. (1983). Twisted Intramolecular Charge-Transfer (Tict) Excited-States—Energy and Molecular-Structure. Pure Appl. Chem..

[67] Sasaki S., Drummen G.P.C., Konishi G. (2016). Recent advances in twisted intramolecular charge transfer (TICT) fluorescence and related phenomena in materials chemistry. J. Mater. Chem. C.

[68] Puschel D., Hede S., Maisuls I., Hofert S.P., Woschko D., Kuhnemuth R., Felekyan S., Seidel C.A.M., Czekelius C., Weingart O. (2023). Enhanced Solid-State Fluorescence of Flavin Derivatives by Incorporation in the Metal-Organic Frameworks MIL-53(Al) and MOF-5. Molecules.

[69] Rodrigues A.C.B., Wetterling D., Scherf U., Seixas de Melo J.S. (2021). Tuning J-aggregate Formation and Emission Efficiency in Cationic Diazapentacenium Dyes. Chem. Eur. J..

[70] Xiong T., Zhang Y., Amin N., Tan J.C. (2021). A Luminescent Guest@MOF Nanoconfined Composite System for Solid-State Lighting. Molecules.

[71] Gutierrez M., Zhang Y., Tan J.C. (2022). Confinement of Luminescent Guests in Metal-Organic Frameworks: Understanding Pathways from Synthesis and Multimodal Characterization to Potential Applications of LG@MOF Systems. Chem. Rev..

[72] Schrimpf W., Jiang J.C., Ji Z., Hirschle P., Lamb D.C., Yaghi O.M., Wuttke S. (2018). Chemical diversity in a metal-organic framework revealed by fluorescence lifetime imaging. Nat. Commun..

[73] Furukawa H., Gandara F., Zhang Y.B., Jiang J., Queen W.L., Hudson M.R., Yaghi O.M. (2014). Water adsorption in porous metal-organic frameworks and related materials. J. Am. Chem. Soc..

[74] Bon V., Senkovska I., Baburin I.A., Kaskel S. (2013). Zr- and Hf-Based Metal–Organic Frameworks: Tracking Down the Polymorphism. Cryst. Growth Des..

[75] Valverde A., Tovar G.I., Rio-López N.A., Torres D., Rosales M., Wuttke S., Fidalgo-Marijuan A., Porro J.M., Jiménez-Ruiz M., García Sakai V. (2022). Designing Metal-Chelator-like Traps by Encoding Amino Acids in Zirconium-Based Metal–Organic Frameworks. Chem. Mater..

[76] Winters W.M.W., Zhou C., Hou J., Diaz-Lopez M., Bennett T.D., Yue Y. (2024). Order-to-Disorder Transition in a Zirconium-Based Metal–Organic Framework. Chem. Mater..

[77] Drache F., Bon V., Senkovska I., Marschelke C., Synytska A., Kaskel S. (2016). Postsynthetic Inner-Surface Functionalization of the Highly Stable Zirconium-Based Metal-Organic Framework DUT-67. Inorg. Chem..

[78] Liu Q., Chen X., Wu J., Zhang L., He G., Tian S., Zhao X. (2023). Enhanced Luminescence of Dye-Decorated ZIF-8 Composite Films via Controllable D-A Interactions for White Light Emission. Langmuir.

[79] Yu J., Cheng Y., Zhang X., Zhou L., Song Z., Nezamzadeh-Ejhieh A., Huang Y. (2025). Application progress of nano-platforms based on metal-organic frameworks (MOFs) in modern agriculture. J. Environ. Chem. Eng..

[80] Zhang Y., Tan H., Zhu J., Duan L., Ding Y., Liang F., Li Y., Peng X., Jiang R., Yu J. (2024). A Fluorine-Functionalized Tb(III)-Organic Framework for Ba^2+^ Detection. Molecules.

[81] Ma D., Chen C., Chen M., Zhu S., Wu Y., Li Z., Li Y., Zhou L. (2019). A hydrostable Cadmium-Organic Framework for Highly Selective and Sensitive Luminescence Sensing of Al^3+^ Ion. J. Inorg. Organomet. Polym. Mater..

[82] Valenzano L., Civalleri B., Chavan S., Bordiga S., Nilsen M.H., Jakobsen S., Lillerud K.P., Lamberti C. (2011). Disclosing the Complex Structure of UiO-66 Metal Organic Framework: A Synergic Combination of Experiment and Theory. Chem. Mater..

[83] Garai M., Yavuz C.T. (2021). Robust Mesoporous Zr-MOF with Pd Nanoparticles for Formic-Acid-Based Chemical Hydrogen Storage. Matter.

