# Supplementary material for: Encapsulation of a Highly Acid-Stable Dicyano-Bodipy in Zr-Based Metal–Organic Frameworks with Increased Fluorescence Lifetime and Quantum Yield Within the Solid Solution Concept
Source: Molecules. 2025 Oct 22;30(21):4151. doi: 10.3390/molecules30214151 (PMC12611095; doi:10.3390/molecules30214151)
Supplement: Supplementary file 1 [file molecules-30-04151-s001.zip › molecules-3921101-supplementary.pdf]

Supplementary Material

Encapsulation of a Highly Acid-stable Dicyano-Bodipy in Zr-based Metal–organic Frameworks with Increased Fluorescence Lifetime and Quantum Yield within the Solid solution Concept

Marcus N. A. Fetzer, Maximilian Vieten, Aysenur Limon, and Christoph Janiak*

Institut für Anorganische Chemie und Strukturchemie, Heinrich-Heine-Universität Düsseldorf, 40204 Düsseldorf, Germany

E-Mail: Christoph Janiak* - janiak@uni-duesseldorf.de * Corresponding author

Fax: +49-211-81-12287; Tel: +49-211-81-12286

Emails: Marcus N. A. Fetzer – fetzer@uni-düsseldorf.de; Maximilian Vieten – mavie107@uni-düsseldorf.de; Aysenur Limon – aysenur.limon@uni-düsseldorf.de

Table of Contents

1. General information 2
2. Sources of chemicals 3
3. Synthesis of Bodipy compounds **1** and **2** (Scheme S1 and S2) 4
4. Photophysical properties of Bodipy **2** 9
5. Synthesis of UiO-66 and 2@UiO-66 composites (Scheme S3) 12
6. Digestion UV–Vis spectroscopy 15
7. Calculation of pore filling and the probability p of multiple occupations 17
8. Synthesis of MOF-808, DUT-67 and MIP-206 and their composites (Scheme S4 and S5) 23
9. Scanning electron microscopy images of MOFs and composites 26
10. Photophysical properties of all composites 30
11. References 40

S1. General Information

Unless otherwise noted, all commercially available compounds were used as provided without further purification. Solvents used in reactions were p. A. grade. Solvents for ambient pressure column chromatography for the preparative purification of synthesis products were technical grade and distilled prior to use. Column chromatography was performed using silica gel Merck 60 (particle size 0.063 – 0.2 mm, Merck KGaA, Darmstadt, Germany). Solvent mixtures are understood as volume/volume. Analytical thin-layer chromatography (TLC) was performed on Macherey-Nagel silica gel aluminium plates (Macherey-Nagel GmbH & Co. Kg, Düren, Germany) with the F-254 indicator, and was visualized by irradiation with UV light (λ_{exc} = 254 or 360 nm). ^{1}H-NMR, ^{13}C-NMR, and ^{19}F-NMR were recorded on a Bruker Avance III 300 MHz NMR spectrometer in $CDCl_3$ (Bruker, Billerica, MA, USA). NMR data are reported as a chemical shift (δ) in ppm with multiplicities indicated as s (singlet), d (doublet), t (triplet), q (quartet), and m (multiplet). ^{1}H-NMR chemical shifts are referenced to the residual proton solvent signal versus TMS (δ($CHCl_3$ = 7.26). ^{13}C-NMR chemical shifts are referenced to the carbon solvent signal versus TMS (δ($CHCl_3$ = 77.2). ESI-MS was measured on a Bruker Daltonics UHR-QTOF maXis 4G (Bruker Daltonics GmbH & Co. KG, Bremen, Germany). All MS measurements were done on positive ion mode, and species can appear as protonated (m/z $[M+H]^+$), sodium adduct (m/z $[M+Na]^+$), or ammonium adduct (m/z $[M+NH_4]^+$). Powder X-ray diffraction (PXRD) analysis was conducted at an ambient temperature on a Rigaku Miniflex 600 powder diffractometer (Rigaku, Tokyo, Japan) using Cu Kα1 radiation with λ = 1.5406 Å (40 kV, 15 mA, 600 W) in the range of 2 θ = 2°– 50° and a flat silicon holder with low background and with a small indent for sample placement. N_2 sorption isotherms were obtained with a Belsorp MAXII high-precision gas/vapor adsorption measurement instrument (Microtrac MRB, Haan, Germany) or a Quantachrome Autosorb-6 at 77 K (Anton Paar QuantaTec, Boynton Beach, FL, USA). Scanning electron microscopy images were taken using a Jeol JSM-6510LV QSEM equipped with a LAB_6 cathode. The acceleration voltage was 20 kV. Before the measurement, the samples were coated with gold using a Jeol JFC 1200 Fine Coater (Jeol Ltd., Akishima, Tokyo, Japan). Optical measurements were carried out using a reflective setup on a FS5 photoluminescence spectrometer (Edinburgh Instruments, Livingston, UK) equipped with a 450 W Xe arc lamp. For this purpose, all solid samples were placed as solids in a brass sample holder. The quantum yield was measured with an integrating sphere (Ulbricht sphere) lined on the inside with BenFlect and exhibiting R > 99% between 350 nm and 2500 nm. All measurements were performed in ambient air.

S2. Sources of chemicals

Reagent	Manufacturer
2,4-Dimethyl-1H-pyrrole	BLDpharm
Benzaldehyde	ACROS Organics
Trifluoroacetic acid (TFA)	ACROS Organics
2,3-Dichloro-5,6-dicyano-1,4-benzoquinone (DDQ)	Sigma-Aldrich
Triethylamine	Fisher Chemical
Boron trifluoride etherate	TCI Chemicals
Magnesium sulfate	VWR Chemicals
Tin tetrachloride	ACROS Organics
Trimethylsilyl cyanide	Thermo Scientific
Sodium bicarbonate	Alfa Aesar
Zirconyl chloride octahydrate	Alfa Aesar
Zirconium(IV) chloride	Alfa Aesar
Dimethylformamide	Honeywell
Terephthalic acid	Alfa Aesar
Methanol	Sigma-Aldrich
Chloroform	Fisher Chemical
Dichloromethane	Sigma-Aldrich
2,5-Thiophenedicarboxylic acid	BLDpharm
Trimesic acid	BLDpharm
HCl (37%)	Sigma-Aldrich
Formic acid	Honeywell

S3. Synthesis of Bodipy compounds 1 and 2 (Scheme S1 and S2)

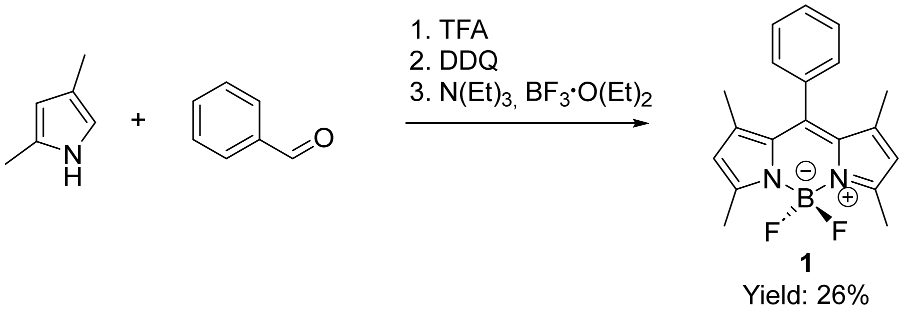

Scheme S1. Synthesis of 5,5-difluoro-1,3,7,9-tetramethyl-10-phenyl-Bodipy **1** according to Caruso et al. [1].

Under a nitrogen atmosphere, 1.13 mL of 2,4-dimethyl-1H-pyrrole (11 mmol) and 505.4 µL of benzaldehyde (5 mmol) were placed in dry dichloromethane (40 mL). Three drops of trifluoroacetic acid (TFA) were added to the solution. The reaction mixture was stirred for 4 h at room temperature. The progress of the reaction was monitored by TLC. After the benzaldehyde was completely consumed, 1.25 g of 2,3-dichloro-5,6-dicyano-1,4-benzoquinone (DDQ) (5.5 mmol) was added to the reaction, and the mixture was stirred for another 1.5 h. Subsequently, 4 mL of triethylamine and 4 mL of boron trifluoride diethyl etherate were added and stirred for 16 h. The mixture was washed three times with water (3 × 100 mL), and the organic layer was dried over $MgSO_4$. The solvent was removed under reduced pressure, and the crude product was purified by column chromatography on silica (cyclohexane/CH_2Cl_2 1:1) to afford 420 mg of difluoro-Bodipy **1** as a red solid.

Yield: 26%

^{1}H NMR (300 MHz, $CDCl_3$): δ = 7.49-7.47 (m, 3H), 7.30-7.26 (m, 2H), 5.98 (s, 2H), 2.56 (s, 6H), 1.37 (s, 6H) ppm (Figure S1).

^{19}F NMR (282 MHz, $CDCl_3$): δ = -146.31 (q, $^1J_{F\text{-}B}$ = 33.1 Hz) ppm (Figure S2).

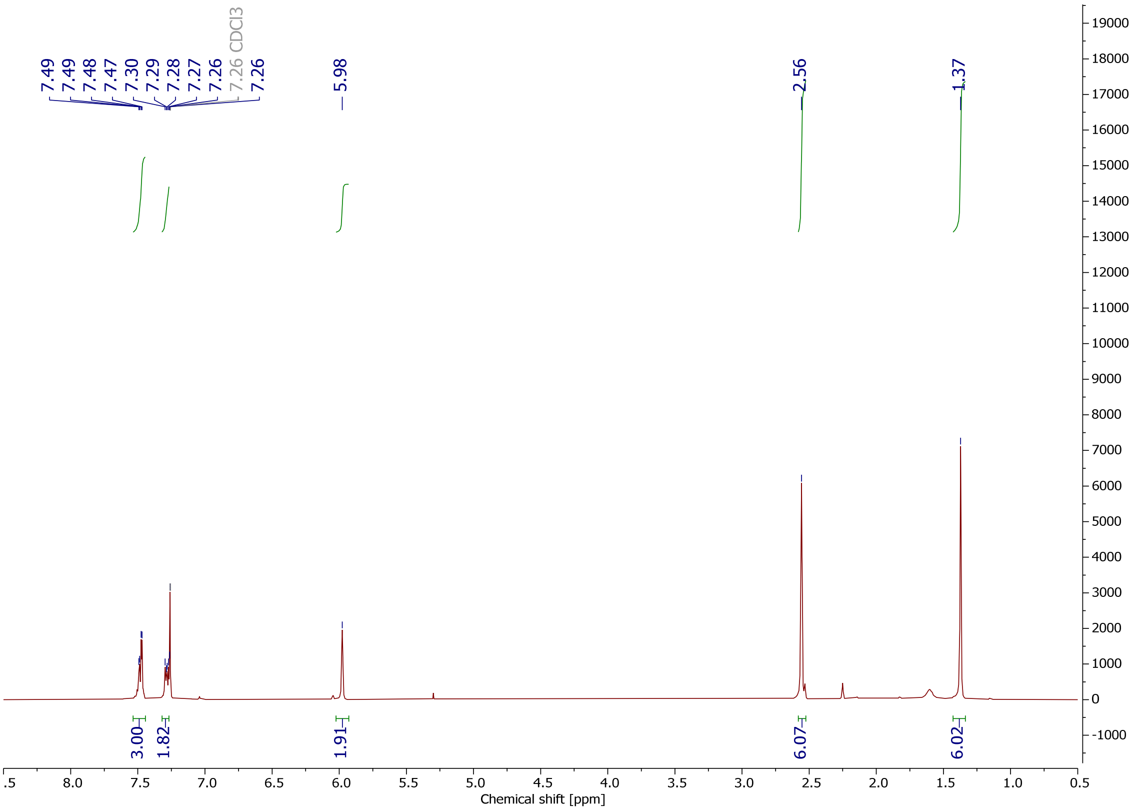

Figure S1. ^{1}H NMR spectrum (300 MHz) of difluoro-Bodipy **1** in $CDCl_3$.

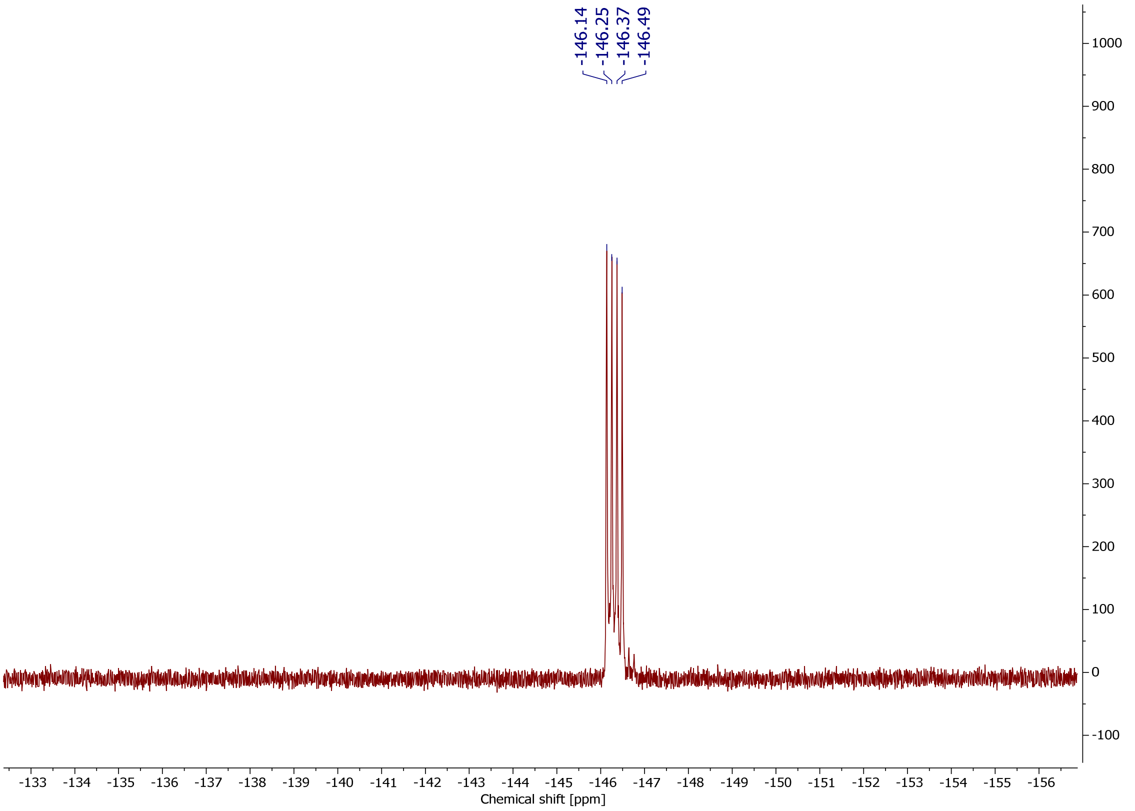

Figure S2. ^{19}F NMR (282 MHz) spectrum of difluoro-Bodipy **1** in $CDCl_3$.

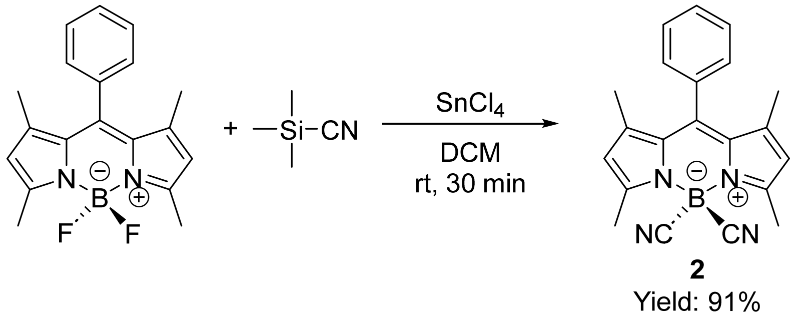

Scheme S2. Synthesis of 5,5-dicarbonitril-1,3,7,9-tetramethyl-10-phenyl-Bodipy **2** according to Nguyen et al. [2].

The amount of 200 mg of **1** (0.62 mmol) was dissolved in anhydrous dichloromethane (30 mL). Tin tetrachloride (36 µL, 0.31 mmol) in 5 mL of dichloromethane was added dropwise, followed by 388 µL of trimethylsilyl cyanide (3.1 mmol). After 30 min, the reaction was quenched with water and extracted three times with dichloromethane (3 × 50 mL). The organic phase was washed with a saturated aqueous $NaHCO_3$ solution (30 mL) and dried over $MgSO_4$. The solvent was removed under reduced pressure, and the crude product was purified by column chromatography on silica (cyclohexane/EtOAc 5:1) to afford 190 mg of dicyano-Bodipy **2** as a red to orange solid.

Yield: 91%

^{1}H NMR (300 MHz, $CDCl_3$): δ = 7.54-7.52 (m, 3H), 7.30-7.27 (m, 2H), 6.16 (s, 2H), 2.73 (s, 6H), 1.41 (s, 6H) ppm (Figure S3).

^{13}C NMR (75 MHz, $CDCl_3$): δ = 156.1, 144.6, 143.7, 134.1, 130.3, 129.8, 129.6, 127.8, 122.7, 122.5, 15.6, 14.7 ppm (Figure S4).

HRMS $[M+H]^+$ calculated ($^{12}C_{21}\,^{1}H_{20}\,^{11}B\,^{14}N_4$) 339.1776; found: 339.1784 (Figure S5).

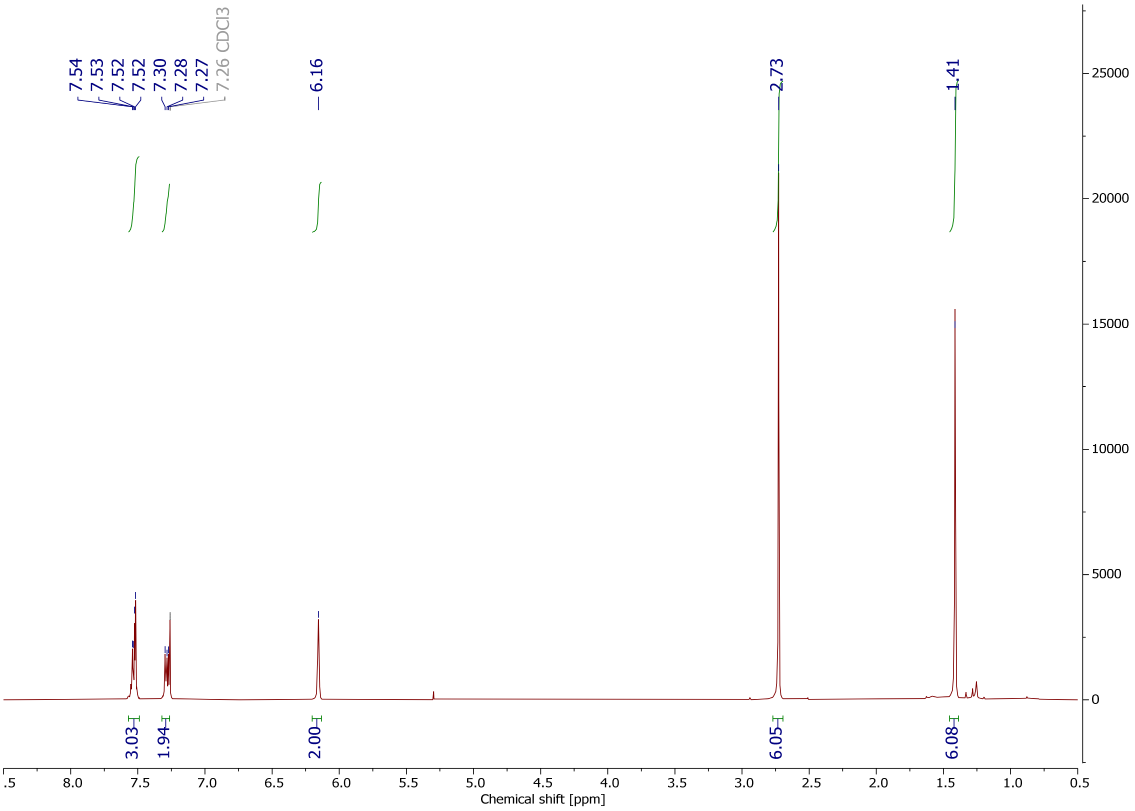

Figure S3. ^{1}H NMR (300 MHz) of dicyano-Bodipy **2** in $CDCl_3$.

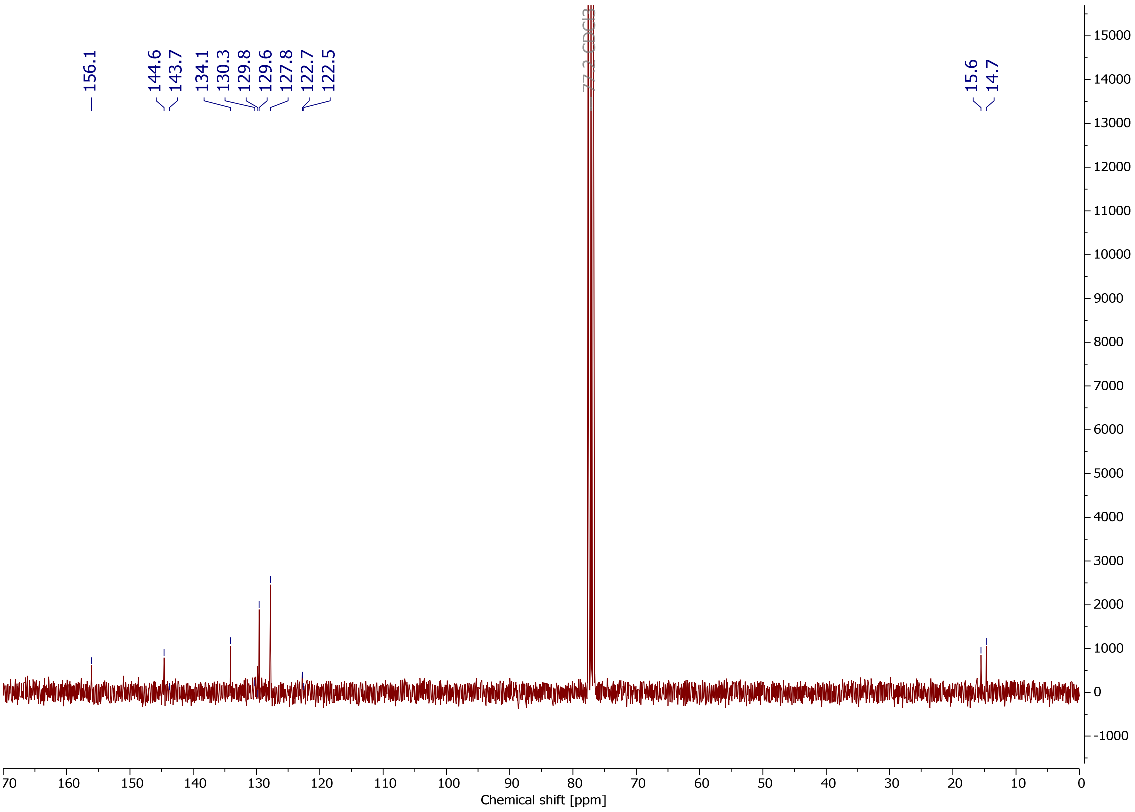

Figure S4. ^{13}C NMR (75 MHz) of dicyano-Bodipy **2** in $CDCl_3$.

Mass Spectrum SmartFormula Report

**Analysis Info**			
Analysis Name	D:\Data\Spektren 2024\JAN24HR000011.d	Acquisition Date	9/24/2024 10:19:56 AM
Method	tune_low_new.m	Operator	PT
Sample Name	Fetzer MF400 Fr.4-12 in CHCl3 (CH3OH)	Instrument	maXis 288882.20213
Comment			

Acquisition Parameter

Source Type	ESI	Ion Polarity	Positive	Set Nebulizer	0.3 Bar		
Focus	Not active	Set Capillary	4000 V	Set Dry Heater	180 °C		
Scan Begin	50 m/z	Set End Plate Offset	-500 V	Set Dry Gas	4.0 l/min		
Scan End	1500 m/z	Set Collision Cell RF	600.0 Vpp	Set Divert Valve	Source		

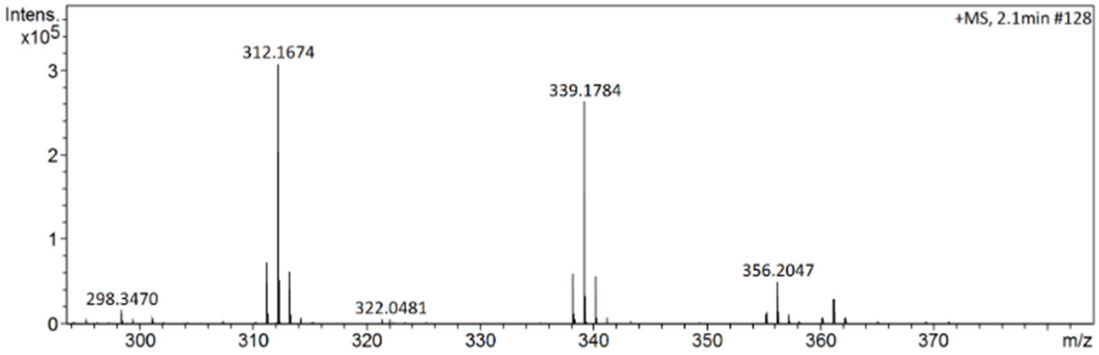

Meas. m/z	#	Ion Formula	m/z	err [ppm]	mSigma	# mSigma	Score	rdb	e⁻ Conf	N-Rule
312.1674	1	C20H19BN3	312.1667	-1.2	11.9	1	100.00	13.5	even	ok
339.1784	1	C21H20BN4	339.1776	-1.3	12.1	1	100.00	14.5	even	ok
356.2047	1	C20H27BNO4	356.2028	-4.4	6.7	1	48.38	8.5	even	ok
	2	C21H23BN5	356.2041	-0.6	7.9	2	100.00	13.5	even	ok
361.1601	1	C21H19BN4Na	361.1595	-0.5	8.6	1	100.00	14.5	even	ok
	2	C20H23BNaO4	361.1582	-4.3	14.8	2	42.42	9.5	even	ok

Figure S5. High-resolution ESI-MS of dicyano-Bodipy **2**.

S4. Photophysical properties of dicyano-Bodipy 2

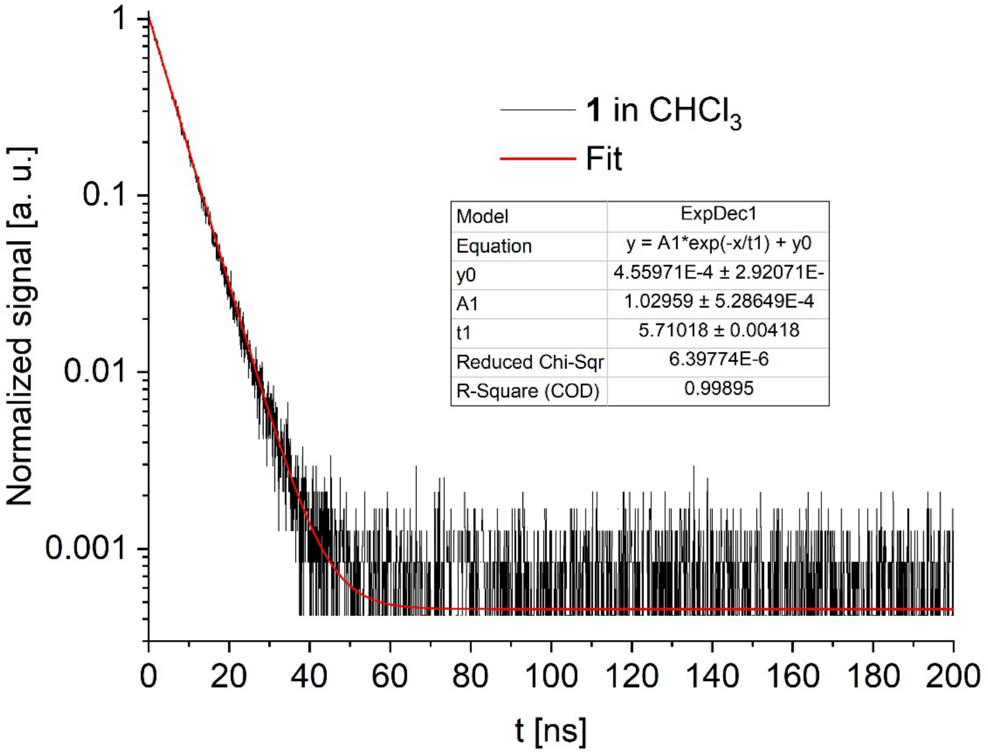

a)

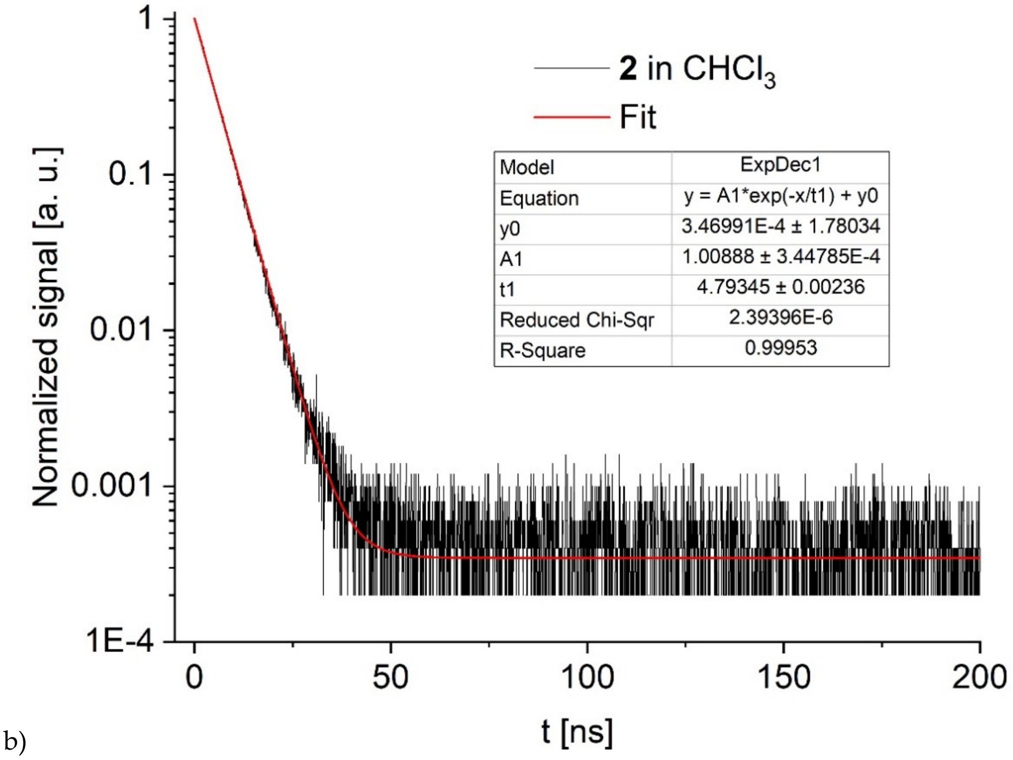

b)

Figure S6. Time-resolved photoluminescence decay (black) of the emission of a) **1** at 514.4 nm and b) **2** at 510 nm after excitation at 375 nm, both in chloroform solution (c = 0.5 mmol/L) with the respective exponential fitting parameters, including the pre-exponential factors A_1, lifetimes t_1, and confidence limits.

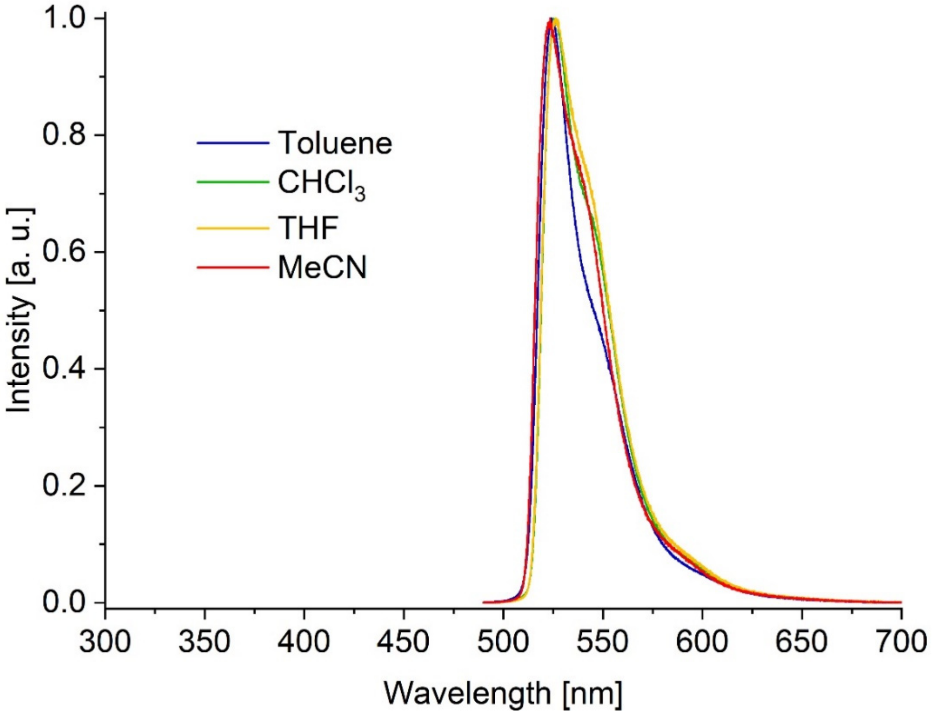

Figure S7. Emission spectra of **2** in different solvents. Excitation at 360 nm.

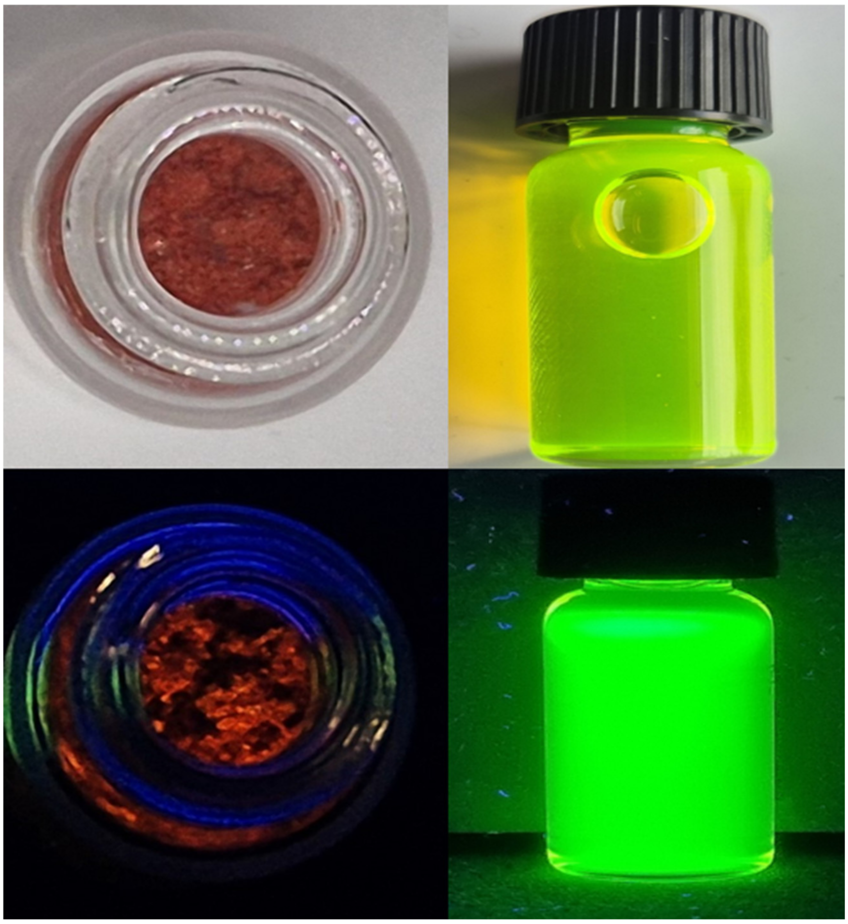

Figure S8. Neat dicyano-Bodipy **2** in the solid state (left) and dicyano-Bodipy **2** solution in $CHCl_3$ (8 mmol/L right) under daylight (top) and UV-light (λ_{exc} = 365 nm, bottom).

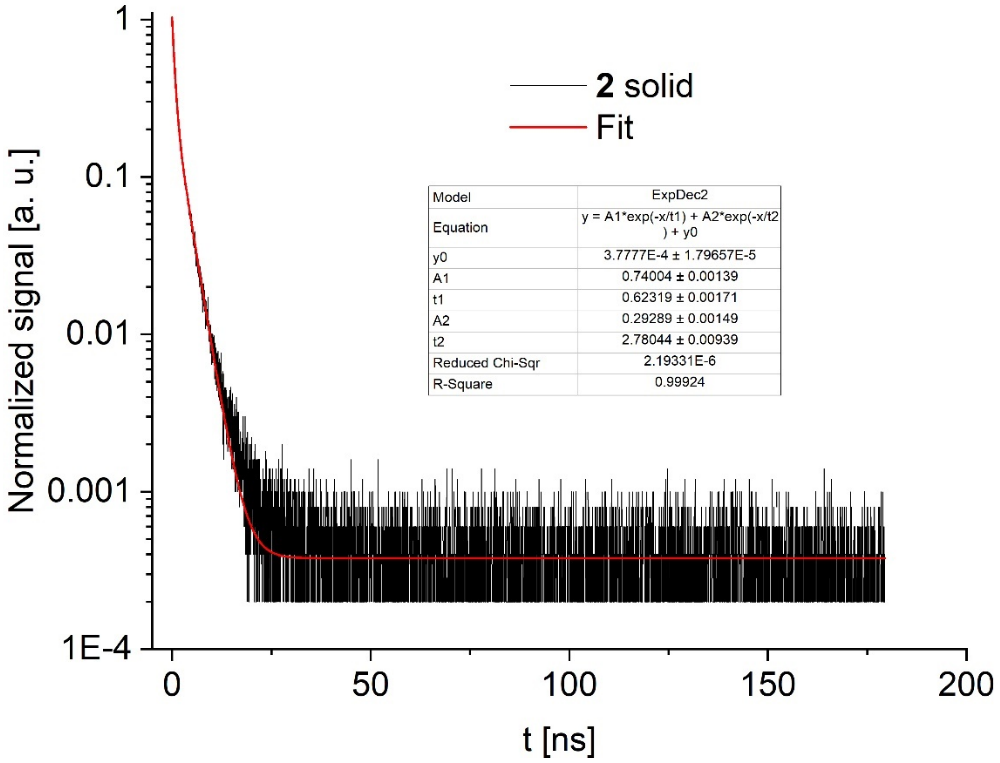

Figure S9. Time-resolved photoluminescence decay (black) of the emission of neat **2** in the solid state at 594 nm after excitation at 375 nm with the respective two-exponential fitting parameters, including the pre-exponential factors A_1 and A_2, lifetimes t_1 and t_2, and confidence limits.

S5. Synthesis of UiO-66 and 2@UiO-66 composites (Scheme S3)

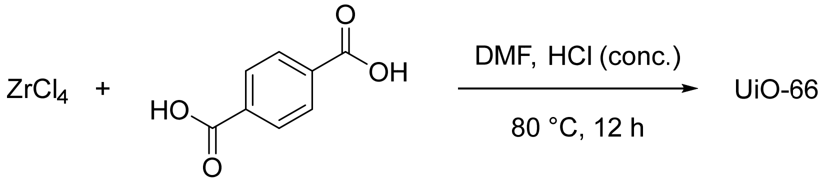

Scheme S3. Synthesis of UiO-66 according to the synthesis of Katz et al. with slight modifications [3].

A 16 mL Pyrex tube was loaded with 125 mg of $ZrCl_4$, 125 mg of H_2BDC, 1 mL of concentrated HCl (37 %), and 15 mL of DMF. The reaction mixture was sonicated for 20 minutes until all solids were dissolved. The reaction mixture was then heated to 80 °C for 12 hours. The white solid obtained was filtered off and washed with DMF (3 × 30 mL) and MeOH (3 × 30 mL). The sample was dried in a vacuum oven at 60 °C for 24 hours and then activated under high vacuum at 150 °C for 16 hours.

The post-synthetic wet infiltration was carried out with stirring for a period of 5 days in a 6 mL solution with a concentration of 1.0 mmol/L of **2** in dichloromethane with 80 mg of activated UiO-66, which led to the composite **2**@UiO-$66_{0.44}$. For the in situ synthesis of the composites, the above-described synthesis protocol was used. Thereby, a defined part of the 15 mL of neat DMF was replaced by a DMF solution of **2** with a concentration of 15 mmol/L (see Table S1), resulting in concentrations of **2** in the reaction mixtures of 3, 7, 10, and 15 mmol/L. The reactions, depending on the increasing concentration, led to the composites **2**@UiO-$66_{1.1}$, **2**@UiO-$66_{2.3}$, **2**@UiO-$66_{4.7}$, and **2**@UiO-$66_{7.2}$.

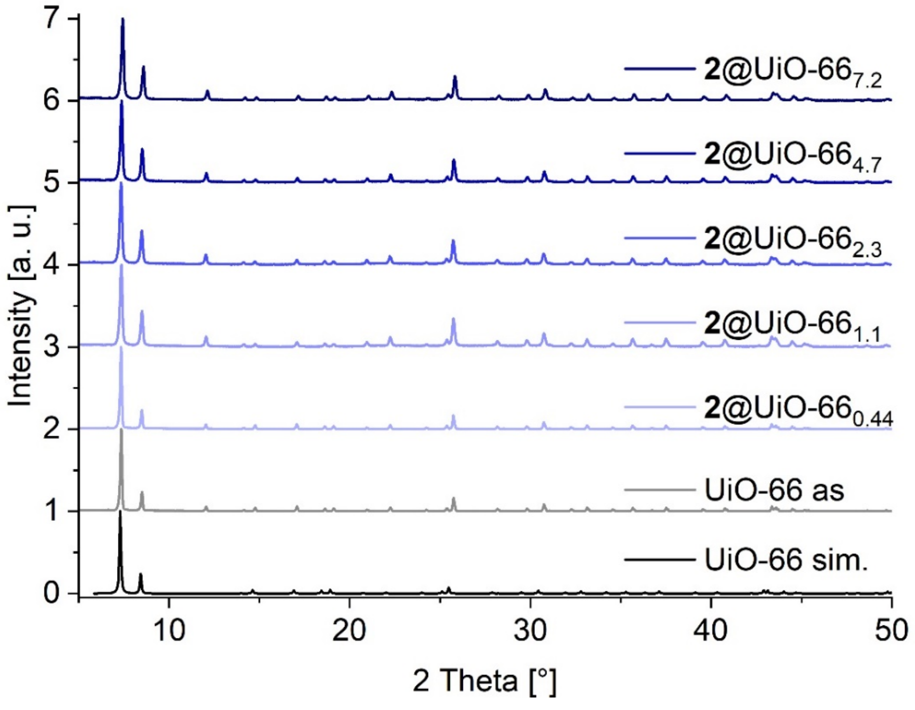

Figure S10. PXRD of UiO-66 and all UiO-66 composites. Simulation of UiO-66 from CCDC No. 752051 [4].

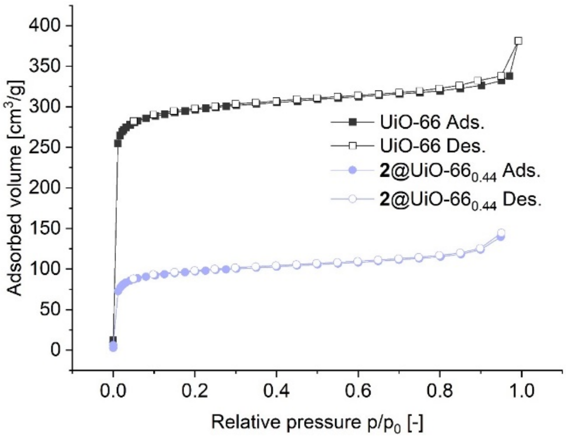

Figure S11. Nitrogen sorption isotherms at 77 K of UiO-66 and the post-synthetic composite with SBET = 1196 m²/g and 381 m²/g, respectively (filled symbols: adsorption; empty symbols: desorption).

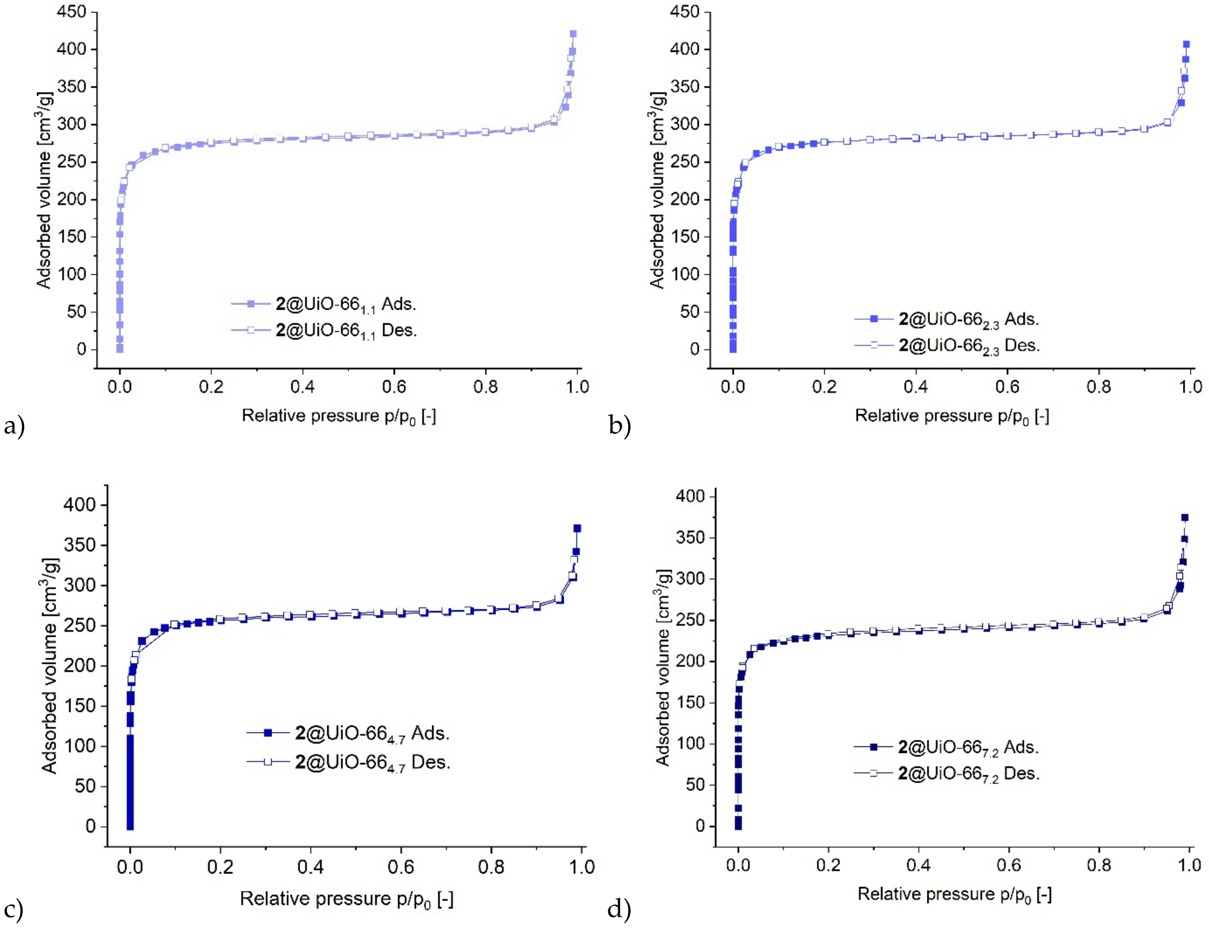

Figure S12. Nitrogen sorption isotherms at 77 K of in situ synthesized **2**@UiO-66 composites with SBET = 1115 m²/g (a), 1096 m²/g (b), 1022 m²/g (c), and 916 m²/g (d) (filled symbols: adsorption; empty symbols: desorption).

Table S1. Concentrations and BET surface area determinations of the in situ prepared composites.

Compound	Volume of DMF solution of 2 [a] [mL]	Concentrations of 2 in the reaction mixtures [mmol/L]	S_{BET} [m²/g]
**UiO-66 synthesized**	-	-	1192
**a) 2@UiO-66$_{1.1}$**	3	3	1115
**b) 2@UiO-66$_{2.3}$**	7	7	1096
**c) 2@UiO-66$_{4.7}$**	10	10	1022
**d) 2@UiO-66$_{7.2}$**	15	15	916

[a] DMF solution of **2** with a concentration of 15 mmol/L.

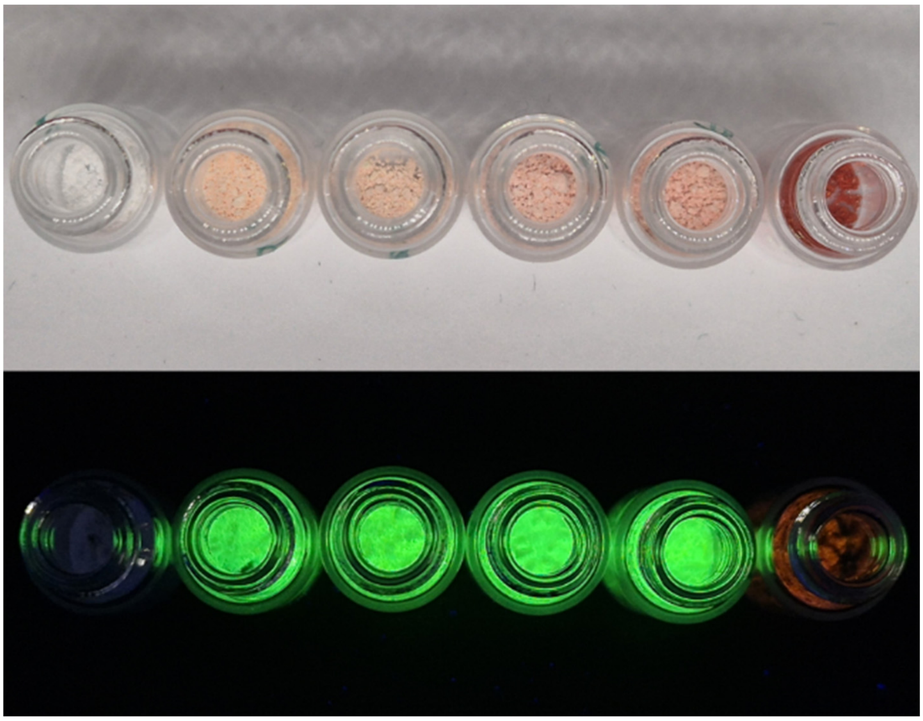

Figure S13. From left to right, neat UiO-66, the four in situ prepared UiO-66 composites **2**@UiO-66$_{1.1}$, **2**@UiO-66$_{2.3}$, **2**@UiO-66$_{4.7}$, **2**@UiO-66$_{7.2}$, and neat **2** as a solid under daylight (top) and UV-light (λ_{exc} = 365 nm, bottom).

S6. Digestion UV–Vis spectroscopy

Carefully weighted aliquots (see example below and Table S2) of all post-synthetic and in situ samples of **2**@MOF were digested under strong acidic (conc. HCl for DUT-67) or basic (1 mol/L KOH for UiO-66, MOF-808, and MIP-206) conditions in order to determine the loading of **2** in the MOF. Dicyano-Bodipy **2** was extracted from the aqueous phase with dichloromethane, the solvent was removed under reduced pressure using a rotary evaporator, the solid residue was dried under high vacuum, and it was then dissolved in a defined amount of 3.0 mL of chloroform for the post-synthetic composites and 5.0 mL of chloroform for the in situ composites. The amount of **2** in each MOF could be determined using the previously generated calibration curve (Figure S14) [5-8].

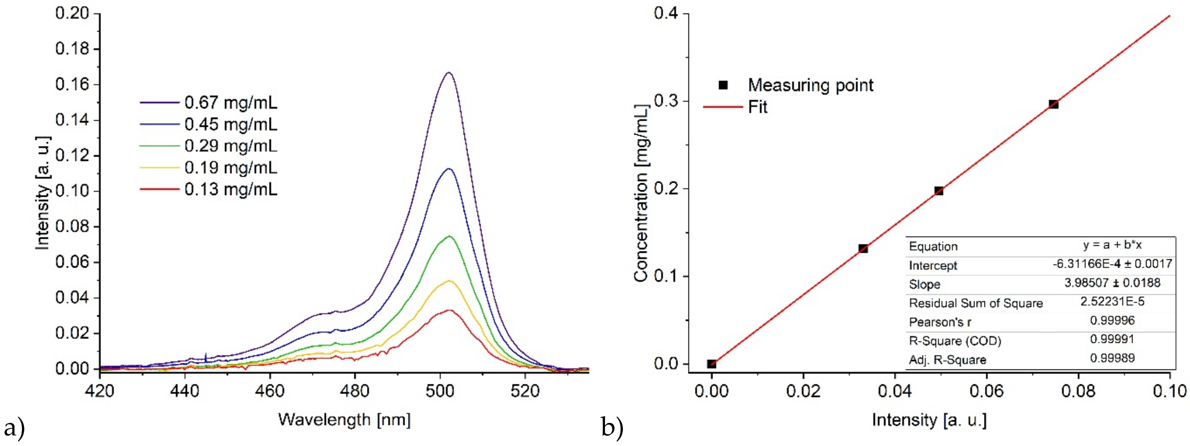

Figure S14. a) UV–Vis spectra of different concentrated solutions of **2** in chloroform. b) Calibration line with fit and R-values. Values were taken at a wavelength of 502 nm.

The mass of Bodipy **2** in the analytical sample (weight of incorporated Bodipy) is obtained by multiplying the absorption intensity by the slope of the calibration curve and the volume of chloroform. The loading of Bodipy in MOFs can then be calculated using the following equation (1):

$$loading\ (wt\%) = \frac{weight\ of\ incorporated\ \boldsymbol{Bodipy}}{weight\ of\ \boldsymbol{Bodipy}@MOF} \times 100 \quad (1)$$

For example, for post-synthetic **2**@UiO-66$_{0.44}$ (eq. (2)), 16.3 mg of the composite was digested and the extracted Bodipy dissolved in 3.0 mL of chloroform, resulting in a rounded intensity of 0.006 at λ_{em} = 502 nm.

$$loading\ (0.44\ wt\%) = \frac{0.006 \times 3.985 \times 3}{16.3} \times 100 \quad (2)$$

For example, for in situ **2**@UiO-66$_{4.7}$ (eq. (3)), 16.1 mg of the composite was digested, and the extracted Bodipy was dissolved in 5.0 mL of chloroform, resulting in a rounded intensity of 0.038 at λ_{em} = 502 nm.

$$loading\ (4.7\ wt\%) = \frac{0.038 \times 3.985 \times 5}{16.1} \times 100 \quad (3)$$

Table S2. Determination of the Bodipy **2** loading in the in situ prepared UiO-66 composites using the UV–Vis digestion method. All in situ prepared UiO-66 composites were dissolved in 5 mL of chloroform.

**Bodipy 2 concentration in synthesis [mmol/L]**	**Mass of composite digested [mg]**	**Intensity at λ_{em} = 502 nm**	**Concentration from UV–Vis calibration curve [mg/mL]**	**Loading [wt%]**	**Sample name**
0	14.2	0	0	0	UiO-66
3	14.8	0.008	0.03	1.1	**2**@UiO-66$_{1.1}$
7	14.4	0.016	0.07	2.3	**2**@UiO-66$_{2.3}$
10	16.1	0.038	0.15	4.7	**2**@UiO-66$_{4.7}$
15	15.8	0.057	0.23	7.2	**2**@UiO-66$_{7.2}$

Table S3. Determination of the Bodipy **2** loading of individual composites for MOF-808, DUT-67, and MIP-206 using the UV–Vis method described.

**MOF**	**Mass of composite digested [mg]**	**Intensity at λ_{em} = 502 nm**	**Concentration from UV–Vis calibration curve [mg/mL]**	**Loading [wt%]**
**2@MOF-808**$_{0.57}$[a]	16.9	0.008	0.03	0.57
**2@MOF-808**$_{1.9}$[b]	16.2	0.016	0.06	1.9
**2@DUT-67**$_{0.51}$[a]	16.3	0.007	0.03	0.51
**2@DUT-67**$_{2.2}$[b]	12.4	0.018	0.07	2.2
**2@MIP-206**$_{0.3}$[a]	17.4	0.004	0.02	0.30

[a] Extract after digestion was dissolved in 3.0 mL of chloroform. [b] Extract after digestion was dissolved in 5.0 mL of chloroform.

S7 Calculation of pore filling and the probability p of multiple occupations

We used the crystal structures of the respective MOFs to visualize the pores. It is crucial that only pores capable of accommodating the dye molecule are considered.

Structure of UiO-66:

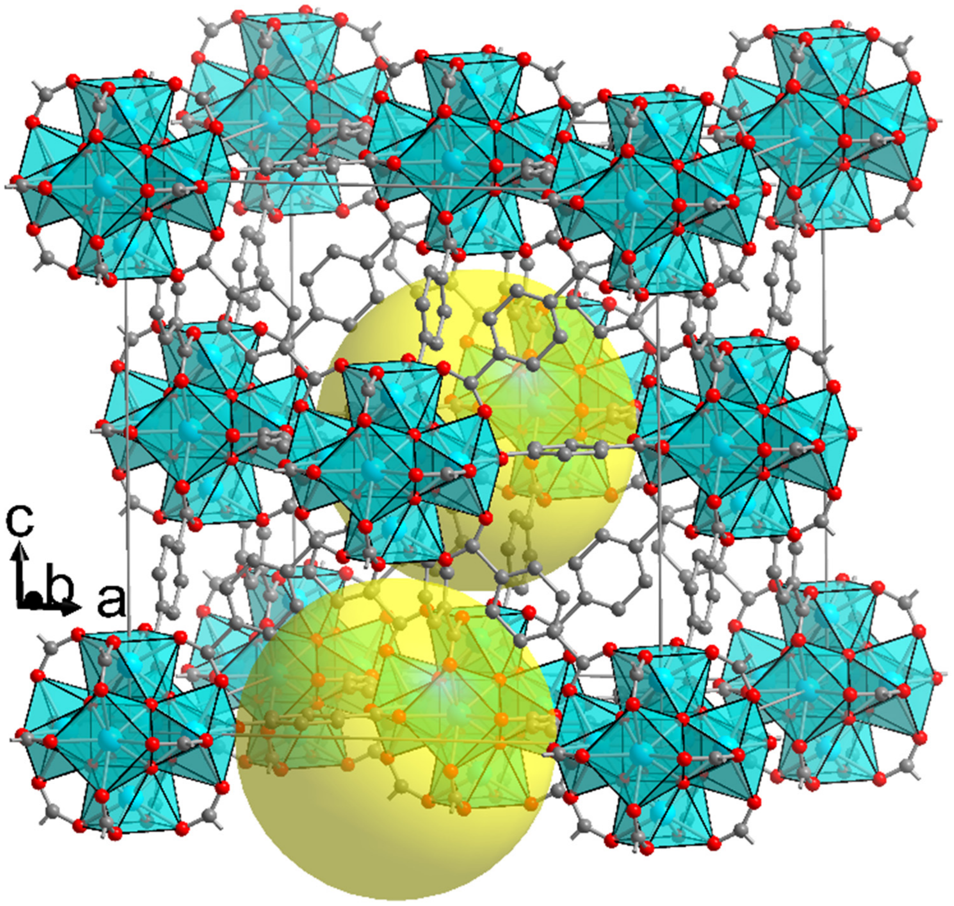

Figure S15. Structure of zirconium terephthalate UiO-66, with the formula unit 3D-$[Zr_6O_4(OH)_4(BDC)_6]$ in the face-centered cubic **fcu** structure, showing the octahedral pore in yellow in the center and on one of the edges. The diameter of the yellow sphere is 12 Å. The UiO-66 structure was drawn from the deposited CIF files under CCDC 837796 [9]. Hydrogen atoms are omitted for clarity.

UiO-66 contains two main types of pores: tetrahedral pores with diameters of approximately 8 Å (0.8 nm) and octahedral pores with a diameter of 11 Å (1.1 nm). Only the latter is capable of absorbing Bodipy. There are four formula units per unit cell. Two of the octahedral pores are indicated as yellow spheres. There is one octahedral pore in the center of the cubic unit cell and one on each of the 12 edges of the cube, counting as ¼, giving an additional three octahedral pores or four in total per unit cell. Thus, there is one octahedral pore per formula unit of $[Zr_6O_4(OH)_4(BDC)_6]$ (1664.01 g/mol).

Based on the number of pores per formula unit (1:1), the formula unit of **2** (338.22 g/mol), and the UV–Vis analysis for **2**@UiO-66 from Table S2, we can calculate the ratio of **2** to octahedral pores (eq. (4)). This will be illustrated using the example **2**@UiO-66$_{7.2}$.

2@UiO-66$_{7.2}$ contains 72 mg of **2** per gram of composite, which then contains 1–0.072 = 0.928 mg of UiO-66. This corresponds to 0.21 mmol of **2** and 0.56 mmol of UiO-66, which equals 0.56 mmol of pores because of the 1:1 ratio (eq. (5)).

$$n\left(\frac{Bodipy}{pore}\right) = filled\ pores \tag{4}$$

$$n\left(\frac{Bodipy}{pore}\right) = \frac{0.21\ mmol}{0.56\ mmol} = 0.375 \tag{5}$$

The result is a pore filling of 37.5% of the existing octahedral pores, leaving 62.5% of the pores empty. Based on this result, we were able to calculate the probability p of multiple occupancy. For this, we used a Poisson distribution (eq. (6) and (7)).

$$p(n) = \frac{\alpha^n \times e^{-\alpha}}{n!} \tag{6}$$

$$\alpha = -\ln(unoccupied\ pores) \tag{7}$$

In the case of **2**@UiO-66$_{7.2}$ $\alpha = -\ln\,(0.625) = 0.47$

Table S4. Calculations of the probability p of multiple occupancy for **2**@UiO-66$_{7.2}$.

**2@UiO-66$_{7.2}$**	**Calculated probability**	**Probability in %**
**p(one)**	0.293	78.1
**p(two)**	0.069	18.4
**p(≥three)**	0.013	3.5
**Sum**	0.375	100

The sum of all probabilities must equal 1 (when adding p(zero) = 0.625). Additionally, the expected value of α should equal the sum of all probabilities p multiplied by the number of occupations n (eq. (8)).

$$\alpha = \sum p(n) \times n \tag{8}$$

Thus, from the above calculated probabilities. $\alpha = 0.47 = 0.293 \times 1 + 0.069 \times 2 + 0.013 \times 3$

Structure of MOF-808:

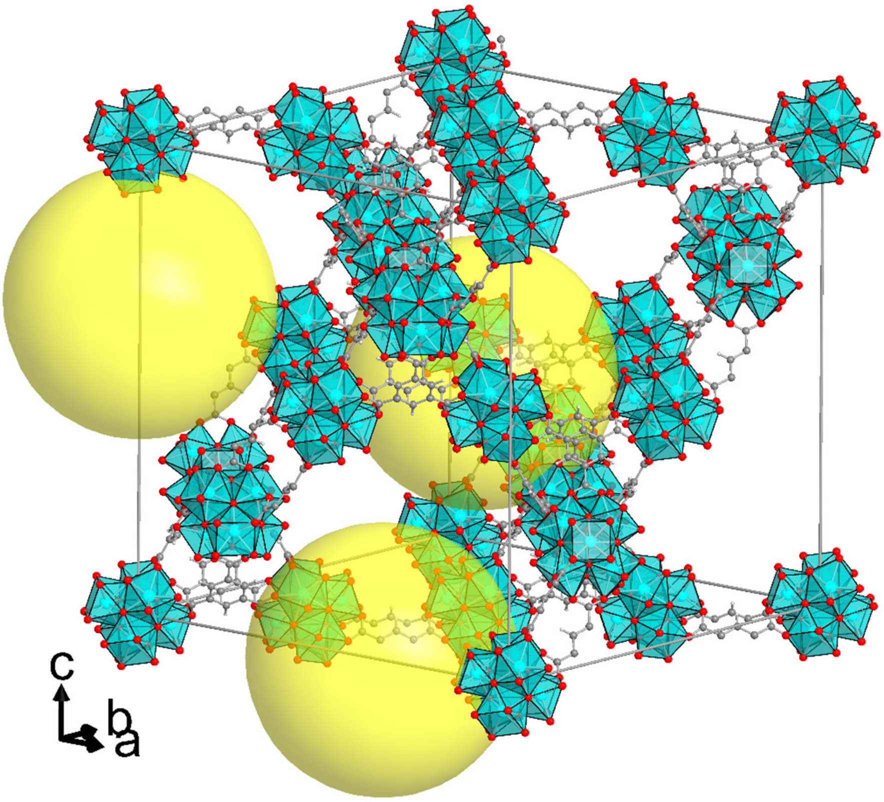

Figure S16. Structure of zirconium benzene-1,3,5-tricarboxylate MOF-808, with the formula unit 3D-[$Zr_6O_4(OH)_{10}(BTC)_2(H_2O)_6$] and an expanded diamond (dia-a) net, consisting of 6-connected Zr-based clusters (SBUs) and trimesate linkers. Its structure features two types of interconnected voids: small, isolated tetrahedral cages (not highlighted) and large, adamantane-shaped pores. The latter in yellow are shown in the center, and two exemplarily on two of the edges (at 0,0,0.66 and at 0.66,0,0). The diameter of the yellow sphere is 20 Å. The MOF-808 structure was drawn from the deposited CIF file under CCDC 1002627 [10]. Note that in this CIF file, there are bridging and terminal formiate linkers at the Zr_6 clusters, giving a formula unit of -[$Zr_6O_4(OH)_4(BTC)_2(HCOO)_6$] [10]. We followed the synthesis of Reinsch et al. [11], who did not provide a CIF file but had given a formula unit of [$Zr_6O_4(OH)_{10}(BTC)_2(H_2O)_6$], and we used this formula unit in our subsequent calculations here.

MOF-808 contains two main types of pores: large, interconnected hexagonal channels with diameters of approximately 18 Å (1.8 nm), and isolated tetrahedral cages with internal pore diameters around 4.8 Å (or 0.48 nm). Thus, this hierarchical pore structure of MOF-808 consists of both micropores (tetrahedral cages) and mesopores (hexagonal channels). Only the latter are accessible for Bodipy **2**.

There are 16 formula units per unit cell. There is one pore in the center of the cubic unit cell and one on each of the 12 edges of the cube, counting as ¼, giving an additional three pores or four in total per unit cell. Thus, there is ¼ = 0.25 pores per formula unit of [$Zr_6O_4(OH)_{10}(BTC)_2(H_2O)_6$] (1303.7 g/mol).

Using the number of pores per formula unit (0.25:1), the formula unit of **2** (338.22 g/mol), and the digestion UV–Vis analysis for the in situ **2**@MOF-808 from Table S3, we can calculate the ratio of **2** to the pores. This will be illustrated using the example **2**@MOF-808$_{1.9}$.

2@MOF-808$_{1.9}$ contains 19 mg of **2** per gram of composite, which then contains 1–0.019 = 0.981 mg of MOF-808. This corresponds to 0.056 mmol of **2** and 0.75 mmol of MOF-808, which equals 0.25 × 0.75 mmol of pores because of the 0.25:1 ratio.

$$n\left(\frac{Bodipy}{pore}\right) = \frac{0.056\,mmol}{0.188\,mmol} = 0.298 \quad (9)$$

The result is a pore filling of 29.8% of the existing pores (eq. (9)), leaving 70.2% of the pores empty. Based on this result, we calculated the probability p of multiple occupancy using the above Poisson distribution.

In the case of **2**@MOF-808$_{1.9}$ $\alpha = -\ln\,(0.702) = 0.354$

The results of the calculation are listed in Table S5.

The sum of all probabilities must equal 1 (when adding p(zero) = 0.702). Additionally, the expected value of α should equal the sum of all probabilities p multiplied by the number of occupations n (eq. (10)).

$$\alpha = \sum p(n) \times n \quad (10)$$

Thus, from the subsequent calculated probabilities. $\alpha = 0.354 = 0.2484 \times 1 + 0.044 \times 2 + 0.0058 \times 3$

Structure of DUT-67:

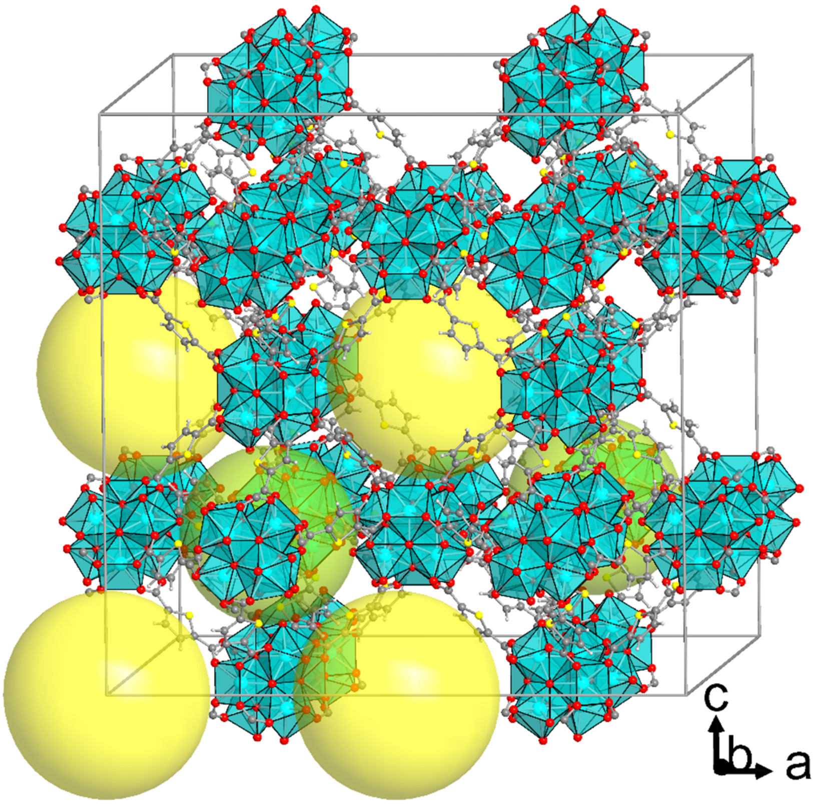

Figure S17. Structure of zirconium thiophenedicarboxylate DUT-67, with the formula unit 3D-$[Zr_6O_4(OH)_8(TDC)_4(H_2O)_6]$ in the reo topology, featuring cuboctahedral cages (yellow) and octahedral cages (light green). The cuboctahedral cages in yellow (sphere diameter 14 Å) are shown in the center, and one each exemplarily on one of the face centers, edge centers, and vertices. Two octahedral cages in light green (sphere diameter 12 Å) are exemplarily given at 0.25,0.25,0.25 and at 0.75,0.75,0.25. The DUT-67 structure was drawn from the deposited CIF file under CCDC 921644 [12].

DUT-67 has a hierarchical porous structure containing two types of pores: A cuboctahedral pore with a diameter of 14.2 Å (1.42 nm), and an octahedral pore with a diameter of 11.7 Å (1.17 nm). Both of which can encapsulate Bodipy, and we took both pores into account when calculating the occupation.

In DUT-67, there are 24 formula units of $[Zr_6O_4(OH)_8(TDC)_4(H_2O)_6]$ (1536.1 g/mol)per unit cell. There is one cuboctahedral pore in the center of the cubic unit cell, one on each center of the six faces, counting ½, one on the midpoint of each of the 12 edges of the cube, counting as ¼, and one on each of the eight vertices, counting 1/8, giving in total eight cuboctahedral pores per unit cell. Further, there are eight octahedral pores per unit cell. Combined, this gives 16 accessible pores per unit cell with its 24 formula units. We expect an even distribution across both types of pores. This results in a ratio of 0.66 pores per formula unit of DUT-67.

Using the number of pores per formula unit (0.66:1), the formula unit of **2** (338.22 g/mol), and the digestion UV–Vis analysis for the in situ **2**@DUT-67 from Table S3, we can calculate the ratio of **2** to the pores. This will be illustrated using the example **2**@DUT-67$_{2.2}$.

2@DUT-67$_{2.2}$ contains 22 mg of **2** per gram of composite, which then contains 1–0.022 = 0.978 mg of DUT-67. This corresponds to 0.065 mmol of **2** and 0.637 mmol of DUT-67, which equals 0.66 × 0.637 mmol of pores because of the 0.66:1 ratio.

$$n\left(\frac{Bodipy}{pore}\right) = \frac{0.065\ mmol}{0.42\ mmol} = 0.155 \tag{11}$$

The result is a pore filling of 15.5% of the existing cuboctahedral and octahedral pores (eq. (11)), leaving 84.5% of the pores empty. Based on this result, we calculated the probability p of multiple occupancy using the above Poisson distribution.

In the case of **2**@DUT-67$_{2.2}$ $\alpha = -\ln(0.845) = 0.168$

The results of the calculation are listed in Table S5.

The sum of all probabilities must equal 1 (when adding p(zero) = 0.845). Additionally, the expected value of α should equal the sum of all probabilities p multiplied by the number of occupations n (eq. (12)).

$$\alpha = \sum p(n) \times n \tag{12}$$

Thus, from the subsequent calculated probabilities. $\alpha = 0.168 = 0.1421 \times 1 + 0.012 \times 2 + 0.0007 \times 3$

Table S5. Calculations of the probability p of multiple occupancy for **2**@MOF-808$_{1.9}$ and **2**@DUT$_{2.2}$.

	2@MOF-808$_{1.9}$		2@DUT-67$_{2.2}$	
**n_{av}(Bodipy/pore)**	0.298		0.155	
	Calc. probability	Probability in %	Calc. probability	Probability in %
**p (one)**	0.2484	83.4	0.1421	91.7
**p (two)**	0.044	14.7	0.012	7.7
**p (≥three)**	0.0058	1.9	0.0007	0.6
**Sum**	0.298	100	0.155	100

S8. Synthesis of MOF-808, DUT-67, and MIP-206 and their composites (Scheme S4 and S5)

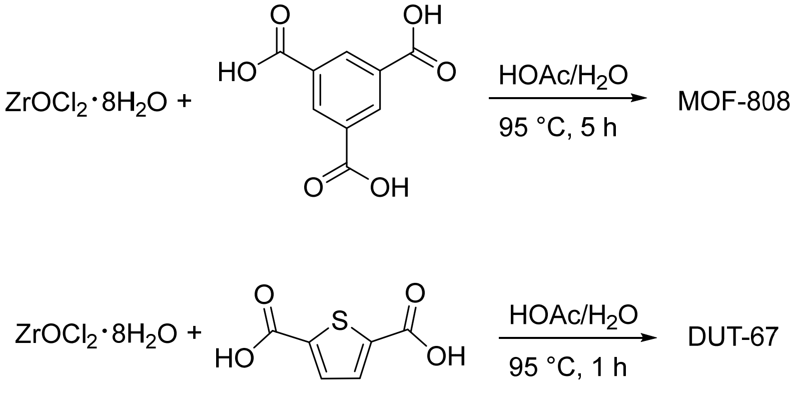

Scheme S4. Synthesis of MOF-808 and DUT-67 in a microwave oven according to a procedure by Reinsch et al. [11].

For MOF-808, 280 mg (1.33 mmol) of 1,3,5-benzenetricarboxylic acid, and for DUT-67, 458 mg (2.66 mmol) of 2,5-thiophenedicarboxylic acid were heated together with 1.288 g (4 mmol) of $ZrOCl_2 \cdot 8H_2O$ in a mixture of 10 mL of water and 10 mL of acetic acid while stirring to 95 °C. The reaction time for MOF-808 was set to 5 h. The reaction time for DUT-67 was set to 1 h. Both MOFs were then separated by centrifugation and washed twice with 15 mL of aqueous sodium acetate solution and once with 15 mL of ultrapure water. The centrifuged products were dried in a vacuum oven at 60 °C for 24 hours and then activated under high vacuum at 150 °C for 16 hours. The respective in situ composites with **2** were prepared in the same way with a concentration of 6 mmol/L of **2** in acetic acid.

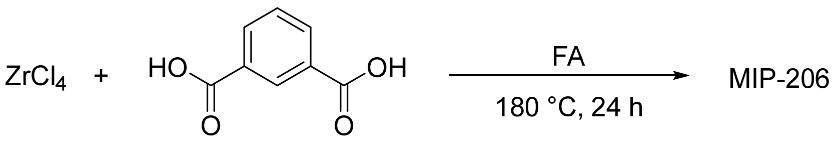

Scheme S5. Synthesis of MIP-206 according to the literature by Wang et al. [13].

MIP-206 was synthesized according to the literature by Wang et al. [13]. 1.1 g (6.6 mmol) of isophthalic acid was added to a Teflon reactor together with 5 mL of formic acid and stirred for 5 minutes. Then 2.0 g of $ZrCl_4$ (8.6 mmol) was added, and the mixture was stirred again for 10 minutes. The reactor was then sealed and heated to 180 °C over a period of two hours. This temperature was maintained for 24 h. The reaction was allowed to cool under ambient conditions to room temperature. The resulting white solid was separated by centrifugation and was washed three times with 20 mL of acetone each and dried in air. It was activated under high vacuum at 150 °C for 16 hours.

Only pores larger than 10 Å can be considered for the incorporation of **2** into the respective MOFs. MOF-808 contains only one type of pore larger than 10 Å (18.4 Å for the adamantane pore; the tetrahedral pore has a size of 4.8 Å), while DUT-67 features two types of pores, both with diameters exceeding 10 Å (14.2 Å for cuboctahedral and 11.7 Å for octahedral pores). Compared to all the other MOFs, MIP-206 has a channel structure with a diameter of 26 Å.

The post-synthetic wet infiltrations were carried out with stirring 80 mg of activated MOF for a period of 5 days in a 6 mL solution of **2** in dichloromethane with a concentration of 1.0 mmol/L, which led to the composites **2**@MOF-808$_{0.57}$, **2**@DUT-67$_{0.51}$, and **2**@MIP-206$_{0.3}$.

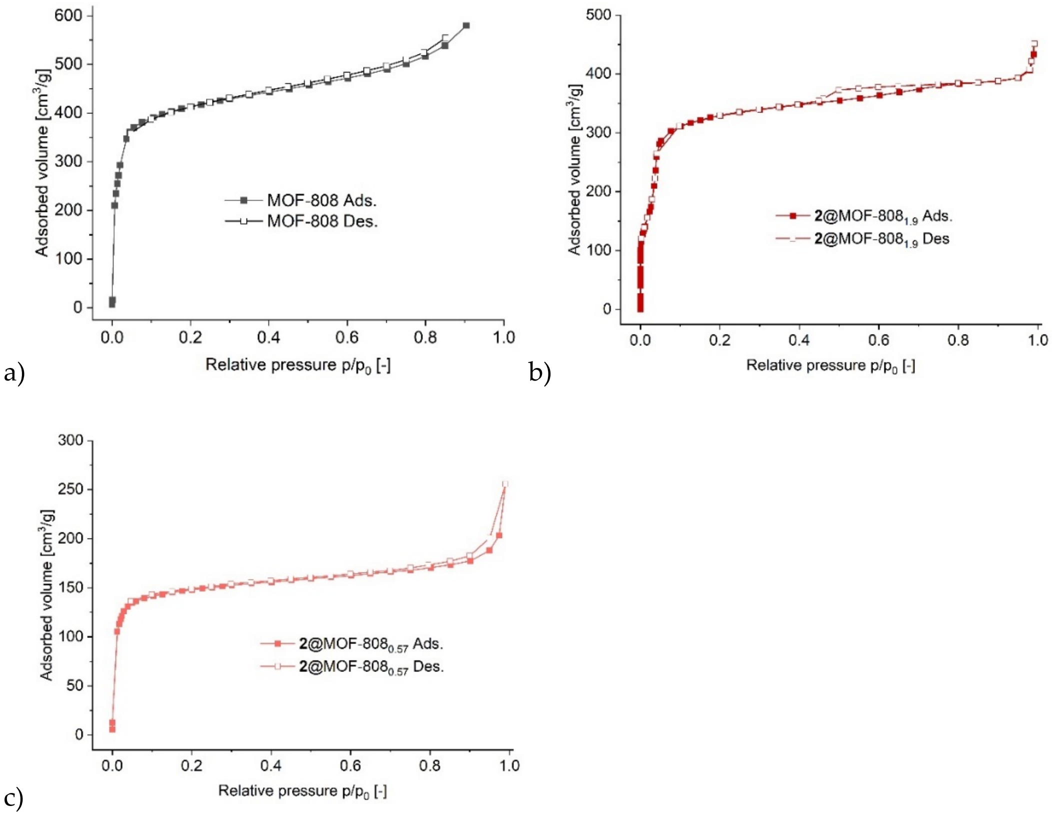

Figure S18. Nitrogen sorption isotherms at 77 K of a) neat MOF-808, b) **2**@MOF-808$_{1.9}$, and c) **2**@MOF-808$_{0.57}$ (filled symbols: adsorption; empty symbols: desorption).

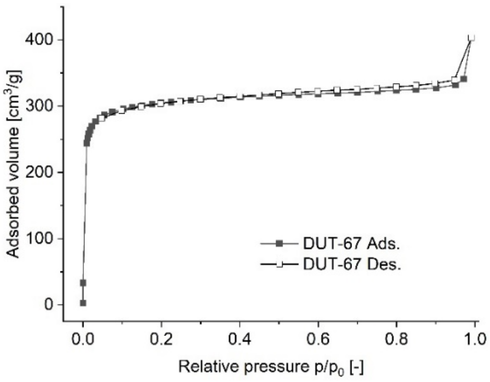

a)

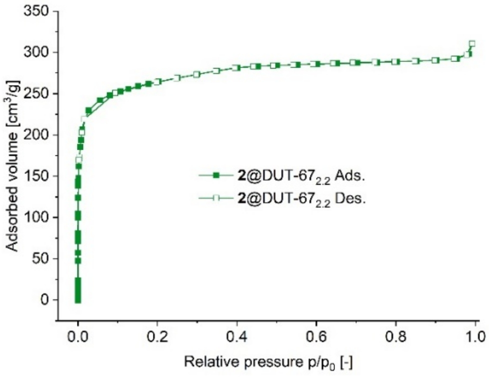

b)

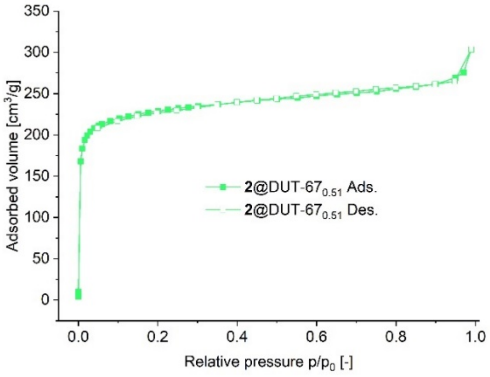

c)

Figure S19. Nitrogen sorption isotherms at 77 K of a) neat DUT-67, b) **2**@DUT-67$_{2.2}$, and c) **2**@DUT-67$_{0.51}$ (filled symbols: adsorption; empty symbols: desorption).

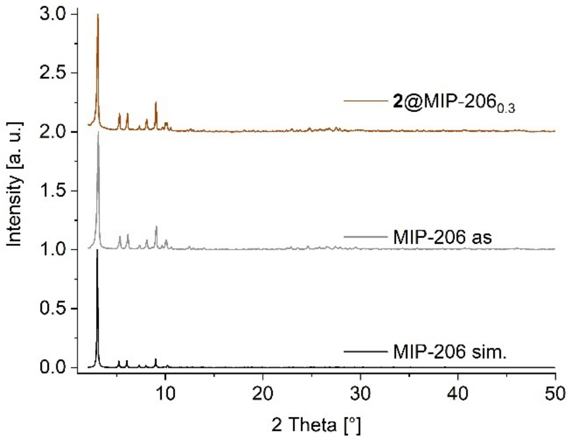

a)

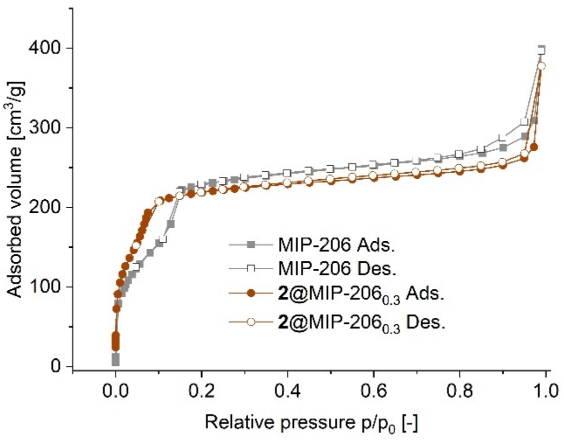

b)

Figure S20. a) PXRD of MIP-206 and post-synthetic **2**@MIP-206$_{0.3}$ composite. Simulation of MIP-206 from CCDC No. 2005237 [13]. b) Nitrogen sorption isotherms at 77 K of MIP-206 (1062 m^2/g) and **2**@MIP-206$_{0.3}$ post-synthetic (701 m^2/g) composites (filled symbols: adsorption; empty symbols: desorption).

S9. Scanning electron microscopy images of MOFs and composites

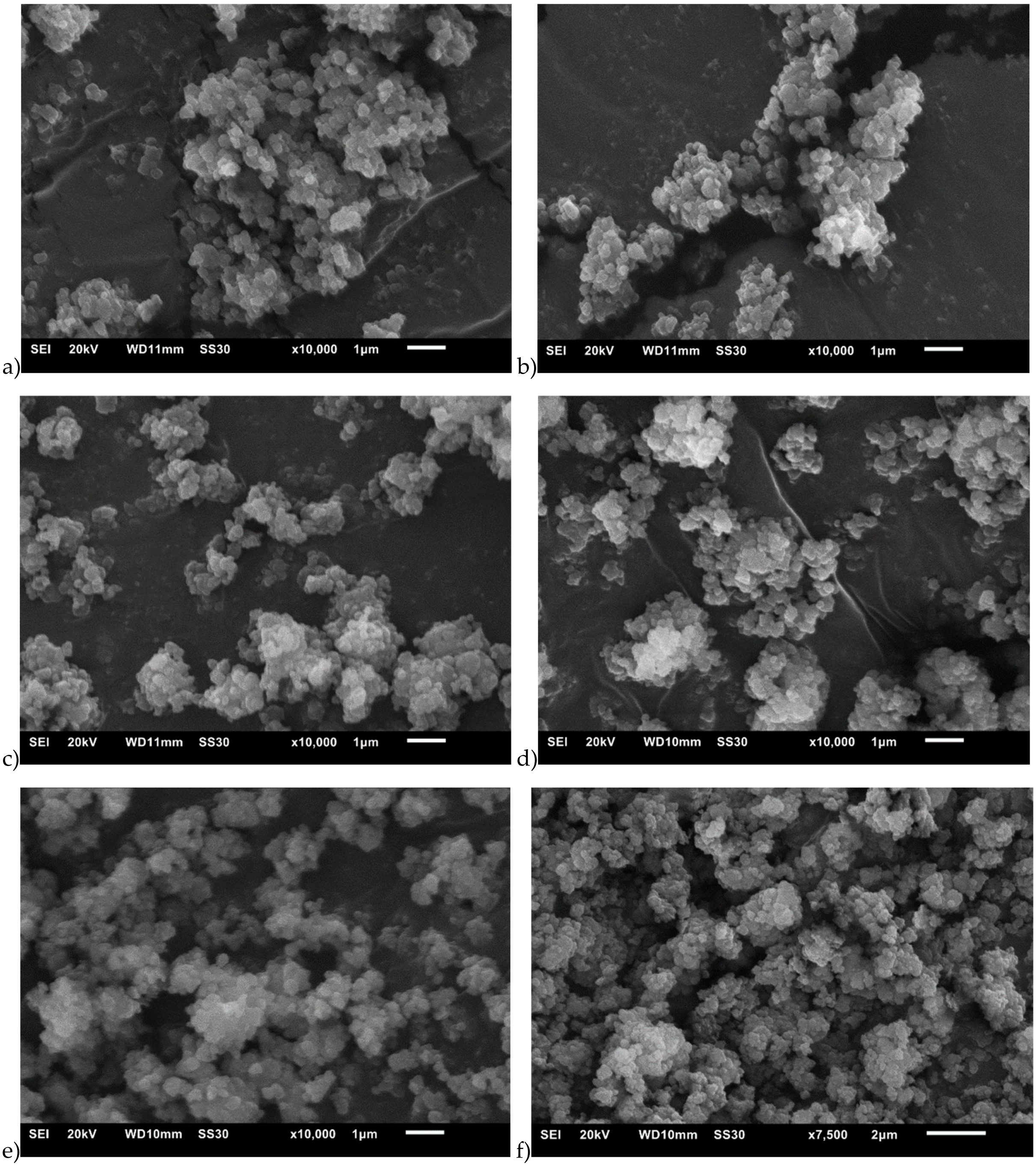

Figure S21. Scanning electron microscopy images of a) neat UiO-66, b) **2**@UiO-66$_{0.44}$ (post-synth.), c) **2**@UiO-66$_{1.1}$ (in situ), d) **2**@UiO-66$_{2.3}$ (in situ), e) **2**@UiO-66$_{4.7}$ (in situ), and f) **2**@UiO-66$_{7.2}$ (in situ).

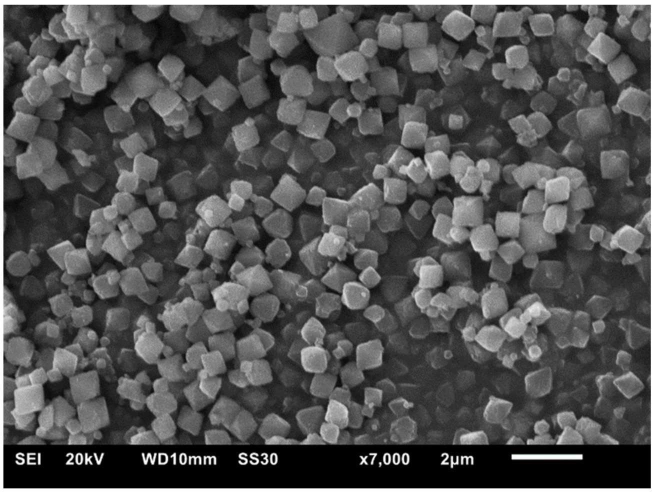

a)

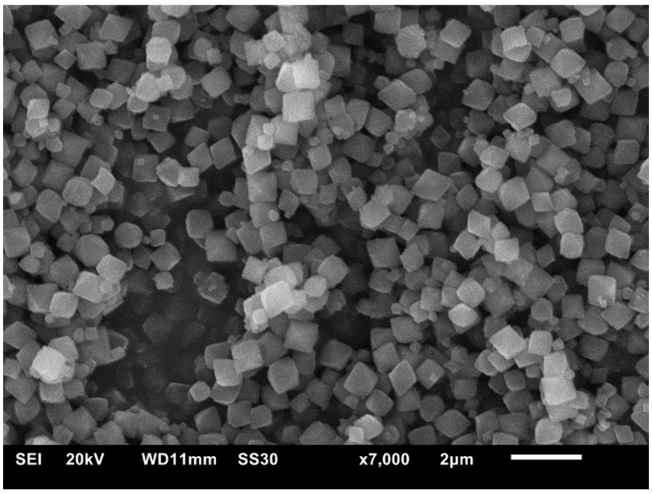

b)

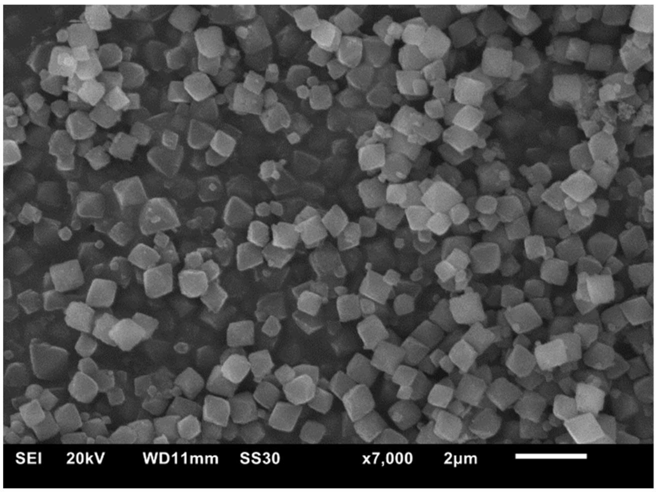

c)

Figure S22. Scanning electron microscopy images of a) neat MOF-808, b) **2**@MOF-808$_{0.57}$ (post-synth.), and c) **2**@MOF-808$_{1.9}$ (in situ).

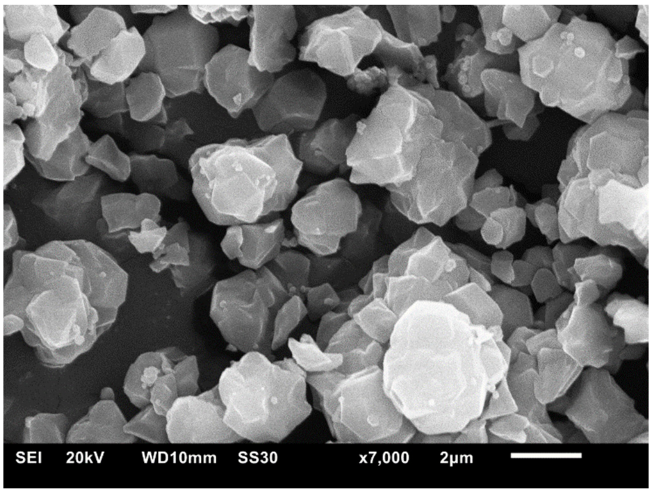

a)

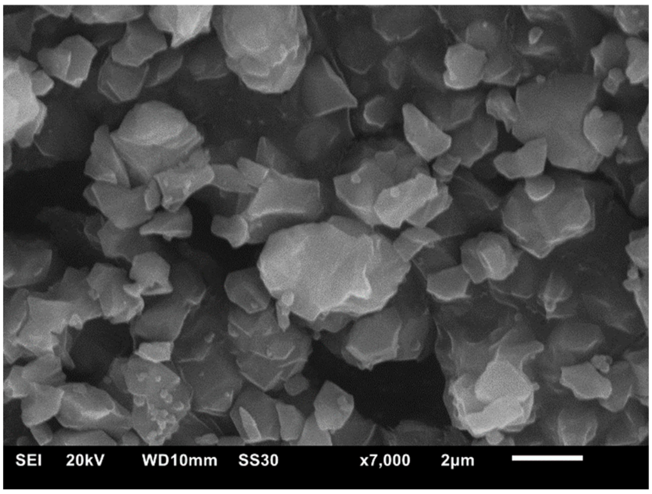

b)

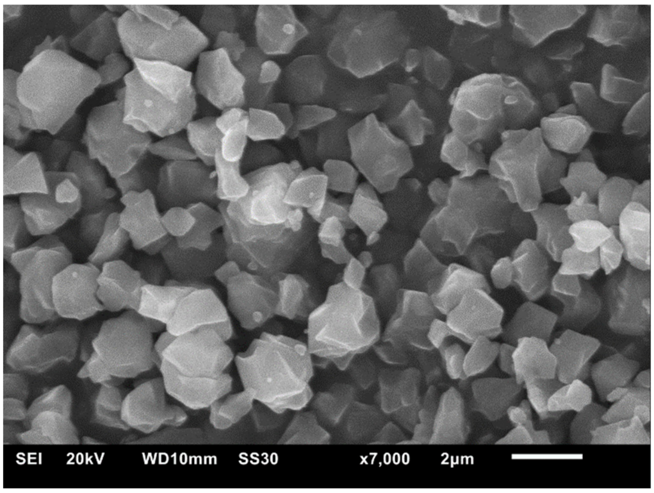

c)

Figure S23. Scanning electron microscopy images of a) neat DUT-67, b) **2**@DUT-$67_{0.51}$ (post-synth.), and c) **2**@DUT-$67_{2.2}$ (in situ).

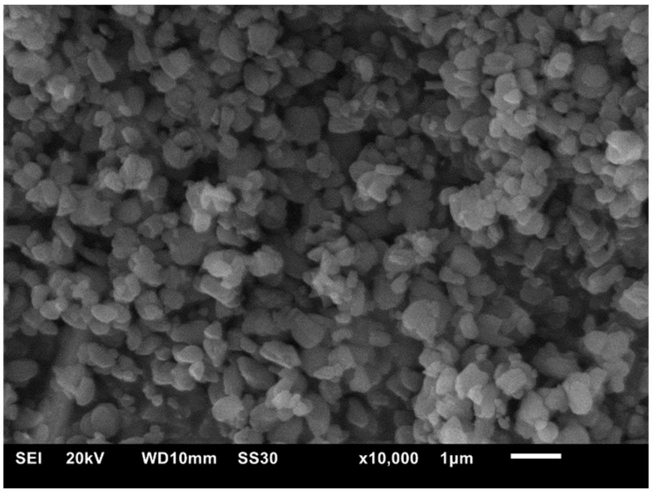

a)

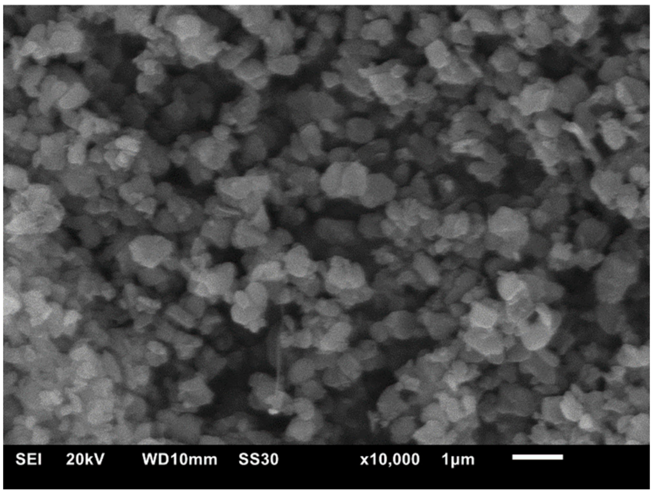

b)

Figure S24. Scanning electron microscopy images of a) neat MIP-206 and b) **2**@MIP-206$_{0.3}$ (post-synth.).

S10. Photophysical properties of all composites

2@UiO-66: Luminescence spectra and emission lifetimes

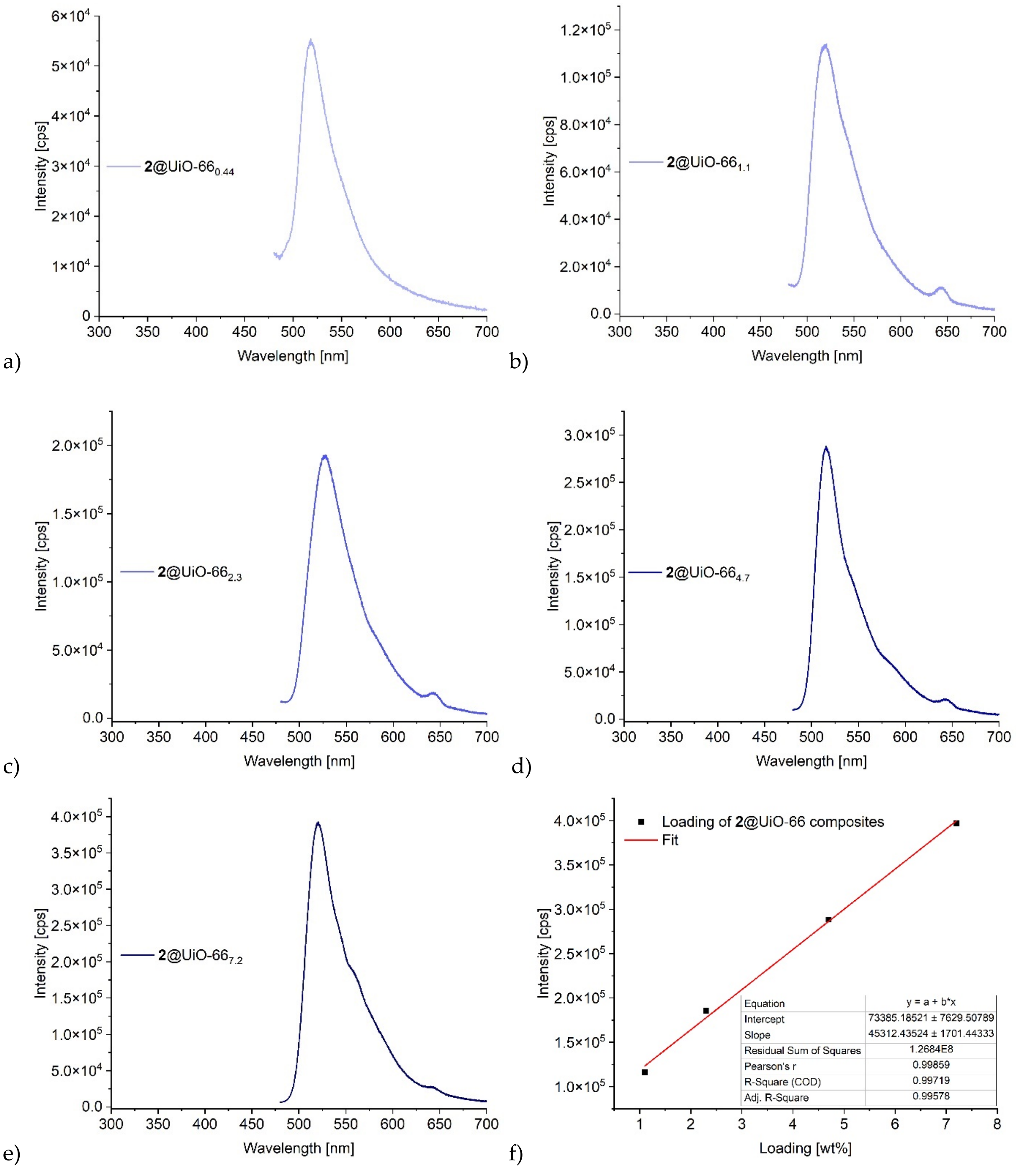

Figure S25. Emission spectra of the **2**@UiO-66 composites: a) 0.44 wt%, b) 1.1 wt%, c) 2.3 wt%, d) 4.7 wt%, and e) 7.2 wt% loading (λ_{exc} = 330 nm) (solid state, reflective setup). f) Linear relationship between intensity and loading of the UiO-66 composites produced in situ.

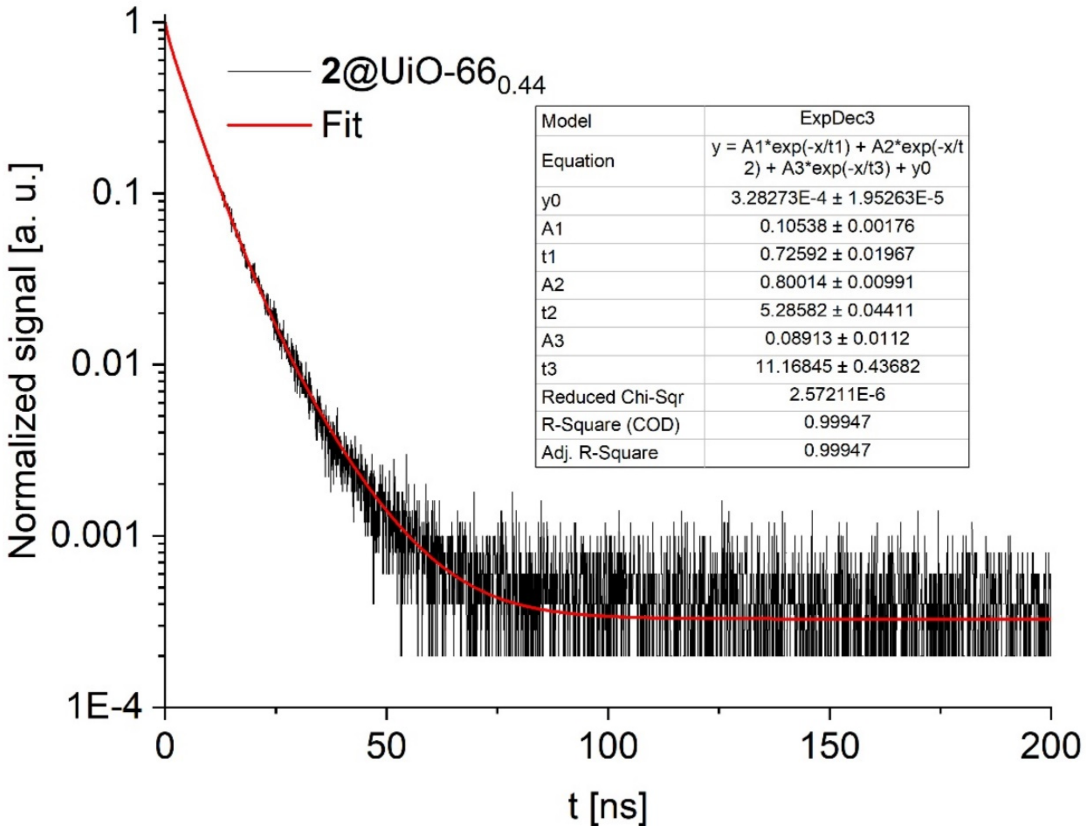

Normalized signal [a. u.]
1
0.1
0.01
0.001
1E-4
0
50
100
150
200
t [ns]
2@UiO-66$_{0.44}$
Fit
Model | ExpDec3
Equation | y = A1*exp(-x/t1) + A2*exp(-x/t2) + A3*exp(-x/t3) + y0
y0 | 3.28273E-4 ± 1.95263E-5
A1 | 0.10538 ± 0.00176
t1 | 0.72592 ± 0.01967
A2 | 0.80014 ± 0.00991
t2 | 5.28582 ± 0.04411
A3 | 0.08913 ± 0.0112
t3 | 11.16845 ± 0.43682
Reduced Chi-Sqr | 2.57211E-6
R-Square (COD) | 0.99947
Adj. R-Square | 0.99947

a)

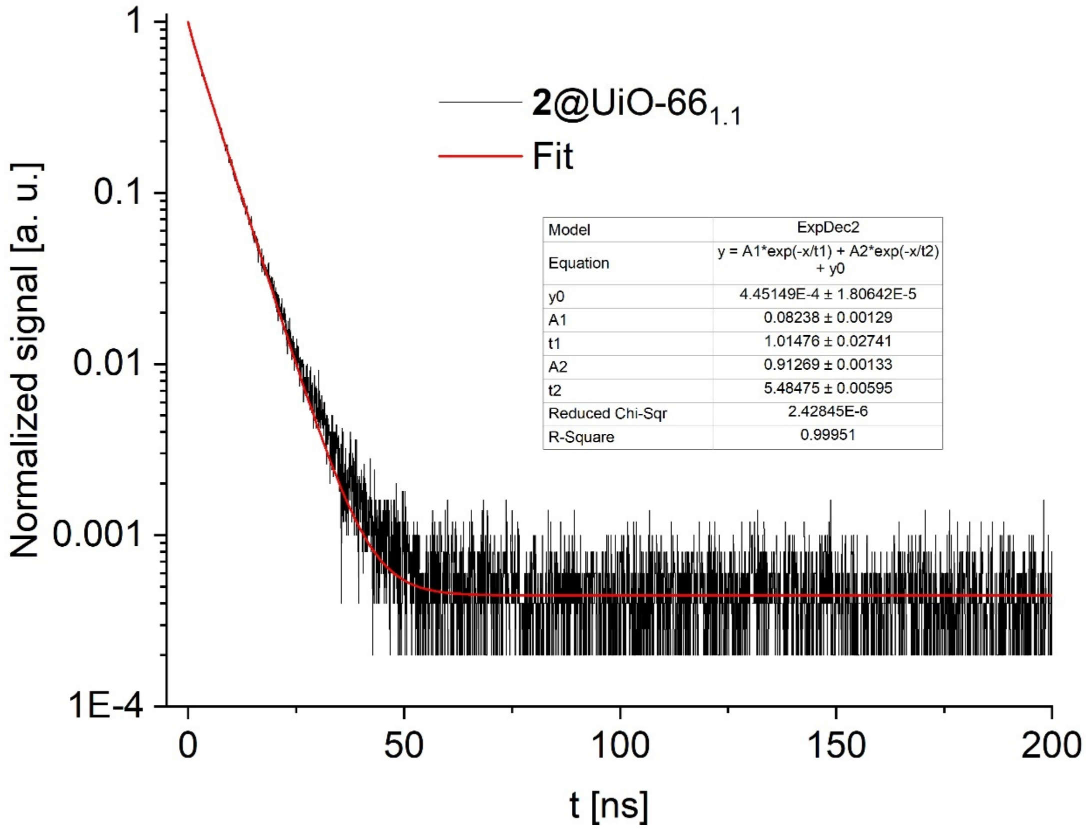

Normalized signal [a. u.]
1
0.1
0.01
0.001
1E-4
0
50
100
150
200
t [ns]
2@UiO-66$_{1.1}$
Fit
Model | ExpDec2
Equation | y = A1*exp(-x/t1) + A2*exp(-x/t2) + y0
y0 | 4.45149E-4 ± 1.80642E-5
A1 | 0.08238 ± 0.00129
t1 | 1.01476 ± 0.02741
A2 | 0.91269 ± 0.00133
t2 | 5.48475 ± 0.00595
Reduced Chi-Sqr | 2.42845E-6
R-Square | 0.99951

b)

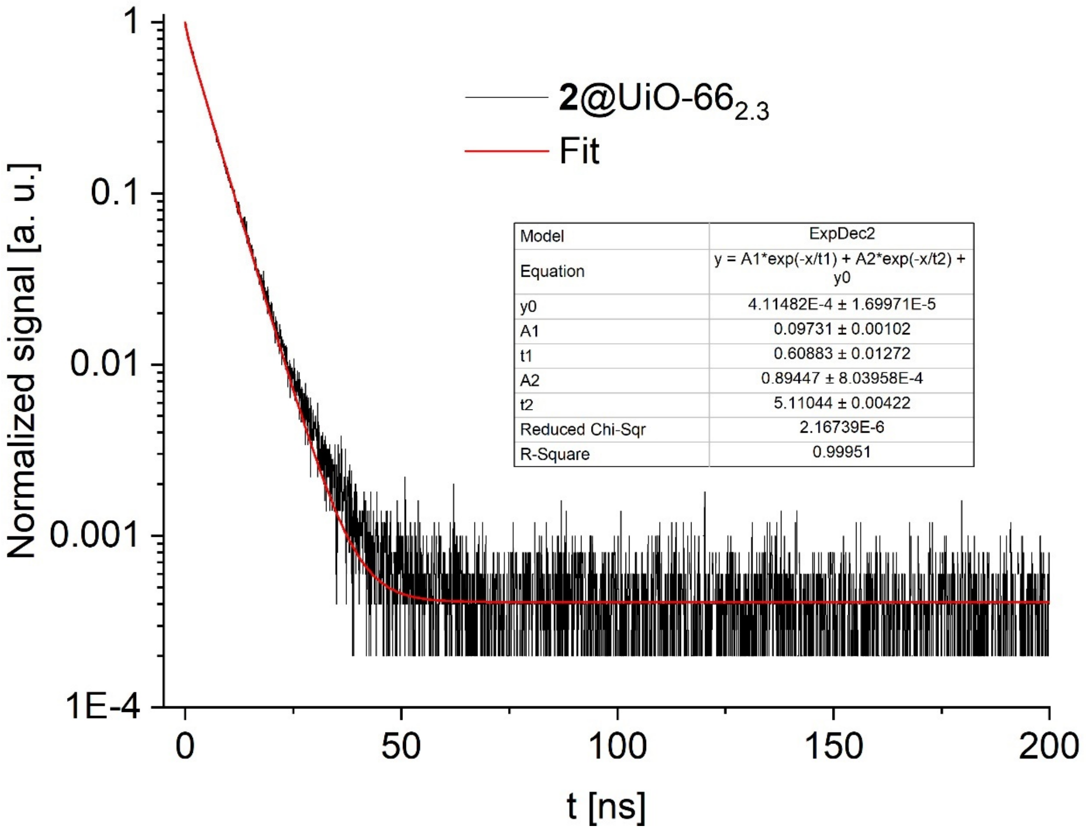

1
0.1
0.01
0.001
1E-4
Normalized signal [a. u.]
0
50
100
150
200
t [ns]
2@UiO-66$_{2.3}$
Fit
Model ExpDec2
Equation y = A1*exp(-x/t1) + A2*exp(-x/t2) + y0
y0 4.11482E-4 ± 1.69971E-5
A1 0.09731 ± 0.00102
t1 0.60883 ± 0.01272
A2 0.89447 ± 8.03958E-4
t2 5.11044 ± 0.00422
Reduced Chi-Sqr 2.16739E-6
R-Square 0.99951

c)

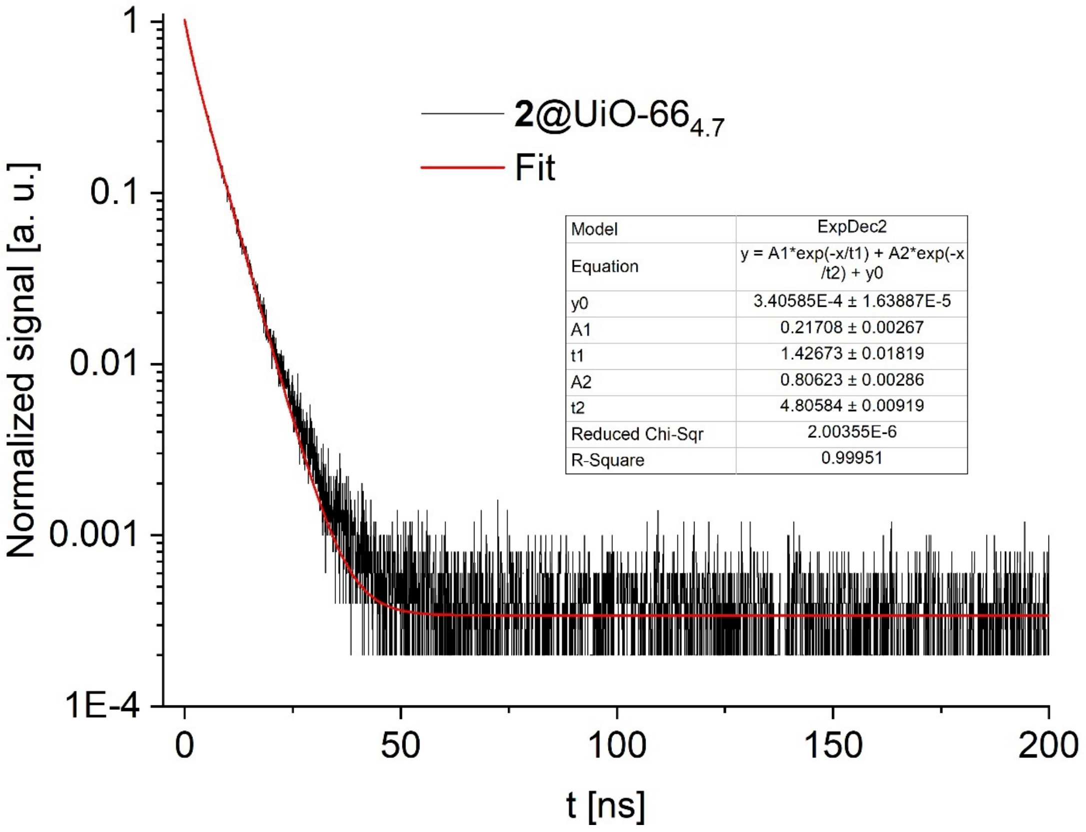

1
0.1
0.01
0.001
1E-4
Normalized signal [a. u.]
0
50
100
150
200
t [ns]
2@UiO-66$_{4.7}$
Fit
Model ExpDec2
Equation y = A1*exp(-x/t1) + A2*exp(-x/t2) + y0
y0 3.40585E-4 ± 1.63887E-5
A1 0.21708 ± 0.00267
t1 1.42673 ± 0.01819
A2 0.80623 ± 0.00286
t2 4.80584 ± 0.00919
Reduced Chi-Sqr 2.00355E-6
R-Square 0.99951

d)

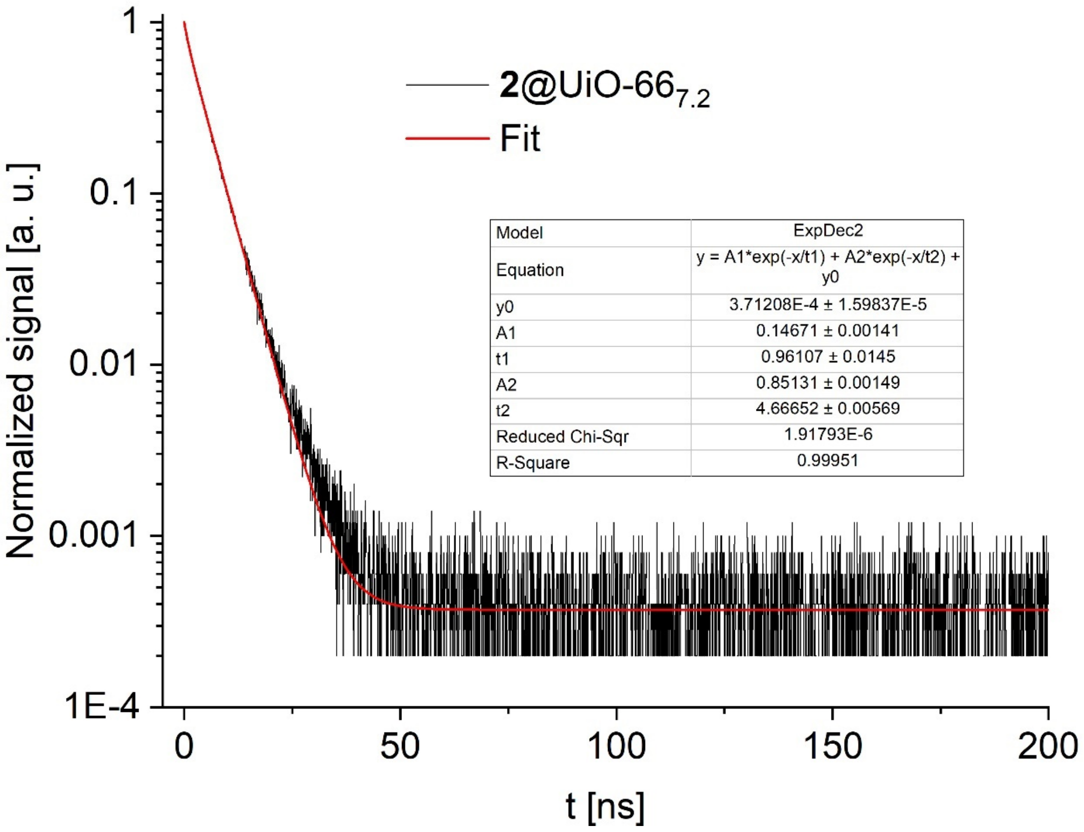

e)

Figure S26. Time-resolved photoluminescence decay (black) of the emission of **2**@UiO-66 composites at 521 nm ± 2 nm. (a) 0.44 wt%, (b) 1.1 wt%, (c) 2.32 wt%, (d) 4.7 wt%, and (e) 7.2 wt% with the respective two- and three-exponential fitting parameters, including pre-exponential factors A_i, lifetimes t_i, and confidence limits (λ_{exc} = 375 nm).

2@MOF-808: Luminescence spectra and emission lifetimes:

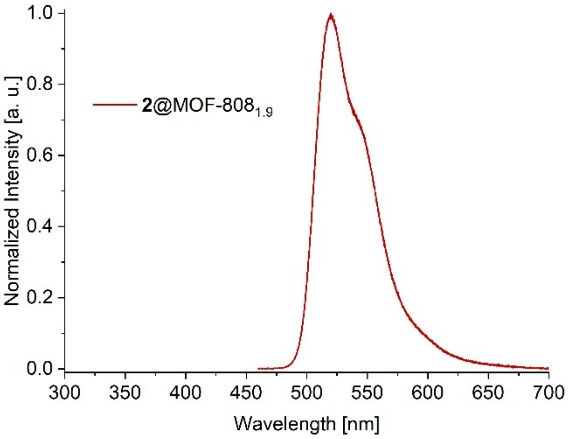

a)

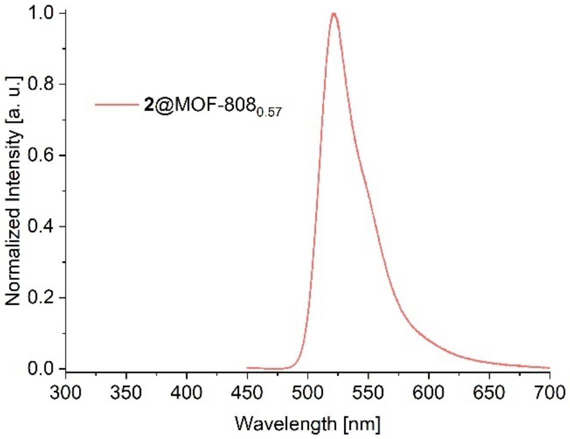

b)

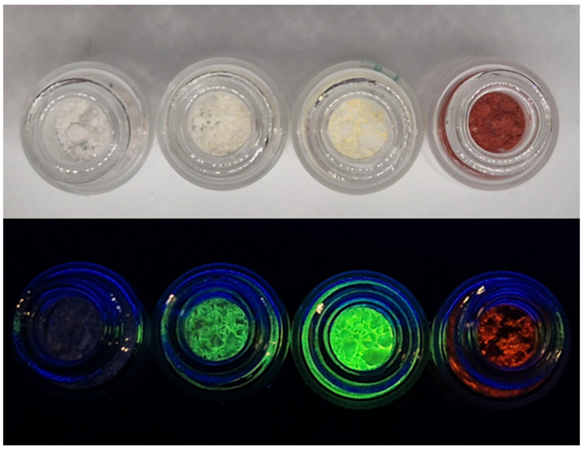

c)

Figure S27. Emission spectra of a) **2**@MOF-808$_{1.9}$ and b) **2**@MOF-808$_{0.57}$ (λ_{exc} = 360 nm) (solid state, reflective setup). c) From left to right: Neat MOF-808, **2**@MOF-808$_{0.57}$, **2**@MOF-808$_{1.9}$ composites and **2** as a solid under daylight (top) and UV-light (λ_{exc} = 365 nm, bottom).

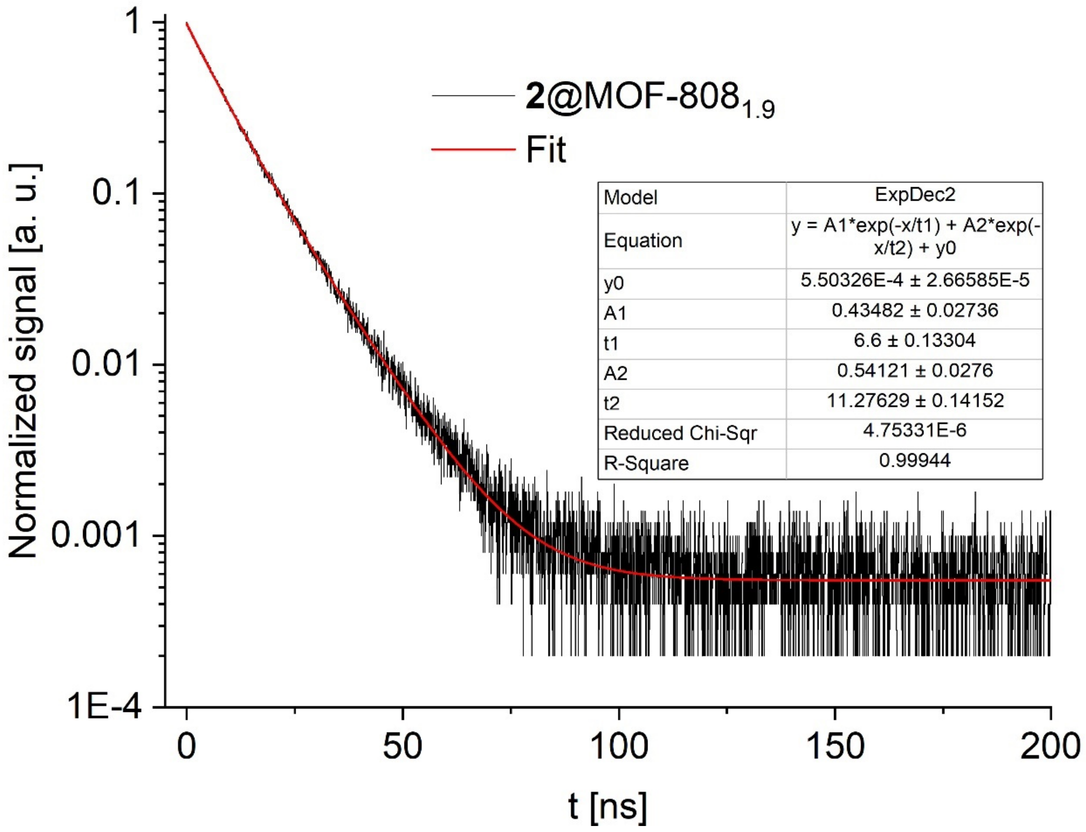

a)

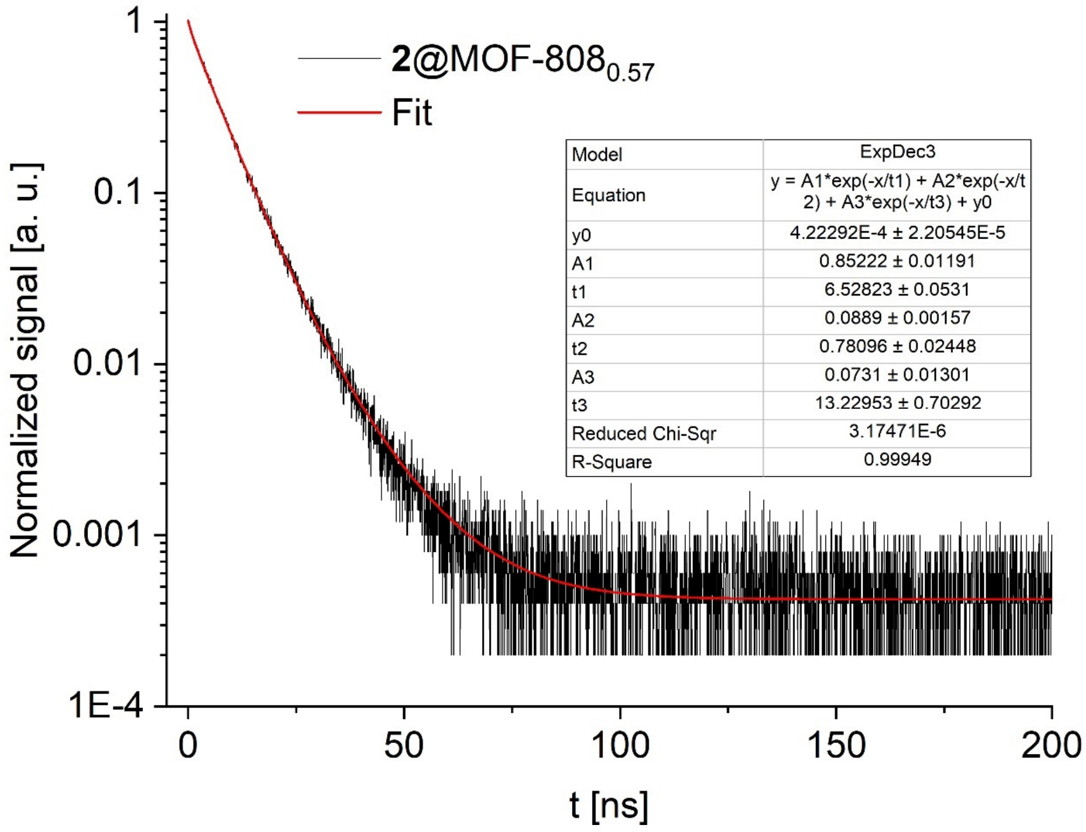

b)

Figure S28. Time-resolved photoluminescence decay (black) of the **2**@MOF-808$_{1.9}$ (a) and **2**@MOF-808$_{0.57}$ (b) composite with the respective two- and three-exponential fitting parameters, including pre-exponential factors A_i, lifetimes t_i, and confidence limits (λ_{exc} = 375 nm).

2@DUT-67: Luminescence spectra and emission lifetimes

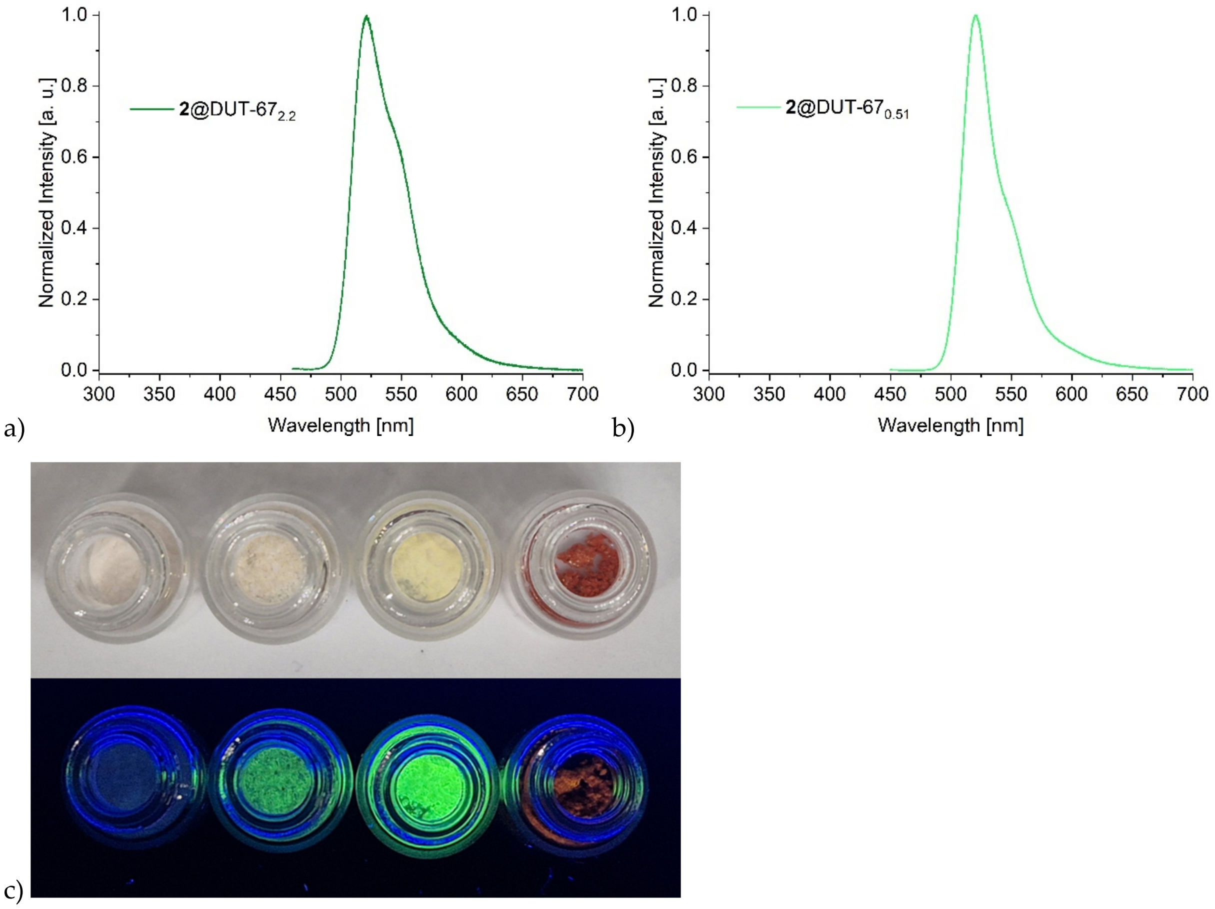

Figure S29. Emission spectra of a) **2**@DUT-67$_{2.2}$ and b) **2**@DUT-67$_{0.51}$ (λ_{exc} = 360 nm) (solid state, reflective setup). c) From left to right: Neat DUT-67, **2**@DUT-67$_{0.51}$, **2**@DUT-67$_{2.2}$ composites and **2** as a solid under daylight (top) and UV-light (λ_{exc} = 365 nm, bottom).

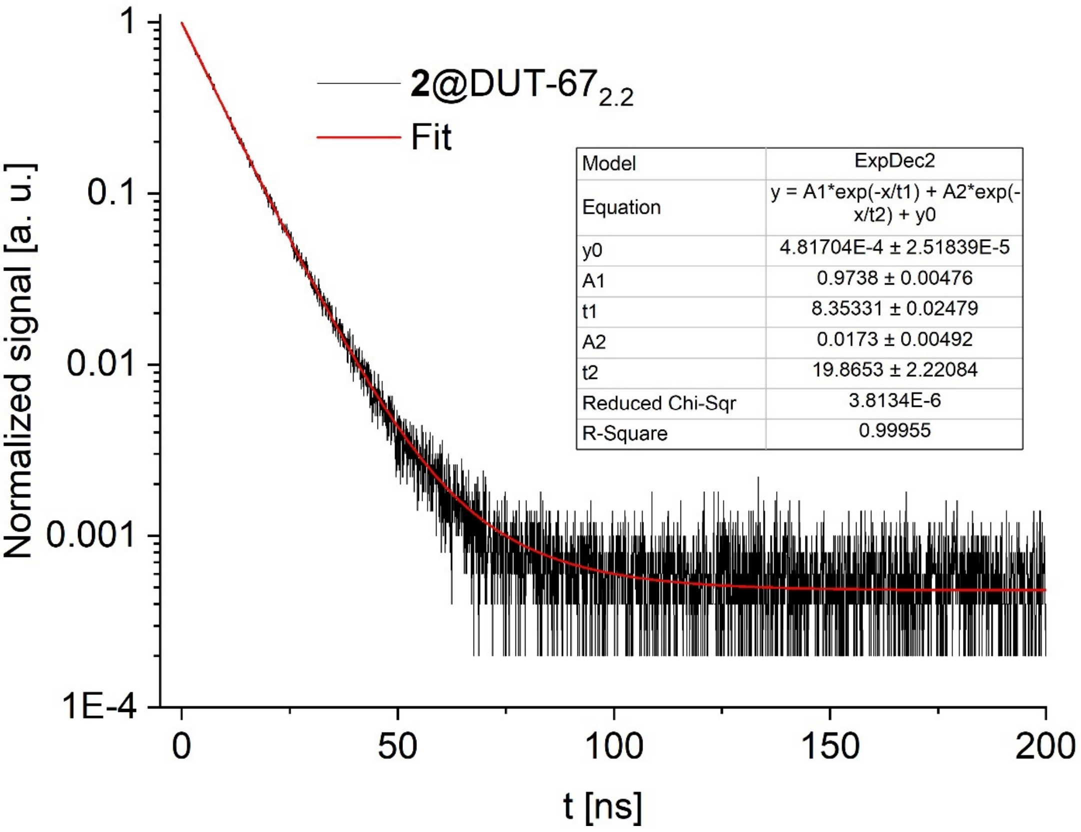

a)

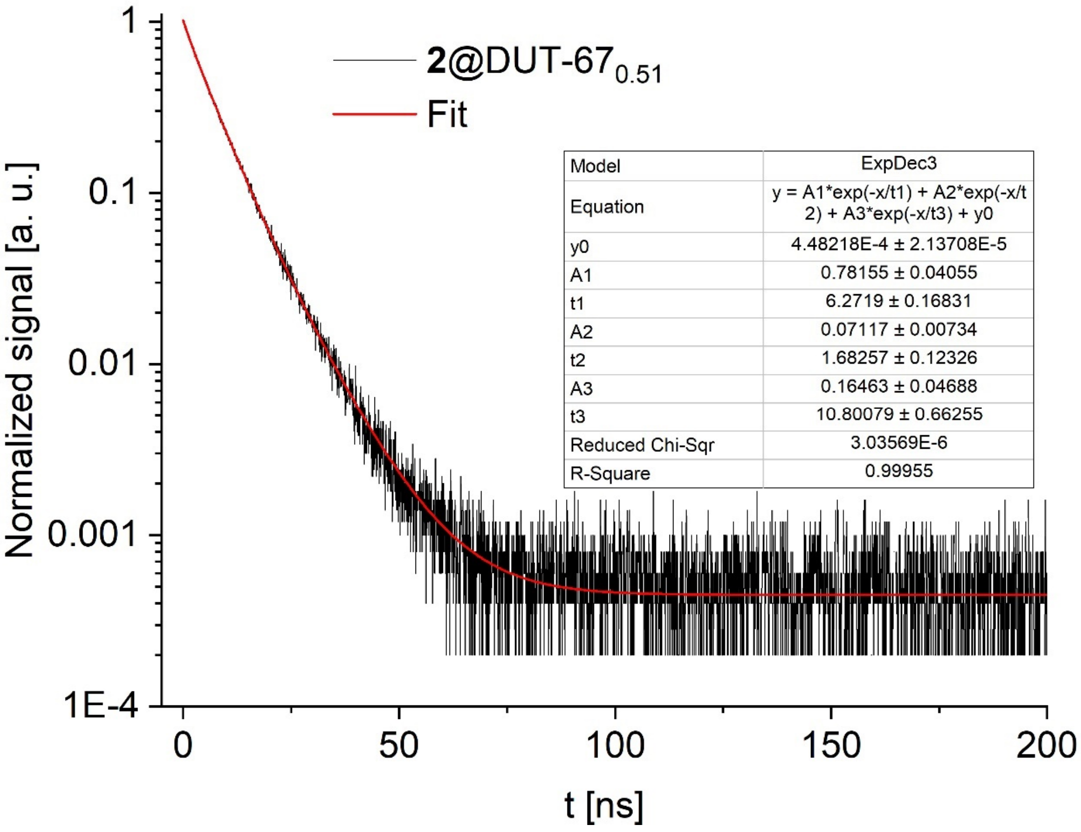

b)

Figure S30. Time-resolved photoluminescence decay (black) of the **2**@DUT-67$_{2.2}$ (a) and **2**@DUT-67$_{0.51}$ (b) composite with the respective two- and three-exponential fitting parameters, including pre-exponential factors A_i, lifetimes t_i, and confidence limits (λ_{exc} = 375 nm).

2@MIP-206: Luminescence spectra and emission lifetimes

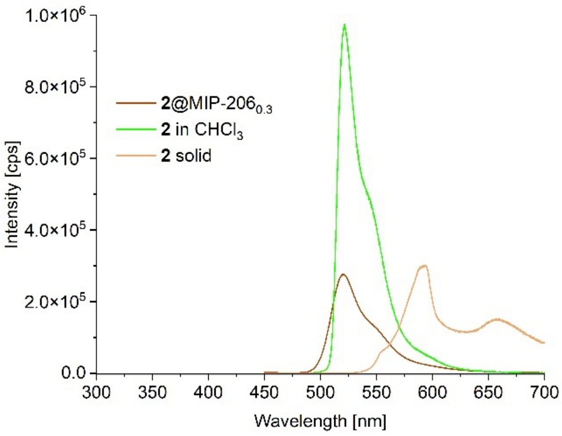

a)

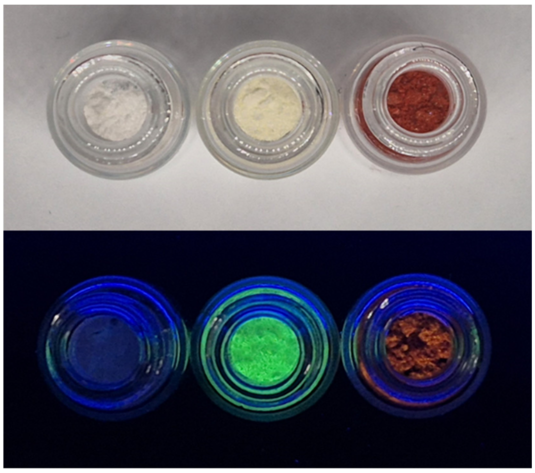

b)

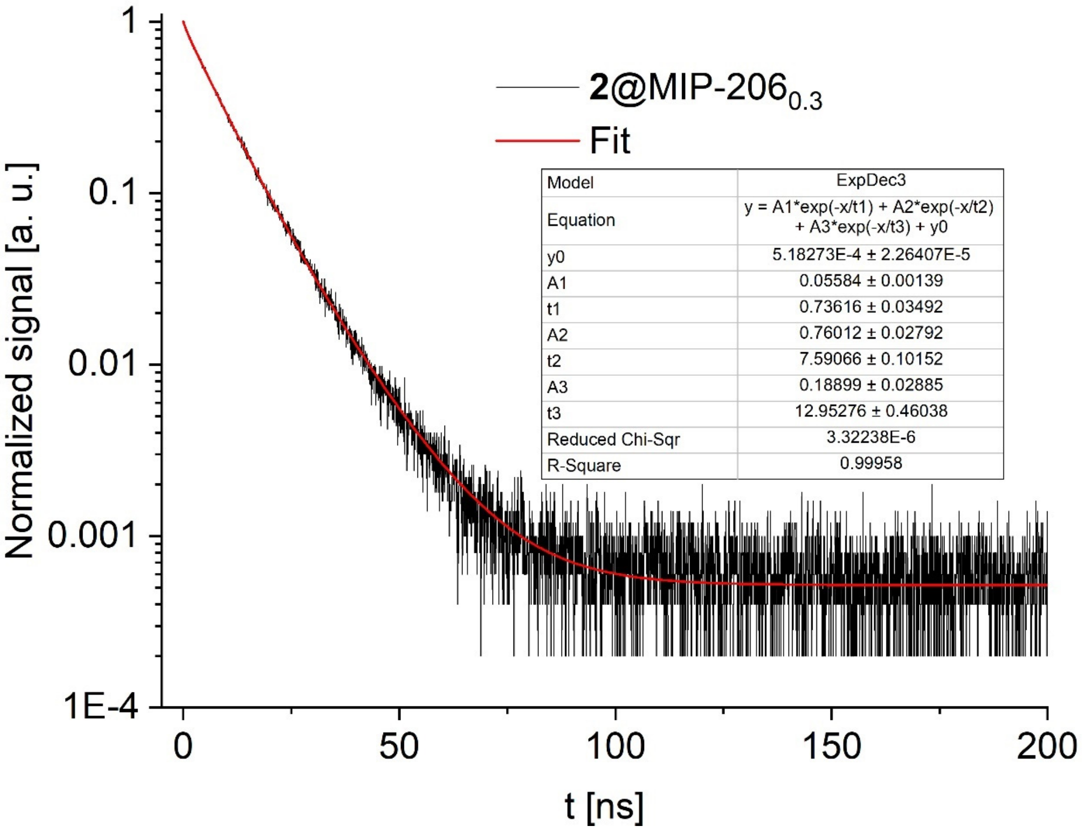

c)

Figure S31. a) Normalized emission spectra of **2**@MIP-206$_{0.3}$, **2** in $CHCl_3$ solution, and **2** as a solid (solid state in a reflective setup). Emission spectra were measured with excitation at 360 nm for **2**@MIP-206$_{0.3}$ and the $CHCl_3$ solution, and at 400 nm for **2** as a solid. b) From left to right: neat MIP-206, post-synthetic encapsulated **2**@MIP-206$_{0.3}$, and **2** as a solid both under daylight (top) and under UV-light (λ_{exc} = 365 nm, bottom). c) Time-resolved photoluminescence decay (black) of the emission of the **2**@MIP-206$_{0.3}$ composite at 521 nm with the respective three-exponential fitting parameters, including pre-exponential factors A_1, A_2, and A_3, lifetimes t_1, t_2, and t_3, and confidence limits (λ_{exc} = 375 nm).

Table S6. Photophysical data for **2** as a solid and in solution, and the **2**@MIP-206 composite.

Compound	$\lambda_{F, max}$ [nm] [a]	τ_1 (x_1), τ_2 (x_2), τ_3 (x_3) [ns] [b]	τ_x [ns] [b]	Φ_F [%] [c]
**2** solid	594/659	0.6 (0.74), 2.8 (0.29)	1.2	9
**2** in $CHCl_3$ (0.5 mmol/L)	510	4.8 (1)	4.8	89 [d]
**2**@MIP-206$_{0.3}$ (post-synth.)	521	0.7 (0.05), 7.6 (0.7), 12.9 (0.2)	7.9	47

[a] Wavelength of the fluorescence maximum (λ_{exc} = 360 nm). [b] Fluorescence lifetime (λ_{exc} = 375 nm): τ_i (x_i) species i lifetime (fraction), τ_x species-weighted average lifetime. [c] Fluorescence quantum yield. [d] Measured in THF taken from the literature [2].

S11. References

1. Caruso, E.; Gariboldi, M.; Sangion, A.; Gramatica, P.; Banfi, S. Synthesis, photodynamic activity, and quantitative structure-activity relationship modelling of a series of BODIPYs. *J. Photochem. Photobiol. B* **2017**, *167*, 269-281, doi:10.1016/j.jphotobiol.2017.01.012.
2. Nguyen, A.L.; Wang, M.; Bobadova-Parvanova, P.; Do, Q.; Zhou, Z.; Fronczek, F.R.; Smith, K.M.; Vicente, M.G.H. Synthesis and properties ofB-cyano-BODIPYs. *J. Porphyr. Phthalocyanines* **2017**, *20*, 1409-1419, doi:10.1142/s108842461650125x.
3. Katz, M.J.; Brown, Z.J.; Colon, Y.J.; Siu, P.W.; Scheidt, K.A.; Snurr, R.Q.; Hupp, J.T.; Farha, O.K. A facile synthesis of UiO-66, UiO-67 and their derivatives. *Chem. Commun.* **2013**, *49*, 9449-9451, doi:10.1039/c3cc46105j.
4. Guillerm, V.; Gross, S.; Serre, C.; Devic, T.; Bauer, M.; Ferey, G. A zirconium methacrylate oxocluster as precursor for the low-temperature synthesis of porous zirconium(IV) dicarboxylates. *Chem. Commun.* **2010**, *46*, 767-769, doi:10.1039/b914919h.
5. Garai, M.; Yavuz, C.T. Robust Mesoporous Zr-MOF with Pd Nanoparticles for Formic-Acid-Based Chemical Hydrogen Storage. *Matter* **2021**, *4*, 10-12, doi:10.1016/j.matt.2020.12.011.
6. Valverde, A.; Tovar, G.I.; Rio-López, N.A.; Torres, D.; Rosales, M.; Wuttke, S.; Fidalgo-Marijuan, A.; Porro, J.M.; Jiménez-Ruiz, M.; García Sakai, V.; et al. Designing Metal-Chelator-like Traps by Encoding Amino Acids in Zirconium-Based Metal–Organic Frameworks. *Chem. Mater.* **2022**, *34*, 9666-9684, doi:10.1021/acs.chemmater.2c02431.
7. Winters, W.M.W.; Zhou, C.; Hou, J.; Diaz-Lopez, M.; Bennett, T.D.; Yue, Y. Order-to-Disorder Transition in a Zirconium-Based Metal–Organic Framework. *Chem. Mater.* **2024**, *36*, 8400-8411, doi:10.1021/acs.chemmater.4c01460.
8. D'Amato, R.; Bondi, R.; Moghdad, I.; Marmottini, F.; McPherson, M.J.; Naïli, H.; Taddei, M.; Costantino, F. "Shake 'n Bake" Route to Functionalized Zr-UiO-66 Metal-Organic Frameworks. *Inorg. Chem.* **2021**, *60*, 14294-14301, doi:10.1021/acs.inorgchem.1c01839.
9. Valenzano, L.; Civalleri, B.; Chavan, S.; Bordiga, S.; Nilsen, M.H.; Jakobsen, S.; Lillerud, K.P.; Lamberti, C. Disclosing the Complex Structure of UiO-66 Metal Organic Framework: A Synergic Combination of Experiment and Theory. *Chem. Mater.* **2011**, *23*, 1700-1718, doi:10.1021/cm1022882.
10. Furukawa, H.; Gandara, F.; Zhang, Y.B.; Jiang, J.; Queen, W.L.; Hudson, M.R.; Yaghi, O.M. Water adsorption in porous metal-organic frameworks and related materials. *J. Am. Chem. Soc.* **2014**, *136*, 4369-4381, doi:10.1021/ja500330a.
11. Reinsch, H.; Waitschat, S.; Chavan, S.M.; Lillerud, K.P.; Stock, N. A Facile “Green” Route for Scalable Batch Production and Continuous Synthesis of Zirconium MOFs. *Eur. J. Inorg. Chem.* **2016**, *2016*, 4490-4498, doi:10.1002/ejic.201600295.
12. Bon, V.; Senkovska, I.; Baburin, I.A.; Kaskel, S. Zr- and Hf-Based Metal–Organic Frameworks: Tracking Down the Polymorphism. *Cryst. Growth Des.* **2013**, *13*, 1231-1237, doi:10.1021/cg301691d.
13. Wang, S.; Chen, L.; Wahiduzzaman, M.; Tissot, A.; Zhou, L.; Ibarra, I.A.; Gutiérrez-Alejandre, A.; Lee, J.S.; Chang, J.-S.; Liu, Z.; et al. A Mesoporous Zirconium-Isophthalate Multifunctional Platform. *Matter* **2021**, *4*, 182-194, doi:10.1016/j.matt.2020.10.009.
